# Supplementary material for: Characteristics of functional enrichment and gene expression level of human putative transcriptional target genes
Source: BMC Genomics. 2018 Jan 19;19(Suppl 1):957. doi: 10.1186/s12864-017-4339-5 (PMC5780744; doi:10.1186/s12864-017-4339-5)
Supplement: Additional file 1: — Supplementary Data. Supplementary results, figures and tables. (PDF 3383 kb) [file 12864_2017_4339_MOESM1_ESM.pdf]

Characteristics of functional enrichment and gene expression level of human putative transcriptional target genes

Naoki Osato

Department of Bioinformatic Engineering, Graduate School of Information Science and Technology, Osaka University, Osaka, Japan

Table of contents

Results	P.2
Figure S1.	P.3 – P.5
Figure S2.	P.6
Table S1.	P.7
Table S2.	P.8
Table S3.	P.9
Table S4.	P.10 – P.18
Table S5.	P.19
Table S6.	P.20 – P.22
Table S7.	P.23 – P.26
Table S8.	P.27 – P.28
Table S9.	P.29
Table S10.	P.30 – P.31
Table S11.	P.32 – P.34
Table S12.	P.35
Table S13.	P.35 – P.37
Table S14.	P.37
Table S15.	P.38 – P.41
Table S16.	P.42
Table S17.	P.42 – P.43
Table S18.	P.44 – P.46
Table S19.	P.47

Supplementary Data

Results

Effect of CTCF-binding sites on functional enrichments

Differences in functional enrichments obtained using promoter and extended regions for enhancer-promoter association (EPA) (association rule 4) *versus* EPA (association rule 4) that were shortened at genomic locations of forward–reverse orientation of CTCF-binding sites (association rule 4) were examined using the functional enrichments of Pathway Commons (Table 6 and S18). A comparison of 325 and 225 functional enrichments using the EPA and the EPA shortened at forward–reverse orientation of CTCF-binding sites, respectively, in monocytes revealed that 166 (51% in EPA, 74% in EPA shortened at forward–reverse orientation of CTCF-binding sites) of them were common. For example, Interferon gamma signaling, IL4 (Interleukin-4, a kind of cytokine)-mediated signaling events, and Immune System were enriched using forward–reverse orientation of CTCF-binding sites as opposed to without them. The comparison of 412 (EPA) and 229 (EPA shortened at forward–reverse orientation of CTCF-binding sites) functional enrichments in $CD4^+$ T cells revealed that 181 of them (44% in EPA, 79% in EPA shortened at forward–reverse orientation of CTCF-binding sites) were common. TCR (T-cell receptor) signaling in naive $CD8^+$ T cells, JNK (c-Jun NH2-terminal kinase) signaling in the $CD4^+$ TCR pathway, and TCR signaling were enriched using CTCF-binding sites. The comparison of 358 (EPA) and 205 (EPA shortened at forward–reverse orientation of CTCF-binding sites) functional enrichments in $CD20^+$ B cells revealed that 144 of them (40% in EPA, 70% in EPA shortened at forward–reverse orientation of CTCF-binding sites) were common. Syndecan-4-mediated signaling events, Interleukin-1 signaling, and Interferon gamma signaling were enriched using CTCF-binding sites. Journal papers showed that the top five frequent functional enrichments were related to the cellular functions in the three cell types (Table 6). These results showed that new functional enrichments related to cellular functions were identified using forward–reverse orientation of CTCF-binding sites.

Supplementary Figures

Figure S1.

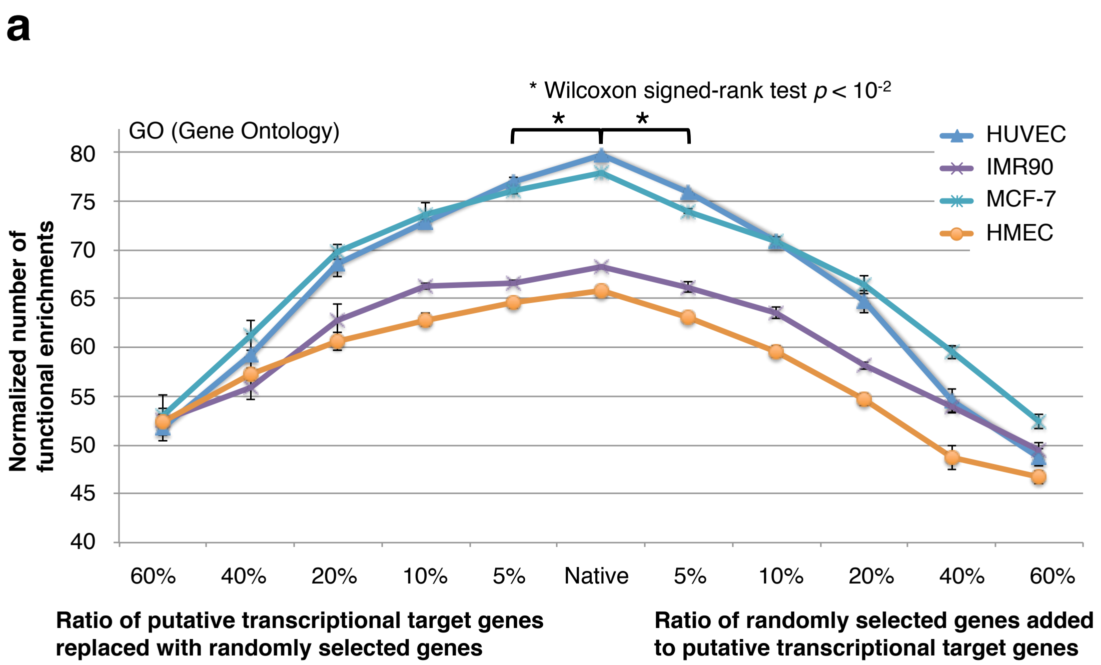

Figure S1. Effect of randomly selected genes on functional enrichments. (a) Effect of randomly selected genes on functional enrichments using DNase-DGF data. Transcriptional target genes were predicted using DNase-DGF data in human HUVEC, IMR90, MCF-7 and HMEC. To test whether slight changes of transcriptional target genes were reflected in the normalized number of their functional enrichments, the ratio of randomly selected genes in the target genes of each TF was changed between 5% and 60%. In the left part of the graphs, randomly selected genes were replaced with the target genes where the total number of target genes was unchanged. In the right part of the graphs, randomly selected genes were added to the target genes where the total number of target genes was increased. The result of Gene Ontology annotation was shown. Native target genes showed the most functional enrichments in the four cell types.

b

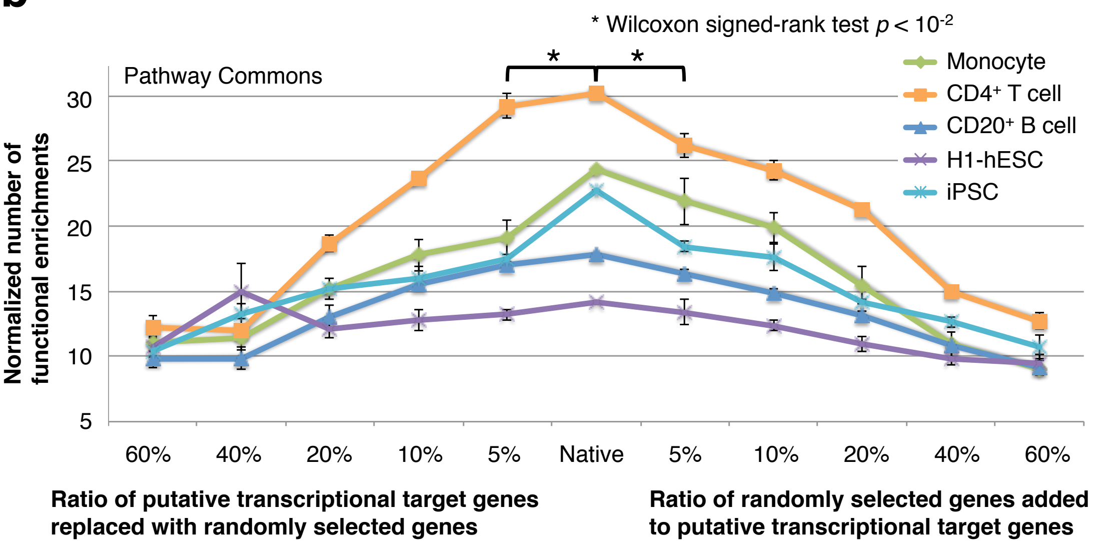

c

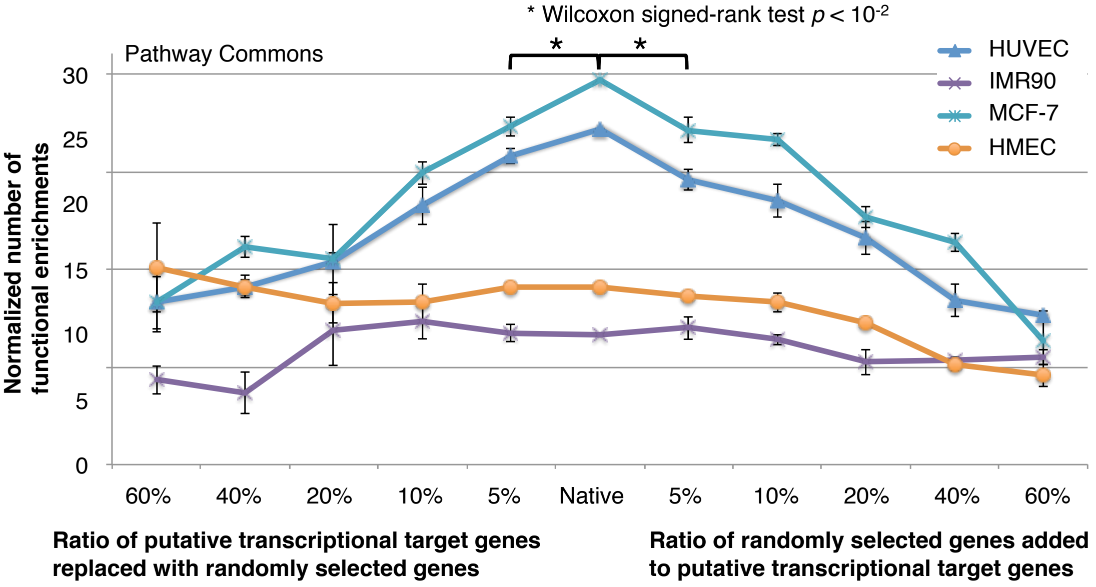

(b) Effect of randomly selected genes on functional enrichments of transcriptional target genes predicted using DNase-DGF data of human monocytes, $CD4^+$ T, $CD20^+$ B cells, H1-hESC and iPSC. **(c)** Effect of randomly selected genes on functional enrichments of transcriptional target genes predicted using DNase-DGF data of human HUVEC, IMR90, MCF-7 and HMEC. The results of Pathway Commons annotation were shown. Native target genes showed the most functional enrichments in most cell types.

d

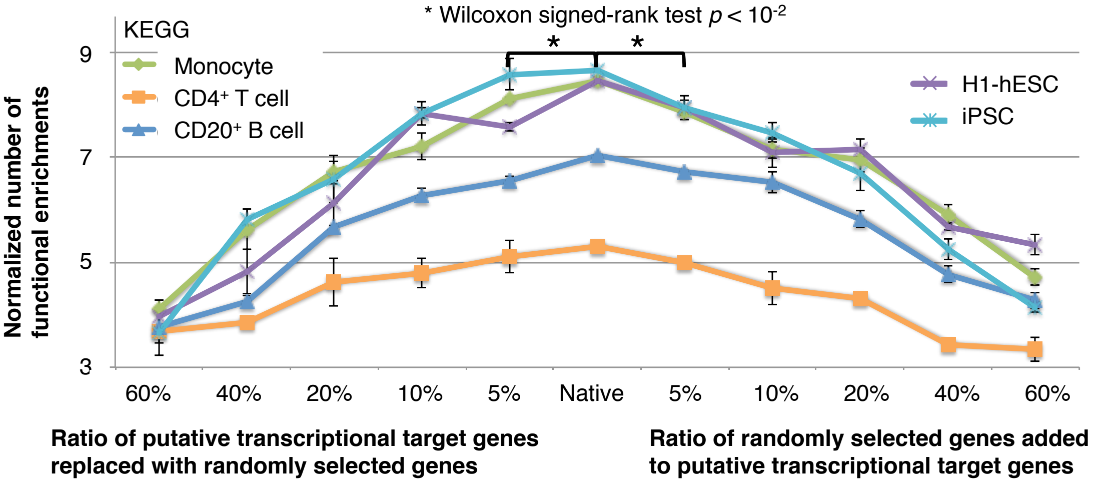

e

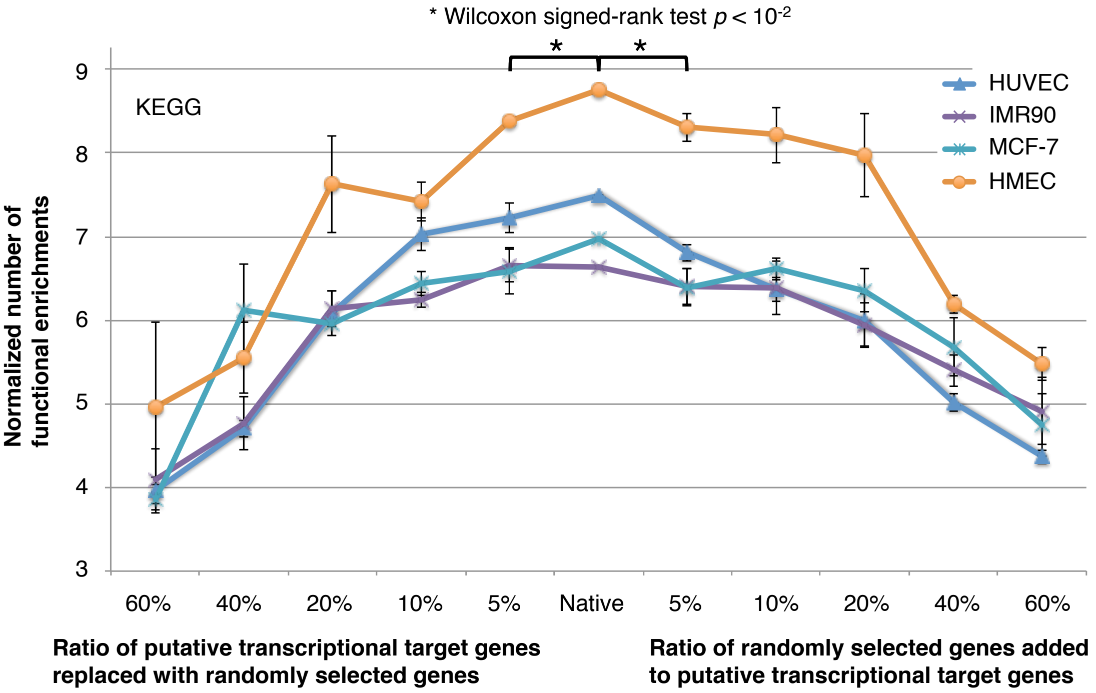

(d) Effect of randomly selected genes on functional enrichments of transcriptional target genes predicted using DNase-DGF data of monocytes, CD4+ T, CD20+ B cells, H1-hESC and iPSC. **(e)** Effect of randomly selected genes on functional enrichments of transcriptional target genes predicted using DNase-DGF data of human HUVEC, IMR90, MCF-7 and HMEC. The results of KEGG annotation were shown. Native target genes showed the most functional enrichments in the nine cell types.

Figure S2.

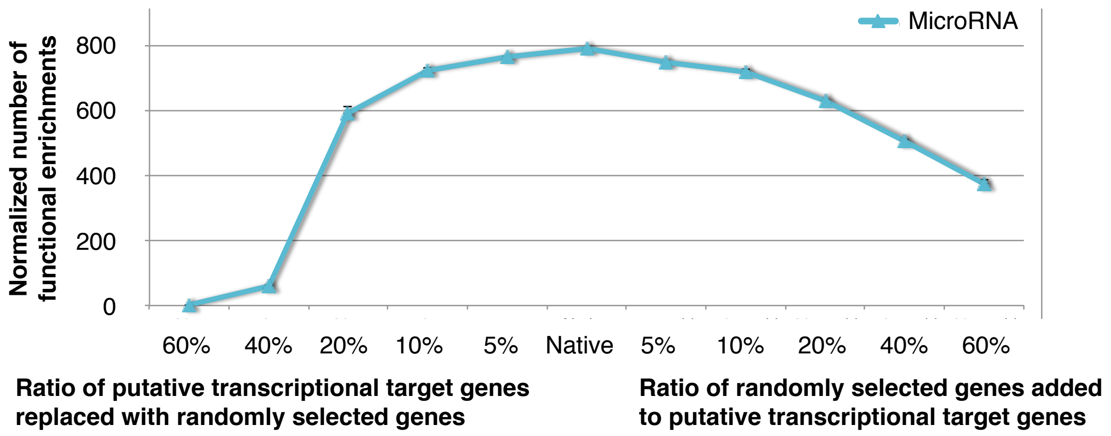

Figure 2. Effect of randomly selected genes on functional enrichments. Effect of randomly selected genes on functional enrichments using ChIP-seq data. Transcriptional target genes were predicted using ChIP-seq data of 19 TF in H1-hESC. The result of target genes of microRNAs was shown. Native target genes showed the most functional enrichments.

Supplementary Tables

Table S1. Number of functional enrichments and unique functional enrichments of putative transcriptional target genes.

Table S1. Number of functional enrichments and unique functional enrichemnts of putative transcriptional target genes.

Number of functional enrichments of putative trancriptional target genes.

	KEGG	TF Targets	CTD Ontology	GO Slim	GO	Pathway Commons	BioMarkers	MicroRNA	Domains	WikiPathways
Monocyte	349	107	209	114	2,902	1,005	42	451	1,202	242
CD4⁺ T cell	317	135	278	77	4,077	1,806	47	754	1,401	405
CD20⁺ B cell	323	103	170	88	2,778	821	39	948	950	288

Number of unique functional enrichments of gene expression information alone and putative transcriptional target genes.
*** Wilcoxon signed-rank test $p < 0.01$ (except for MicroRNA and Domains)**

	KEGG	TF Targets	CTD Ontology	GO Slim	GO	Pathway Commons	BioMarkers	MicroRNA	Domains	WikiPathways
**Gene expression information alone, independent of transcirptional target genes.**										
Monocyte	43	0	35	11	237	101	7	314	404	58
CD4⁺ T cell	47	0	19	9	301	165	9	136	397	81
CD20⁺ B cell	42	0	27	12	239	247	6	370	409	65
**Putative transcriptional target genes.**										
Monocyte	95	16	127	12	1,271	242	17	97	303	105
CD4⁺ T cell	105	26	146	23	1,654	415	24	224	585	133
CD20⁺ B cell	93	23	96	23	1,192	329	16	231	397	106

Z-Score: −2 −1 0 1 2

Table S2. Normalized number of functional enrichments of putative transcriptional target genes using promoter and extended regions for enhancer-promoter association.

*** Wilcoxon signed-rank test $p < 0.05$**

Table S2. Normalized number of functional enrichments of putative transcriptional target genes using promoter and extended regions for enhancer-promoter association.
*** Wilcoxon signed-rank test $p < 0.05$**

	KEGG	CTD Ontology	GO Slim	GO	Pathway Commons	Domains	Wiki Pathways
**Monocyte**							
Promoter	8.46	5.07	2.76	70.34	24.36	29.13	5.87
Association rule 1	10.44	6.48	2.01	133.54	44.75	42.19	11.10
Association rule 2	9.03	6.29	1.45	125.13	38.69	40.17	8.30
Association rule 3	8.06	5.29	1.37	106.60	24.25	38.96	7.62
Association rule 4	11.47	8.22	2.46	164.18	71.42	47.85	12.78
**$CD4^+$ T cell**							
Promoter	5.30	4.64	1.29	68.11	30.17	23.41	6.77
Association rule 1	13.60	7.07	2.74	142.40	84.51	43.78	13.65
Association rule 2	13.57	6.69	3.05	141.15	86.36	46.33	12.02
Association rule 3	12.40	5.89	2.50	115.76	68.84	41.85	10.00
Association rule 4	16.40	7.86	4.03	177.55	108.08	53.86	16.72
**$CD20^+$ B cell**							
Promoter	7.02	3.70	1.91	60.39	17.85	20.65	6.26
Association rule 1	8.88	6.21	2.59	104.55	59.32	34.34	8.29
Association rule 2	8.60	5.32	1.55	105.34	57.31	38.05	9.95
Association rule 3	9.01	5.28	1.42	88.85	26.49	35.17	8.26
Association rule 4	9.95	6.62	3.07	134.46	90.99	41.30	10.67
**HUVEC**							
Promoter	7.50	5.74	1.34	79.71	22.21	22.40	4.59
Association rule 1	11.62	8.95	3.74	160.52	37.11	65.15	8.88
Association rule 2	11.87	8.77	4.76	176.26	36.86	74.71	8.66
Association rule 3	9.84	7.70	4.55	154.06	34.29	73.98	7.74
Association rule 4	13.46	8.99	5.08	191.73	59.10	82.65	10.13
**IMR90**							
Promoter	6.63	5.21	2.43	68.31	11.64	25.50	5.47
Association rule 1	9.63	6.78	2.85	122.38	24.89	65.39	8.03
Association rule 2	9.32	6.56	1.43	115.31	16.36	60.36	7.47
Association rule 3	9.59	5.63	1.55	99.10	15.39	54.82	5.82
Association rule 4	10.55	7.68	3.76	142.10	30.05	74.86	8.78
**MCF-7**							
Promoter	6.97	5.43	1.69	77.88	24.71	25.99	8.77
Association rule 1	9.45	6.26	2.03	121.84	22.38	55.19	9.86
Association rule 2	9.44	6.18	0.89	111.77	21.59	53.90	7.97
Association rule 3	9.83	5.13	1.12	100.35	18.96	55.30	7.37
Association rule 4	10.80	7.38	2.77	149.64	31.20	62.40	11.07
**HMEC**							
Promoter	8.76	5.03	1.53	65.80	14.07	26.52	6.70
Association rule 1	10.88	7.04	1.96	138.27	18.28	72.55	8.08
Association rule 2	9.22	6.31	1.63	141.57	18.84	73.09	7.99
Association rule 3	9.30	6.50	1.68	123.46	17.27	64.67	7.75
Association rule 4	11.78	7.53	3.48	166.71	23.02	90.11	10.10
**H1-hESC**							
Promoter	8.45	3.47	2.49	73.33	14.12	24.35	4.84
Association rule 1	9.54	4.82	1.97	113.37	21.86	37.66	6.30
Association rule 2	10.76	5.82	1.69	123.45	21.27	38.26	5.35
Association rule 3	8.00	4.84	1.43	101.12	18.00	33.59	5.62
Association rule 4	10.56	5.37	3.27	141.38	35.12	46.16	6.73
**iPSC**							
Promoter	8.64	2.05	1.58	67.32	22.75	12.36	6.42
Association rule 1	10.25	5.64	1.66	107.50	18.35	26.77	8.59
Association rule 2	11.70	6.20	1.01	101.76	17.00	28.13	8.16
Association rule 3	11.69	6.89	1.01	90.85	16.60	24.89	7.76
Association rule 4	11.39	7.21	2.53	136.46	23.60	31.49	9.27

Z-Score: −2 −1 0 1 2

Table. S3 Normalized number of functional enrichments of putative transcriptional target genes using CTCF binding sites. * Wilcoxon signed-rank test $p < 0.05$

Table. S3 Normalized number of functional enrichments of putative transcriptional target genes using CTCF binding sites. * Wilcoxon signed-rank test $p < 0.05$

Z-Score: −2 −1 0 1 2

	KEGG	CTD Ontology	GO Slim	GO	Pathway Commons	Domains	Wiki Pathways
**Monocytes**							
Association rule 4	11.47	8.22	2.46	164.18	71.42	47.85	12.78
CTCF (FR+RF+FF+RR)	13.19	10.39	2.74	134.26	34.37	44.46	12.96
CTCF (FR)	42.92	19.53	5.66	509.86	196.58	112.14	35.11
**CD4⁺ T cells**							
Association rule 4	16.40	7.86	4.03	177.55	108.08	53.86	16.72
CTCF (FR+RF+FF+RR)	26.33	8.73	5.11	206.05	130.71	57.53	23.56
CTCF (FR)	69.39	14.66	24.91	560.44	220.54	133.26	46.54
**CD20⁺ B cells**							
Association rule 4	9.95	6.62	3.07	134.46	90.99	41.30	10.67
CTCF (FR+RF+FF+RR)	8.78	4.22	2.78	94.13	27.70	28.55	7.08
CTCF (FR)	28.86	9.72	6.61	304.01	220.77	99.89	22.68
**HUVEC**							
Association rule 4	13.46	8.99	5.08	191.73	59.10	82.65	10.13
CTCF (FR+RF+FF+RR)	19.35	10.59	3.85	155.11	28.36	57.87	13.58
CTCF (FR)	33.71	14.82	16.33	454.47	61.85	163.38	29.38
**IMR90**							
Association rule 4	10.55	7.68	3.76	142.10	30.05	74.86	8.78
CTCF (FR+RF+FF+RR)	13.88	17.55	9.22	252.24	39.92	123.18	22.45
CTCF (FR)	30.22	15.89	11.34	402.40	53.56	180.71	37.42
**MCF-7**							
Association rule 4	10.80	7.38	2.77	149.64	31.20	62.40	11.07
CTCF (FR+RF+FF+RR)	11.39	8.30	4.92	234.48	30.40	126.70	13.99
CTCF (FR)	22.34	11.48	6.09	397.69	51.39	145.43	37.58
**HMEC**							
Association rule 4	11.78	7.53	3.48	166.71	23.02	90.11	10.10
CTCF (FR+RF+FF+RR)	19.68	13.54	4.29	232.47	27.97	106.24	9.84
CTCF (FR)	29.59	14.46	6.97	390.45	43.90	124.81	30.78
**H1-hESC**							
Association rule 4	10.56	5.37	3.27	141.38	35.12	46.16	6.73
CTCF (FR+RF+FF+RR)	12.95	5.37	2.45	131.46	29.57	44.15	11.08
CTCF (FR)	28.21	10.55	9.02	303.41	56.95	139.44	19.72
**iPSC**							
Association rule 4	11.39	7.21	2.53	136.46	23.60	31.49	9.27
CTCF (FR+RF+FF+RR)	17.17	2.33	1.69	120.87	25.60	21.06	6.42
CTCF (FR)	36.94	6.09	6.86	274.30	31.37	83.40	16.80

Table S4. Normalized number of functional enrichments of transcriptional target genes predicted using promoters in monocytes, CD4$^+$ T cells, CD20$^+$ B cells, HUVEC, IMR90, MCF-7, HMEC, H1-hESC, and iPSC. Percent shows the ratio of putative transcriptional target genes that were replaced with randomly selected genes or randomly selected genes were added to. * Wilcoxon signed-rank test $p < 0.01$, ** $p < 0.05$

Table S4. Normalized number of functional enrichments of transcriptional target genes predicted using promoters in monocytes. Percent shows the ratio of putative transcriptional target genes that were replaced with randomly selected genes or randomly selected genes were added to. * Wilcoxon signed-rank test $p < 0.01$

−2 −1 0 1 2
Z-Score

Monocyte	KEGG	TF Targets	CTD Ontology	GO Slim	GO	Pathway Commons	BioMarkers	MicroRNA	Domains	WikiPathways	
60% replace	4.05	1.13	4.12	0.92	46.32	9.79	0.85	7.13	18.69	3.83	
40% replace	4.85	1.50	4.05	1.02	53.35	11.10	0.63	8.02	21.16	4.27	
20% replace	6.30	2.16	4.36	1.91	60.28	16.58	0.70	7.93	24.09	4.34	
10% replace	6.71	2.33	4.68	2.57	63.46	19.12	1.04	9.91	25.23	4.94	
5% replace	8.19	2.30	4.92	2.79	67.53	18.59	0.92	10.57	28.00	5.43	*
Native	8.46	2.59	5.07	2.76	70.34	24.36	1.02	10.93	29.13	5.87	*
5% add	7.76	2.29	5.03	2.64	65.52	24.10	0.90	10.29	26.97	5.44	
10% add	6.85	2.23	4.71	2.19	62.49	21.76	0.73	8.54	26.42	5.04	
20% add	6.93	1.78	4.03	1.40	53.91	17.23	0.53	7.43	23.61	4.54	
40% add	5.83	1.37	3.64	1.04	45.24	10.10	0.54	5.26	17.51	3.77	
60% add	4.43	1.21	3.66	0.74	39.92	8.86	0.44	4.25	17.62	3.92	

Normalized number of unique functional enrichements of transcriptional target genes predicted using promoters in monocytes.

Monocyte	KEGG	TF Targets	CTD Ontology	GO Slim	GO	Pathway Commons	BioMarkers	MicroRNA	Domains	WikiPathways
60% replace	2.38	0.61	3.42	0.48	30.52	6.40	0.58	4.07	11.73	2.47
40% replace	2.38	0.51	3.08	0.46	32.26	6.30	0.48	3.95	12.43	2.38
20% replace	2.69	0.56	3.08	0.63	30.61	8.31	0.39	3.81	11.61	2.23
10% replace	2.35	0.56	3.08	0.68	30.30	7.97	0.61	4.44	10.59	1.89
5% replace	2.52	0.56	3.10	0.63	30.06	7.78	0.44	4.65	10.74	2.18
Native	2.52	0.56	2.84	0.61	29.74	7.78	0.48	4.80	10.16	2.04
5% add	2.36	0.51	2.85	0.60	28.43	7.30	0.42	4.63	10.06	1.97
10% add	2.23	0.49	2.92	0.55	28.15	7.65	0.38	4.00	10.17	1.92
20% add	2.19	0.47	2.53	0.43	25.56	6.66	0.34	3.60	9.68	1.84
40% add	2.01	0.36	2.38	0.43	22.75	5.08	0.42	2.57	8.02	1.72
60% add	1.62	0.35	2.35	0.33	20.99	4.77	0.32	2.13	8.14	1.79

Number of functional enrichments of transcriptional target genes predicted using promoters in monocytes.

Monocyte	KEGG	TF Targets	CTD Ontology	GO Slim	GO	Pathway Commons	BioMarkers	MicroRNA	Domains	WikiPathways
60% replace	167	54	170	38	1,911	404	35	294	771	158
40% replace	200	62	167	42	2,201	458	26	331	873	176
20% replace	260	89	180	79	2,487	684	29	327	994	179
10% replace	277	96	193	106	2,618	789	43	409	1,041	204
5% replace	338	95	203	115	2,786	767	38	436	1,155	224
Native	349	107	209	114	2,902	1,005	42	451	1,202	242
5% add	335	99	217	114	2,828	1,040	39	444	1,164	235
10% add	310	101	213	99	2,826	984	33	386	1,195	228
20% add	342	88	199	69	2,662	851	26	367	1,166	224
40% add	336	79	210	60	2,607	582	31	303	1,009	217
60% add	292	80	241	49	2,630	584	29	280	1,161	258

Number of unique functional enrichments of transcriptional target genes predicted using promoters in monocytes.

Monocyte	KEGG	TF Targets	CTD Ontology	GO Slim	GO	Pathway Commons	BioMarkers	MicroRNA	Domains	WikiPathways
60% replace	98	25	141	20	1,259	264	24	168	484	102
40% replace	98	21	127	19	1,331	260	20	163	513	98
20% replace	111	23	127	26	1,263	343	16	157	479	92
10% replace	97	23	127	28	1,250	329	25	183	437	78
5% replace	104	23	128	26	1,240	321	18	192	443	90
Native	104	23	117	25	1,227	321	20	198	419	84
5% add	102	22	123	26	1,227	315	18	200	434	85
10% add	101	22	132	25	1,273	346	17	181	460	87
20% add	108	23	125	21	1,262	329	17	178	478	91
40% add	116	21	137	25	1,311	293	24	148	462	99
60% add	107	23	155	22	1,383	314	21	140	536	118

Normalized number of functional enrichments of transcriptional target genes predicted using promoters in CD4$^+$ T cells.
*** Wilcoxon signed-rank test $p < 0.01$, ** $p < 0.05$**

CD4$^+$ T cell	KEGG	TF Targets	CTD Ontology	GO Slim	GO	Pathway Commons	BioMarkers	MicroRNA	Domains	WikiPathways	
60% replace	3.34	0.87	3.53	0.68	46.81	10.99	0.53	6.15	18.63	3.63	
40% replace	3.71	1.35	3.54	0.79	51.74	12.10	1.02	7.12	17.22	4.78	
20% replace	4.61	1.65	4.41	1.07	59.16	18.18	0.57	7.38	20.68	5.85	
10% replace	5.03	2.02	4.18	0.95	63.38	22.95	0.68	9.29	21.95	5.85	
5% replace	4.98	2.19	4.44	1.04	66.46	27.52	0.80	10.66	22.75	6.73	*
Native	5.30	2.26	4.64	1.29	68.11	30.17	0.79	12.60	23.41	6.77	**
5% add	5.04	2.19	4.83	1.12	64.01	27.91	0.77	11.01	22.63	6.10	
10% add	4.71	2.06	4.16	1.00	60.12	25.81	0.69	9.58	21.50	5.42	
20% add	3.98	1.73	3.78	0.73	52.91	20.44	0.63	7.07	19.71	4.81	
40% add	3.57	1.29	3.30	0.57	45.90	15.81	0.62	4.41	16.20	4.49	
60% add	3.07	1.08	3.21	0.32	39.00	13.43	0.49	3.03	14.71	3.08	

Normalized number of unique functional enrichements of transcriptional target genes predicted using promoters in CD4$^+$ T cells.

CD4$^+$ T cell	KEGG	TF Targets	CTD Ontology	GO Slim	GO	Pathway Commons	BioMarkers	MicroRNA	Domains	WikiPathways
60% replace	1.89	0.42	2.76	0.45	29.04	6.35	0.38	3.06	12.08	1.94
40% replace	1.87	0.45	2.52	0.38	29.94	6.68	0.70	3.41	10.68	2.47
20% replace	1.90	0.43	2.77	0.45	29.04	7.68	0.35	3.32	10.91	2.27
10% replace	1.79	0.40	2.42	0.37	29.10	7.05	0.43	3.68	10.44	2.22
5% replace	1.74	0.42	2.49	0.38	28.35	7.15	0.47	3.83	10.06	2.19
Native	1.75	0.43	2.44	0.38	27.63	6.93	0.40	3.74	9.77	2.22
5% add	1.68	0.43	2.55	0.35	26.65	6.73	0.38	3.56	9.64	2.07
10% add	1.58	0.44	2.34	0.32	26.14	6.61	0.37	3.30	9.32	1.95
20% add	1.46	0.39	2.33	0.25	24.69	6.12	0.33	2.87	9.25	1.90
40% add	1.35	0.29	2.13	0.27	22.21	6.07	0.39	1.91	8.07	1.83
60% add	1.23	0.28	2.06	0.15	19.74	5.10	0.33	1.61	7.87	1.48

Number of functional enrichments of transcriptional target genes predicted using promoters in CD4$^+$ T cells.

CD4$^+$ T cell	KEGG	TF Targets	CTD Ontology	GO Slim	GO	Pathway Commons	BioMarkers	MicroRNA	Domains	WikiPathways
60% replace	200	52	211	41	2,802	658	32	368	1,115	217
40% replace	222	81	212	47	3,097	724	61	426	1,031	286
20% replace	276	99	264	64	3,541	1,088	34	442	1,238	350
10% replace	301	121	250	57	3,794	1,374	41	556	1,314	350
5% replace	298	131	266	62	3,978	1,647	48	638	1,362	403
Native	317	135	278	77	4,077	1,806	47	754	1,401	405
5% add	316	137	303	70	4,012	1,749	48	690	1,418	382
10% add	309	135	273	66	3,948	1,695	45	629	1,412	356
20% add	285	124	271	52	3,793	1,465	45	507	1,413	345
40% add	299	108	276	48	3,840	1,323	52	369	1,355	376
60% add	294	103	307	31	3,729	1,284	47	290	1,407	295

Number of unique functional enrichments of transcriptional target genes predicted using promoters in CD4$^+$ T cells.

CD4$^+$ T cell	KEGG	TF Targets	CTD Ontology	GO Slim	GO	Pathway Commons	BioMarkers	MicroRNA	Domains	WikiPathways
60% replace	113	25	165	27	1,738	380	23	183	723	116
40% replace	112	27	151	23	1,792	400	42	204	639	148
20% replace	114	26	166	27	1,738	460	21	199	653	136
10% replace	107	24	145	22	1,742	422	26	220	625	133
5% replace	104	25	149	23	1,697	428	28	229	602	131
Native	105	26	146	23	1,654	415	24	224	585	133
5% add	105	27	160	22	1,670	422	24	223	604	130
10% add	104	29	154	21	1,717	434	24	217	612	128
20% add	105	28	167	18	1,770	439	24	206	663	136
40% add	113	24	178	23	1,858	508	33	160	675	153
60% add	118	27	197	14	1,888	488	32	154	753	142

Normalized number of functional enrichments of transcriptional target genes predicted using promoters in CD20^{+} B cells.
*** Wilcoxon signed-rank test $p < 0.01$**

CD20^{+} B cell	KEGG	TF Targets	CTD Ontology	GO Slim	GO	Pathway Commons	BioMarkers	MicroRNA	Domains	WikiPathways	
60% replace	3.96	0.74	3.41	1.11	38.56	9.65	0.70	6.24	14.28	3.59	
40% replace	4.13	1.09	3.59	1.11	44.37	8.24	0.52	7.22	17.26	3.74	
20% replace	6.02	1.78	3.65	1.04	50.43	13.85	0.59	7.85	15.96	4.59	
10% replace	6.37	1.80	4.20	1.70	54.71	14.61	0.76	10.87	19.98	5.11	
5% replace	7.00	2.09	3.59	1.65	57.89	17.30	0.87	15.24	19.54	6.13	*
Native	7.02	2.24	3.70	1.91	60.39	17.85	0.85	20.61	20.65	6.26	*
5% add	6.56	1.95	3.59	1.72	56.44	17.03	0.75	16.74	19.00	5.90	
10% add	6.18	1.80	3.61	1.49	52.31	14.35	0.75	12.59	18.35	5.63	
20% add	6.03	1.42	3.12	1.25	47.64	12.54	0.58	9.19	15.21	5.25	
40% add	5.01	0.98	3.61	1.14	38.33	10.22	0.61	5.34	13.21	4.20	
60% add	4.35	0.93	2.63	0.72	33.18	9.01	0.38	3.70	13.00	3.21	

Normalized number of unique functional enrichements of transcriptional target genes predicted using promoters in CD20^{+} B cells.

CD20^{+} B cell	KEGG	TF Targets	CTD Ontology	GO Slim	GO	Pathway Commons	BioMarkers	MicroRNA	Domains	WikiPathways
60% replace	2.37	0.41	2.67	0.50	25.26	6.48	0.46	3.70	9.50	1.96
40% replace	2.04	0.39	2.63	0.57	27.11	5.09	0.37	3.83	9.96	2.02
20% replace	2.22	0.48	2.46	0.50	26.78	6.59	0.37	3.83	8.46	2.13
10% replace	2.11	0.50	2.48	0.59	25.98	6.98	0.37	4.22	9.09	2.15
5% replace	2.13	0.54	2.17	0.48	25.89	7.15	0.37	4.54	8.50	2.30
Native	2.02	0.50	2.09	0.50	25.91	7.15	0.35	5.02	8.63	2.30
5% add	1.93	0.44	2.08	0.48	24.79	7.37	0.33	4.57	8.58	2.10
10% add	1.84	0.44	2.08	0.40	23.94	6.48	0.36	4.08	8.46	2.16
20% add	1.91	0.31	1.91	0.40	23.02	5.75	0.25	3.85	7.64	1.96
40% add	1.65	0.26	2.16	0.40	19.94	5.02	0.30	2.66	7.36	1.84
60% add	1.54	0.30	1.67	0.24	18.06	4.30	0.23	2.01	6.64	1.47

Number of functional enrichments of transcriptional target genes predicted using promoters in CD20^{+} B cells.

CD20^{+} B cell	KEGG	TF Targets	CTD Ontology	GO Slim	GO	Pathway Commons	BioMarkers	MicroRNA	Domains	WikiPathways
60% replace	182	34	157	51	1,774	44	32	287	657	165
40% replace	190	50	165	51	2,041	379	24	332	794	172
20% replace	277	82	168	48	2,320	637	27	361	734	211
10% replace	293	83	193	78	2,517	672	35	500	919	235
5% replace	322	96	165	76	2,663	796	40	701	899	282
Native	323	103	170	88	2,778	821	39	948	950	288
5% add	316	94	173	83	2,718	820	36	806	915	284
10% add	312	91	182	75	2,639	724	38	635	926	284
20% add	332	78	172	69	2,624	691	32	506	838	289
40% add	322	63	232	73	2,464	657	39	343	849	270
60% add	320	68	193	53	2,438	662	28	272	955	236

Number of unique functional enrichments of transcriptional target genes predicted using promoters in CD20^{+} B cells.

CD20^{+} B cell	KEGG	TF Targets	CTD Ontology	GO Slim	GO	Pathway Commons	BioMarkers	MicroRNA	Domains	WikiPathways
60% replace	109	19	123	23	1,162	298	21	170	437	90
40% replace	94	18	121	26	1,247	234	17	176	458	93
20% replace	102	22	113	23	1,232	303	17	176	389	98
10% replace	97	23	114	27	1,195	321	17	194	418	99
5% replace	98	25	100	22	1,191	329	17	209	391	106
Native	93	23	96	23	1,192	329	16	231	397	106
5% add	93	21	100	23	1,194	355	16	220	413	101
10% add	93	22	105	20	1,208	327	18	206	427	109
20% add	105	17	105	22	1,268	317	14	212	421	108
40% add	106	17	139	26	1,282	323	19	171	473	118
60% add	113	22	123	18	1,327	316	17	148	488	108

Normalized number of functional enrichments of transcriptional target genes predicted using promoters in HUVEC.
*** Wilcoxon signed-rank test $p < 0.01$**

HUVEC	KEGG	TF Targets	CTD Ontology	GO Slim	GO	Pathway Commons	BioMarkers	MicroRNA	Domains	WikiPathways
60% replace	4.02	1.02	4.34	0.70	49.65	15.95	0.57	8.52	16.24	4.15
40% replace	4.79	1.63	5.26	1.15	63.62	14.90	0.93	9.03	16.34	4.31
20% replace	6.48	1.88	5.58	1.24	71.00	18.79	0.99	8.93	21.35	4.88
10% replace	6.67	1.95	5.20	1.37	72.08	17.45	1.02	9.51	20.33	4.37
5% replace	7.02	2.27	5.65	1.31	77.47	21.00	1.21	10.66	21.89	4.28
Native	7.50	2.43	5.74	1.34	79.71	22.21	1.21	10.75	22.40	4.59
5% add	6.71	2.07	5.46	1.28	75.96	20.42	1.28	10.21	20.79	4.12
10% add	6.20	1.95	5.76	1.28	70.92	18.30	1.08	9.19	21.35	3.93
20% add	6.37	1.68	5.09	1.15	67.04	17.11	1.04	7.59	18.41	4.64
40% add	4.98	1.37	4.84	1.10	56.15	13.34	0.80	6.21	17.77	3.90
60% add	4.49	1.08	4.43	0.62	50.24	12.32	0.90	4.69	14.80	3.64

Normalized number of unique functional enrichements of transcriptional target genes predicted using promoters in HUVEC.

HUVEC	KEGG	TF Targets	CTD Ontology	GO Slim	GO	Pathway Commons	BioMarkers	MicroRNA	Domains	WikiPathways
60% replace	2.65	0.70	3.45	0.41	36.28	10.05	0.48	5.23	11.61	2.74
40% replace	2.74	0.67	4.24	0.54	38.42	9.03	0.57	4.91	11.42	2.81
20% replace	3.10	0.80	4.02	0.70	37.24	9.54	0.67	5.04	12.41	2.58
10% replace	3.03	0.61	3.67	0.57	35.58	8.26	0.67	5.14	10.02	2.49
5% replace	2.87	0.64	3.73	0.51	35.80	10.21	0.73	5.42	10.43	2.30
Native	2.87	0.70	3.67	0.51	35.61	10.47	0.77	5.46	9.67	2.52
5% add	2.74	0.64	3.54	0.55	35.02	9.94	0.79	5.12	9.33	2.44
10% add	2.59	0.61	3.90	0.49	33.22	8.20	0.67	4.71	9.66	2.21
20% add	2.61	0.56	3.65	0.51	33.28	7.97	0.67	4.05	10.15	2.37
40% add	2.28	0.53	3.31	0.43	29.27	6.78	0.62	3.49	10.12	2.12
60% add	2.14	0.34	2.94	0.26	27.45	6.51	0.66	2.64	8.25	2.08

Number of functional enrichments of transcriptional target genes predicted using promoters in HUVEC.

HUVEC	KEGG	TF Targets	CTD Ontology	GO Slim	GO	Pathway Commons	BioMarkers	MicroRNA	Domains	WikiPathways
60% replace	126	32	136	22	1,556	500	18	267	509	130
40% replace	150	51	165	36	1,994	467	29	283	512	135
20% replace	203	59	175	39	2,225	589	31	280	669	153
10% replace	209	61	163	43	2,259	547	32	298	637	137
5% replace	220	71	177	41	2,428	658	38	334	686	134
Native	235	76	180	42	2,498	696	38	337	702	144
5% add	220	68	179	42	2,492	670	42	335	682	135
10% add	213	67	198	44	2,438	629	37	316	734	135
20% add	239	63	191	43	2,516	642	39	285	691	174
40% add	218	60	212	48	2,459	584	35	272	778	171
60% add	225	54	222	31	2,515	617	45	235	741	182

Number of unique functional enrichments of transcriptional target genes predicted using promoters in HUVEC.

HUVEC	KEGG	TF Targets	CTD Ontology	GO Slim	GO	Pathway Commons	BioMarkers	MicroRNA	Domains	WikiPathways
60% replace	83	22	108	13	1,137	315	15	164	364	86
40% replace	86	21	133	17	1,204	283	18	154	358	88
20% replace	97	25	126	22	1,167	299	21	158	389	81
10% replace	95	19	115	18	1,115	259	21	161	314	78
5% replace	90	20	117	16	1,122	320	23	170	327	72
Native	90	22	115	16	1,116	328	24	171	303	79
5% add	90	21	116	18	1,149	326	26	168	306	80
10% add	89	21	134	17	1,142	282	23	162	332	76
20% add	98	21	137	19	1,249	299	25	152	381	89
40% add	100	23	145	19	1,282	297	27	153	443	93
60% add	107	17	147	13	1,374	326	33	132	413	104

Normalized number of functional enrichments of transcriptional target genes predicted using promoters in IMR90.
*** Wilcoxon signed-rank test $p < 0.01$**

IMR90	KEGG	TF Targets	CTD Ontology	GO Slim	GO	Pathway Commons	BioMarkers	MicroRNA	Domains	WikiPathways	
60% replace	4.60	1.11	5.36	1.37	52.78	8.20	0.56	10.32	20.34	3.69	
40% replace	4.81	1.72	3.95	1.87	57.69	8.05	0.10	12.80	19.53	5.26	
20% replace	6.43	2.28	4.30	2.02	63.51	15.48	0.10	11.79	20.70	5.72	
10% replace	6.07	2.93	4.35	1.92	65.83	13.97	0.20	12.90	25.00	5.36	*
5% replace	7.03	3.54	5.36	2.28	66.59	12.60	0.15	13.26	24.29	4.86	
Native	6.63	3.74	5.21	2.43	68.31	11.64	0.15	13.31	25.50	5.47	
5% add	6.29	3.53	5.18	2.13	65.33	11.18	0.15	12.24	24.54	5.23	*
10% add	5.77	2.68	5.22	2.45	64.58	11.55	0.28	10.35	22.40	5.08	
20% add	5.54	2.28	4.86	1.90	58.53	9.30	0.17	10.62	21.48	5.16	
40% add	5.07	1.96	3.84	1.41	53.34	10.29	0.40	7.10	20.33	4.20	
60% add	5.20	1.39	3.96	1.14	51.13	10.27	0.48	5.80	19.40	4.37	

Normalized number of unique functional enrichements of transcriptional target genes predicted using promoters in IMR90.

IMR90	KEGG	TF Targets	CTD Ontology	GO Slim	GO	Pathway Commons	BioMarkers	MicroRNA	Domains	WikiPathways
60% replace	3.29	0.71	4.76	0.91	40.79	6.58	0.46	7.34	15.59	2.88
40% replace	3.39	0.76	3.34	1.06	41.55	6.58	0.10	7.74	14.02	3.64
20% replace	3.69	0.81	3.34	1.11	40.48	11.28	0.10	7.03	14.07	3.54
10% replace	3.34	0.91	3.44	1.11	39.57	8.60	0.20	7.29	14.78	3.14
5% replace	3.34	0.96	3.64	1.06	38.31	8.30	0.15	7.34	13.92	2.63
Native	3.29	0.86	3.74	1.06	38.00	6.88	0.15	7.24	13.66	2.68
5% add	3.10	0.82	3.73	1.02	36.78	7.02	0.15	6.87	13.84	2.66
10% add	2.82	0.74	3.65	1.06	37.56	6.84	0.23	5.77	12.61	2.77
20% add	2.66	0.72	3.30	0.85	35.95	5.88	0.17	6.05	12.56	2.83
40% add	2.50	0.80	2.90	0.69	34.39	6.34	0.36	4.71	11.89	2.32
60% add	2.57	0.51	2.98	0.70	33.44	7.07	0.38	3.74	11.86	2.63

Number of functional enrichments of transcriptional target genes predicted using promoters in IMR90.

IMR90	KEGG	TF Targets	CTD Ontology	GO Slim	GO	Pathway Commons	BioMarkers	MicroRNA	Domains	WikiPathways
60% replace	91	22	106	27	1,043	162	11	204	402	73
40% replace	95	34	78	37	1,140	159	2	253	386	104
20% replace	127	45	85	40	1,255	306	2	233	409	113
10% replace	120	58	86	38	1,301	276	4	255	494	106
5% replace	139	70	106	45	1,316	249	3	262	480	96
Native	131	74	103	48	1,350	230	3	263	504	108
5% add	130	73	107	44	1,350	231	3	253	507	108
10% add	125	58	113	53	1,398	250	6	224	485	110
20% add	131	54	115	45	1,384	220	4	251	508	122
40% add	140	54	106	39	1,472	284	11	196	561	116
60% add	164	44	125	36	1,613	324	15	183	612	138

Number of unique functional enrichments of transcriptional target genes predicted using promoters in IMR90.

IMR90	KEGG	TF Targets	CTD Ontology	GO Slim	GO	Pathway Commons	BioMarkers	MicroRNA	Domains	WikiPathways
60% replace	65	14	94	18	806	130	9	145	308	57
40% replace	67	15	66	21	821	130	2	153	277	72
20% replace	73	16	66	22	800	223	2	139	278	70
10% replace	66	18	68	22	782	170	4	144	292	62
5% replace	66	19	72	21	757	164	3	145	275	52
Native	65	17	74	21	751	136	3	143	270	53
5% add	64	17	77	21	760	145	3	142	286	55
10% add	61	16	79	23	813	148	5	125	273	60
20% add	63	17	78	20	850	139	4	143	297	67
40% add	69	22	80	19	949	175	10	130	328	64
60% add	81	16	94	22	1,055	223	12	118	374	83

Normalized number of functional enrichments of transcriptional target genes predicted using promoters in MCF-7.
*** Wilcoxon signed-rank test *p* < 0.01**

MCF-7	KEGG	TF Targets	CTD Ontology	GO Slim	GO	Pathway Commons	BioMarkers	MicroRNA	Domains	WikiPathways	
60% replace	3.69	1.54	3.13	1.23	56.81	15.84	0.31	10.92	18.46	4.20	
40% replace	5.90	1.90	3.95	1.74	64.39	15.07	0.56	12.05	18.15	6.41	
20% replace	5.95	2.67	4.20	1.49	68.70	15.59	0.62	12.71	22.46	6.87	
10% replace	6.15	3.64	4.51	2.05	71.26	20.00	1.18	14.66	22.66	8.15	
5% replace	6.51	3.13	5.13	1.59	76.75	21.84	1.13	14.46	24.92	8.10	*
Native	6.97	3.74	5.43	1.69	77.88	24.71	1.03	14.77	25.99	8.77	
5% add	6.28	3.24	5.15	1.62	73.75	22.02	0.98	13.73	23.78	8.14	*
10% add	6.46	3.09	4.54	1.54	70.11	21.58	0.94	13.10	21.48	7.77	
20% add	6.86	2.79	4.50	1.24	68.31	16.63	1.07	9.73	20.48	8.14	
40% add	6.28	2.13	3.97	1.43	60.88	16.71	0.88	7.75	18.84	6.94	
60% add	4.18	1.54	4.05	0.39	51.29	10.76	0.84	6.33	17.18	5.46	

Normalized number of unique functional enrichements of transcriptional target genes predicted using promoters in MCF-7.

MCF-7	KEGG	TF Targets	CTD Ontology	GO Slim	GO	Pathway Commons	BioMarkers	MicroRNA	Domains	WikiPathways
60% replace	2.82	0.97	2.97	0.72	43.07	10.77	0.26	7.49	13.69	2.87
40% replace	3.64	0.77	3.13	1.08	43.53	10.15	0.46	7.23	13.54	3.49
20% replace	3.18	0.82	3.02	0.82	40.96	9.90	0.31	7.64	12.71	3.95
10% replace	2.87	1.08	2.72	1.03	39.53	10.61	0.41	8.20	13.07	4.10
5% replace	2.92	0.82	3.23	0.72	40.86	11.23	0.26	7.90	13.33	3.28
Native	2.92	0.87	3.02	0.77	39.68	11.43	0.21	8.05	12.97	3.59
5% add	2.80	0.88	3.09	0.74	38.30	10.84	0.20	7.70	13.24	3.48
10% add	2.95	0.70	2.71	0.70	37.30	11.09	0.28	7.49	11.98	3.32
20% add	3.13	0.94	2.70	0.69	37.54	9.08	0.47	5.57	12.30	3.30
40% add	3.08	0.73	2.42	0.77	35.25	9.95	0.33	4.55	11.09	3.08
60% add	2.34	0.61	2.89	0.35	30.93	6.71	0.35	4.11	11.59	2.54

Number of functional enrichments of transcriptional target genes predicted using promoters in MCF-7.

MCF-7	KEGG	TF Targets	CTD Ontology	GO Slim	GO	Pathway Commons	BioMarkers	MicroRNA	Domains	WikiPathways
60% replace	72	30	61	24	1,108	309	6	213	360	82
40% replace	115	37	77	34	1,256	294	11	235	354	125
20% replace	116	52	82	29	1,340	304	12	248	438	134
10% replace	120	71	88	40	1,390	390	23	286	442	159
5% replace	127	61	100	31	1,497	426	22	282	486	158
Native	136	73	106	33	1,519	482	20	288	507	171
5% add	128	66	105	33	1,504	449	20	280	485	166
10% add	138	66	97	33	1,498	461	20	280	459	166
20% add	160	65	105	29	1,594	388	25	227	478	190
40% add	171	58	108	39	1,658	455	24	211	513	189
60% add	130	48	126	12	1,597	335	26	197	535	170

Number of unique functional enrichments of transcriptional target genes predicted using promoters in MCF-7.

MCF-7	KEGG	TF Targets	CTD Ontology	GO Slim	GO	Pathway Commons	BioMarkers	MicroRNA	Domains	WikiPathways
60% replace	55	19	58	14	840	210	5	146	267	56
40% replace	71	15	61	21	849	198	9	141	264	68
20% replace	62	16	59	16	799	193	6	149	248	77
10% replace	56	21	53	20	771	207	8	160	255	80
5% replace	57	16	63	14	797	219	5	154	260	64
Native	57	17	59	15	774	223	4	157	253	70
5% add	57	18	63	15	781	221	4	157	270	71
10% add	63	15	58	15	797	237	6	160	256	71
20% add	73	22	63	16	876	212	11	130	287	77
40% add	84	20	66	21	960	271	9	124	302	84
60% add	73	19	90	11	963	209	11	128	361	79

Normalized number of functional enrichments of transcriptional target genes predicted using promoters in HMEC.
*** Wilcoxon signed-rank test $p < 0.01$, ** $p < 0.05$**

HMEC	KEGG	TF Targets	CTD Ontology	GO Slim	GO	Pathway Commons	BioMarkers	MicroRNA	Domains	WikiPathways	
60% replace	4.58	1.53	5.17	0.90	53.67	12.54	0.63	9.53	19.10	4.63	
40% replace	4.81	2.20	4.94	1.30	58.48	13.44	0.40	9.48	22.07	4.81	
20% replace	6.56	2.74	5.39	2.07	58.88	11.19	0.63	10.25	25.35	4.36	
10% replace	6.97	2.79	4.85	1.35	62.30	12.23	0.40	11.91	24.18	7.01	
5% replace	8.45	2.79	4.99	1.44	64.77	13.71	0.63	11.15	24.72	6.29	**
Native	8.76	2.92	5.03	1.53	65.80	14.07	0.49	11.19	26.52	6.70	
5% add	8.08	2.54	4.30	1.29	63.26	13.84	0.47	10.70	24.71	6.36	*
10% add	7.55	2.58	4.88	0.98	58.61	13.53	0.62	10.38	23.75	5.58	
20% add	8.22	2.22	4.28	0.83	54.01	12.58	0.56	9.01	22.16	5.15	
40% add	5.99	2.03	4.54	1.09	51.11	10.62	0.77	6.79	20.50	4.67	
60% add	5.10	1.38	4.08	0.84	47.10	8.95	0.34	5.80	19.03	3.72	

Normalized number of unique functional enrichements of transcriptional target genes predicted using promoters in HMEC.

HMEC	KEGG	TF Targets	CTD Ontology	GO Slim	GO	Pathway Commons	BioMarkers	MicroRNA	Domains	WikiPathways
60% replace	3.19	0.90	4.00	0.63	40.14	9.39	0.58	6.47	14.34	3.69
40% replace	3.10	0.99	3.96	0.94	41.08	10.38	0.36	5.80	14.47	3.64
20% replace	3.55	1.03	3.55	0.99	37.67	7.51	0.45	5.62	16.14	2.97
10% replace	3.60	0.90	3.28	0.81	36.63	7.82	0.31	5.89	13.53	3.46
5% replace	3.96	0.76	3.19	0.72	35.87	8.90	0.31	5.44	14.02	3.28
Native	4.31	0.72	3.01	0.76	35.37	8.41	0.27	5.39	13.71	3.51
5% add	4.04	0.69	2.84	0.64	34.98	7.99	0.26	5.11	12.93	3.14
10% add	3.61	0.70	2.95	0.49	34.12	8.74	0.33	4.92	12.80	3.16
20% add	3.91	0.83	2.93	0.49	31.81	7.89	0.34	4.58	12.36	2.70
40% add	3.03	0.61	3.15	0.61	32.18	7.14	0.48	3.60	12.42	2.51
60% add	2.65	0.56	3.12	0.42	29.81	6.05	0.28	3.35	11.63	2.20

Number of functional enrichments of transcriptional target genes predicted using promoters in HMEC.

HMEC	KEGG	TF Targets	CTD Ontology	GO Slim	GO	Pathway Commons	BioMarkers	MicroRNA	Domains	WikiPathways
60% replace	102	34	115	20	1,194	279	14	212	425	103
40% replace	107	49	110	29	1,301	299	9	211	491	107
20% replace	146	61	120	46	1,310	249	14	228	564	97
10% replace	155	62	108	30	1,386	272	9	265	538	156
5% replace	188	62	111	32	1,441	305	14	248	550	140
Native	195	65	112	34	1,464	313	11	249	590	149
5% add	188	59	100	30	1,472	322	11	249	575	148
10% add	184	63	119	24	1,429	330	15	253	579	136
20% add	219	59	114	22	1,438	335	15	240	590	137
40% add	186	63	141	34	1,588	330	24	211	637	145
60% add	181	49	145	30	1,673	318	12	206	676	132

Number of unique functional enrichments of transcriptional target genes predicted using promoters in HMEC.

HMEC	KEGG	TF Targets	CTD Ontology	GO Slim	GO	Pathway Commons	BioMarkers	MicroRNA	Domains	WikiPathways
60% replace	71	20	89	14	893	209	13	144	319	82
40% replace	69	22	88	21	914	231	8	129	322	81
20% replace	79	23	79	22	838	167	10	125	359	66
10% replace	80	20	73	18	815	174	7	131	301	77
5% replace	88	17	71	16	798	198	7	121	312	73
Native	96	16	67	17	787	187	6	120	305	78
5% add	94	16	66	15	814	186	6	119	301	73
10% add	88	17	72	12	832	213	8	120	312	77
20% add	104	22	78	13	847	210	9	122	329	72
40% add	94	19	98	19	1,000	222	15	112	386	78
60% add	94	20	111	15	1,059	215	10	119	413	78

Normalized number of functional enrichments of transcriptional target genes predicted using promoters in H1-hESC.
*** Wilcoxon signed-rank test $p < 0.01$, ** p < 0.05**

H1−hESC	KEGG	TF Targets	CTD Ontology	GO Slim	GO	Pathway Commons	BioMarkers	MicroRNA	Domains	WikiPathways	
60% replace	3.61	0.90	3.18	0.61	46.60	9.43	0.33	7.91	14.85	3.21	
40% replace	4.48	1.52	3.79	1.66	51.26	12.64	0.83	7.44	14.95	3.61	
20% replace	6.90	1.95	4.01	1.66	60.32	13.26	0.69	9.25	19.54	4.44	
10% replace	8.02	2.10	3.07	2.02	66.03	13.91	0.72	8.71	22.03	4.80	
5% replace	7.69	1.88	3.50	2.24	69.71	13.69	0.72	9.21	22.54	5.02	
Native	8.45	2.10	3.47	2.49	73.33	14.12	0.79	9.36	24.35	4.84	**
5% add	8.39	1.93	3.24	2.28	69.99	15.32	0.72	8.83	23.64	5.56	*
10% add	7.64	1.94	3.13	2.37	65.55	12.85	0.59	7.81	22.10	4.61	
20% add	7.30	1.51	3.35	2.05	61.12	10.26	0.45	7.00	22.57	4.50	
40% add	5.82	1.50	2.79	1.01	51.75	9.05	0.59	4.73	19.93	3.77	
60% add	5.16	1.24	2.99	0.59	47.04	8.87	0.79	4.27	15.94	2.94	

Normalized number of unique functional enrichements of transcriptional target genes predicted using promoters in H1-hESC.

H1−hESC	KEGG	TF Targets	CTD Ontology	GO Slim	GO	Pathway Commons	BioMarkers	MicroRNA	Domains	WikiPathways
60% replace	2.28	0.54	2.64	0.47	33.38	7.40	0.25	5.20	11.23	2.46
40% replace	2.67	0.58	3.29	0.72	33.45	7.98	0.61	5.27	11.45	2.17
20% replace	3.25	0.58	3.00	0.54	34.17	7.91	0.61	5.67	11.49	2.56
10% replace	3.36	0.65	2.02	0.65	33.16	6.79	0.51	5.31	12.17	2.71
5% replace	3.21	0.51	2.56	0.72	33.30	7.55	0.61	5.24	11.09	2.71
Native	3.21	0.58	2.28	0.72	33.48	6.75	0.58	5.27	11.67	2.46
5% add	3.21	0.48	2.21	0.69	32.75	8.11	0.55	5.00	11.60	2.55
10% add	3.03	0.49	2.37	0.76	32.31	7.02	0.46	4.58	11.53	2.21
20% add	2.96	0.51	2.32	0.75	31.23	5.52	0.36	4.04	11.83	2.38
40% add	2.33	0.59	2.27	0.44	27.66	4.94	0.49	3.13	10.96	2.09
60% add	2.28	0.43	2.37	0.32	25.83	5.31	0.59	2.67	9.68	1.63

Number of functional enrichments of transcriptional target genes predicted using promoters in H1-hESC.

H1−hESC	KEGG	TF Targets	CTD Ontology	GO Slim	GO	Pathway Commons	BioMarkers	MicroRNA	Domains	WikiPathways
60% replace	100	25	88	17	1,290	261	9	219	411	89
40% replace	124	42	105	46	1,419	350	23	206	414	100
20% replace	191	54	111	46	1,670	367	19	256	541	123
10% replace	222	58	85	56	1,828	385	20	241	610	133
5% replace	213	52	97	62	1,930	379	20	255	624	139
Native	234	58	96	69	2,030	391	22	259	674	134
5% add	243	56	94	66	2,028	444	21	256	685	161
10% add	232	59	95	72	1,990	390	18	237	671	140
20% add	242	50	111	68	2,026	340	15	232	748	149
40% add	225	58	108	39	2,002	350	23	183	771	146
60% add	228	55	132	26	2,080	392	35	189	705	130

Number of unique functional enrichments of transcriptional target genes predicted using promoters in H1-hESC.

H1−hESC	KEGG	TF Targets	CTD Ontology	GO Slim	GO	Pathway Commons	BioMarkers	MicroRNA	Domains	WikiPathways
60% replace	63	15	73	13	924	205	7	144	311	68
40% replace	74	16	91	20	926	221	17	146	317	60
20% replace	90	16	83	15	946	219	17	157	318	71
10% replace	93	18	56	18	918	188	14	147	337	75
5% replace	89	14	71	20	922	209	17	145	307	75
Native	89	16	63	20	927	187	16	146	323	68
5% add	93	14	64	20	949	235	16	145	336	74
10% add	92	15	72	23	981	213	14	139	350	67
20% add	98	17	77	25	1,035	183	12	134	392	79
40% add	90	23	88	17	1,070	191	19	121	424	81
60% add	101	19	105	14	1,142	235	26	118	428	72

Normalized number of functional enrichments of transcriptional target genes predicted using promoters in iPSC.
*** Wilcoxon signed-rank test $p < 0.01$, ** $p < 0.05$**

iPS	KEGG	TF Targets	CTD Ontology	GO Slim	GO	Pathway Commons	BioMarkers	MicroRNA	Domains	WikiPathways
60% replace	2.82	0.73	2.10	0.60	39.82	9.54	0.73	9.11	10.05	3.59
40% replace	5.65	1.28	1.84	1.33	47.05	12.15	0.68	9.11	10.78	4.58
20% replace	7.48	1.92	2.01	1.03	53.25	15.27	0.81	9.84	12.45	5.65
10% replace	8.25	1.54	1.50	1.20	61.55	17.71	0.73	9.97	11.63	6.20
5% replace	9.11	1.88	1.92	1.54	63.77	18.09	0.94	10.65	11.51	6.37 *
Native	8.64	1.80	2.05	1.58	67.32	22.75	0.81	11.63	12.36	6.42
5% add	7.77	1.72	2.21	1.31	62.05	19.33	0.86	10.55	11.85	5.97 **
10% add	7.10	1.56	2.03	1.25	57.82	15.84	0.74	8.58	12.95	4.92
20% add	6.22	1.89	1.93	0.93	52.38	14.54	0.82	6.54	11.65	4.57
40% add	5.27	1.26	2.11	0.61	43.44	11.91	0.77	6.12	10.81	4.07
60% add	3.94	1.34	1.87	0.51	38.70	9.00	0.51	4.66	10.18	3.56

Normalized number of unique functional enrichements of transcriptional target genes predicted using promoters in iPSC.

iPS	KEGG	TF Targets	CTD Ontology	GO Slim	GO	Pathway Commons	BioMarkers	MicroRNA	Domains	WikiPathways
60% replace	1.97	0.43	1.97	0.47	30.41	7.57	0.51	6.16	7.66	2.78
40% replace	3.12	0.68	1.58	0.77	32.93	7.01	0.56	5.65	7.96	2.61
20% replace	3.21	0.68	1.67	0.60	32.21	9.45	0.47	6.03	7.53	2.65
10% replace	3.04	0.56	1.33	0.60	32.93	10.26	0.43	5.60	7.06	2.74
5% replace	3.12	0.60	1.67	0.68	32.33	8.64	0.43	5.86	6.59	2.57
Native	2.95	0.64	1.63	0.68	31.48	8.17	0.34	5.69	6.89	2.57
5% add	2.74	0.65	1.72	0.57	29.80	8.09	0.41	5.48	6.54	2.37
10% add	2.61	0.62	1.72	0.55	29.10	8.23	0.35	4.92	7.49	2.18
20% add	2.32	0.57	1.54	0.39	27.97	6.65	0.43	3.93	6.97	2.18
40% add	2.39	0.49	1.71	0.43	24.77	6.89	0.34	3.98	6.80	2.14
60% add	1.98	0.46	1.69	0.29	22.63	5.28	0.32	2.95	6.75	2.09

Number of functional enrichments of transcriptional target genes predicted using promoters in iPSC.

iPS	KEGG	TF Targets	CTD Ontology	GO Slim	GO	Pathway Commons	BioMarkers	MicroRNA	Domains	WikiPathways
60% replace	66	17	49	14	931	223	17	213	235	84
40% replace	132	30	43	31	1,100	284	16	213	252	107
20% replace	175	45	47	24	1,245	357	19	230	291	132
10% replace	193	36	35	28	1,439	414	17	233	272	145
5% replace	213	44	45	36	1,491	423	22	249	269	149
Native	202	42	48	37	1,574	532	19	272	289	150
5% add	190	42	54	32	1,518	473	21	258	290	146
10% add	182	40	52	32	1,482	406	19	220	332	126
20% add	174	53	54	26	1,466	407	23	183	326	128
40% add	172	41	69	20	1,419	389	25	200	353	133
60% add	147	50	70	19	1,445	336	19	174	380	133

Number of unique functional enrichments of transcriptional target genes predicted using promoters in iPSC.

iPS	KEGG	TF Targets	CTD Ontology	GO Slim	GO	Pathway Commons	BioMarkers	MicroRNA	Domains	WikiPathways
60% replace	46	10	46	11	711	177	12	144	179	65
40% replace	73	16	37	18	770	164	13	132	186	61
20% replace	75	16	39	14	753	221	11	141	176	62
10% replace	71	13	31	14	770	240	10	131	165	64
5% replace	73	14	39	16	756	202	10	137	154	60
Native	69	15	38	16	736	191	8	133	161	60
5% add	67	16	42	14	729	198	10	134	160	58
10% add	67	16	44	14	746	211	9	126	192	56
20% add	65	16	43	11	783	186	12	110	195	61
40% add	78	16	56	14	809	225	11	130	222	70
60% add	74	17	63	11	845	197	12	110	252	78

Table S5. Normalized number of functional enrichments of transcriptional target genes predicted using promoter regions in H1-hESC (ChIP-seq data of transcription factors). Percent shows the ratio of putative transcriptional target genes that were replaced with randomly selected genes or randomly selected genes were added to. * Wilcoxon signed-rank test $p < 0.01$, ** $p < 0.05$

Table S5. Normalized number of functional enrichments of transcriptional target genes predicted using promoters in H1-hESC (ChIP-seq data of 19 transcription factors). Percent shows the ratio of putative transcriptional target genes that were replaced with randomly selected genes or randomly selected genes were added to. * Wilcoxon signed-rank test $p < 0.01$, ** $p < 0.05$

−2 −1 0 1 2
Z-Score

H1-hESC	KEGG	TF Targets	CTD Ontology	GO Slim	GO	Pathway Commons	BioMarkers	MicroRNA	Domains	WikiPathways	
60% replace	0.24	0.12	0.24	0.49	9.14	0.12	0.12	0.98	1.83	0.24	
40% replace	1.22	0.49	0.37	5.85	34.61	1.22	0.61	65.57	5.97	1.22	
20% replace	4.14	1.83	0.12	14.02	89.58	6.58	2.93	632.93	14.99	5.48	
10% replace	7.68	4.14	1.10	16.21	113.47	11.94	3.66	729.58	22.91	8.53	
5% replace	10.73	5.61	0.85	16.21	122.61	18.28	4.14	769.68	27.06	9.14	
Native	15.97	5.85	1.22	17.55	136.39	21.33	5.61	791.25	35.10	13.29	*
5% add	13.35	5.69	0.35	15.79	128.74	18.69	4.99	746.23	30.42	11.38	*
10% add	10.75	5.32	0.66	15.07	111.48	15.07	3.99	707.98	29.92	9.86	
20% add	8.02	4.37	0.20	13.10	97.31	9.95	2.84	635.25	21.84	5.79	
40% add	3.05	3.13	0.26	10.97	69.04	5.14	1.83	516.12	13.93	3.31	
60% add	1.98	2.13	0.15	7.85	49.21	3.50	0.61	356.67	9.45	2.29	

Normalized number of unique functional enrichements of transcriptional target genes predicted using promoters in H1-hESC.

H1-hESC	KEGG	TF Targets	CTD Ontology	GO Slim	GO	Pathway Commons	BioMarkers	MicroRNA	Domains	WikiPathways
60% replace	0.24	0.12	0.24	0.37	8.17	0.12	0.12	0.98	1.83	0.24
40% replace	1.10	0.49	0.37	1.34	15.97	1.22	0.61	23.40	4.27	1.10
20% replace	1.95	0.49	0.12	2.68	29.37	3.66	1.34	55.58	8.41	2.19
10% replace	2.68	0.73	0.98	3.29	34.00	5.24	1.10	58.26	10.12	2.32
5% replace	2.68	0.98	0.73	3.17	33.52	5.36	1.10	58.50	11.21	2.32
Native	3.29	0.85	0.85	2.80	33.76	4.39	1.22	59.23	11.70	3.29
5% add	2.79	0.81	0.35	3.02	32.51	4.53	1.39	56.19	11.49	2.67
10% add	2.33	0.89	0.66	2.44	28.70	3.77	0.89	52.97	9.64	2.33
20% add	1.93	0.71	0.20	2.23	26.31	2.34	0.81	48.45	8.63	1.63
40% add	1.22	0.61	0.26	2.09	20.02	1.65	0.70	40.31	5.66	0.96
60% add	0.91	0.53	0.15	1.60	15.39	1.52	0.38	33.44	4.80	0.99

Number of functional enrichments of transcriptional target genes predicted using promoters in H1-hESC.

H1-hESC	KEGG	TF Targets	CTD Ontology	GO Slim	GO	Pathway Commons	BioMarkers	MicroRNA	Domains	WikiPathways
60% replace	2	1	2	4	75	1	1	8	15	2
40% replace	10	4	3	48	284	10	5	538	49	10
20% replace	34	15	1	115	735	54	24	5,193	123	45
10% replace	63	34	9	133	931	98	30	5,986	188	70
5% replace	88	46	7	133	1,006	150	34	6,315	222	75
Native	131	48	10	144	1,119	175	46	6,492	288	109
5% add	115	49	3	136	1,109	161	43	6,428	262	98
10% add	97	48	6	136	1,006	136	36	6,389	270	89
20% add	79	43	2	129	958	98	28	6,254	215	57
40% add	35	36	3	126	793	59	21	5,928	160	38
60% add	26	28	2	103	646	46	8	4,682	124	30

Number of unique functional enrichments of transcriptional target genes predicted using promoters in H1-hESC.

H1-hESC	KEGG	TF Targets	CTD Ontology	GO Slim	GO	Pathway Commons	BioMarkers	MicroRNA	Domains	WikiPathways
60% replace	2	1	2	3	67	1	1	8	15	2
40% replace	9	4	3	11	131	10	5	192	35	9
20% replace	16	4	1	22	241	30	11	456	69	18
10% replace	22	6	8	27	279	43	9	478	83	19
5% replace	22	8	6	26	275	44	9	480	92	19
Native	27	7	7	23	277	36	10	486	96	27
5% add	24	7	3	26	280	39	12	484	99	23
10% add	21	8	6	22	259	34	8	478	87	21
20% add	19	7	2	22	259	23	8	477	85	16
40% add	14	7	3	24	230	19	8	463	65	11
60% add	12	7	2	21	202	20	5	439	63	13

Table S6. Functional enrichments of gene expression information alone (highly expressed genes) using Pathway Commons, independent of transcriptional target genes (*p*-value < 0.01).

Monocytes - Pathway Commons	No. in selected genes	No. in all RefSeq	*p*-value
Gene Expression	161	297	0.0.E+00
Formation and Maturation of mRNA Transcript	89	164	1.5.E-03
The citric acid (TCA) cycle and respiratory electron transport	59	101	0.0.E+00
Androgen-mediated signaling	54	106	1.0.E-03
HIV Life Cycle	51	91	7.0.E-03
Regulation of Androgen receptor activity	49	96	2.5.E-03
Late Phase of HIV Life Cycle	47	82	4.5.E-03
Respiratory electron transport, ATP synthesis by chemiosmotic coupling, and heat production by uncoupling proteins.	45	78	3.0.E-03
Respiratory electron transport	40	65	0.0.E+00
BCR signaling pathway	36	63	0.0.E+00
Signaling by Interleukins	36	70	9.0.E-03
Endogenous TLR signaling	33	56	0.0.E+00
Fc-epsilon receptor I signaling in mast cells	33	59	2.0.E-03
RNA Polymerase II Pre-transcription Events	31	52	9.5.E-03
Toll Receptor Cascades	31	55	3.5.E-03
tRNA Aminoacylation	26	41	0.0.E+00
Toll Like Receptor 4 (TLR4) Cascade	25	43	1.0.E-03
Activated TLR4 signalling	22	39	3.5.E-03
HIV-1 Nef: Negative effector of Fas and TNF-alpha	21	35	2.0.E-03
RIG-I/MDA5 mediated induction of IFN-alpha/beta pathways	21	35	1.0.E-03
Toll Like Receptor 2 (TLR2) Cascade	21	37	5.0.E-03
Toll Like Receptor TLR6:TLR2 Cascade	21	37	5.0.E-03
Toll Like Receptor TLR1:TLR2 Cascade	20	36	5.0.E-03
FoxO family signaling	19	29	0.0.E+00
EPO signaling pathway	19	31	4.5.E-03
Pyruvate metabolism and Citric Acid (TCA) cycle	18	27	0.0.E+00
Interleukin-1 signaling	18	29	5.0.E-04
Cytosolic tRNA aminoacylation	18	24	0.0.E+00
Vpr-mediated nuclear import of PICs	16	25	0.0.E+00
Osteopontin-mediated events	16	26	1.0.E-03
Interactions of Vpr with host cellular proteins	16	25	0.0.E+00
Protein folding	15	21	0.0.E+00
Citric acid cycle (TCA cycle)	13	19	0.0.E+00
Chaperonin-mediated protein folding	12	16	0.0.E+00
Cooperation of Prefoldin and TriC/CCT in actin and tubulin folding	12	15	0.0.E+00
Prefoldin mediated transfer of substrate to CCT/TriC	11	14	0.0.E+00
Extrinsic Pathway for Apoptosis	10	13	0.0.E+00
Death Receptor Signalling	10	13	0.0.E+00
GPVI-mediated activation cascade	9	15	4.0.E-03
S1P2 pathway	8	13	5.0.E-04
EPHA2 forward signaling	8	13	3.5.E-03
Metabolism of polyamines	8	13	0.0.E+00
Folding of actin by CCT/TriC	8	9	0.0.E+00
Association of TriC/CCT with target proteins during biosynthesis	7	9	1.0.E-03
heme biosynthesis II	7	10	0.0.E+00
isoleucine degradation I	7	11	4.5.E-03
Formation of tubulin folding intermediates by CCT/TriC	7	8	0.0.E+00
Caspase-8 is formed from procaspase-8	6	9	6.0.E-03
COPI Mediated Transport	6	9	9.0.E-03
NF-kB activation through FADD/RIP-1 pathway mediated by caspase-8 and -10	6	8	3.0.E-03
Golgi to ER Retrograde Transport	6	9	9.0.E-03
Activation of Pro-Caspase 8	6	9	6.0.E-03
IKK complex recruitment mediated by RIP1	6	7	5.0.E-04
Heme biosynthesis	5	6	1.0.E-03
Metabolism of porphyrins	5	7	2.0.E-03
TNF signaling	5	7	2.5.E-03
TRAF6 mediated NF-kB activation	5	7	7.0.E-03
Beta oxidation of lauroyl-CoA to decanoyl-CoA-CoA	4	5	2.0.E-03
Beta oxidation of hexanoyl-CoA to butanoyl-CoA	4	5	2.0.E-03
Beta oxidation of decanoyl-CoA to octanoyl-CoA-CoA	4	5	2.5.E-03
Beta oxidation of octanoyl-CoA to hexanoyl-CoA	4	5	2.5.E-03
GRB2:SOS provides linkage to MAPK signaling for Intergrins	4	5	2.0.E-03
G alpha (q) signalling events	4	4	1.0.E-03
Beta-oxidation of very long chain fatty acids	4	5	4.0.E-03
Interleukin receptor SHC signaling	4	4	5.5.E-03
AKT phosphorylates targets in the nucleus	3	3	8.5.E-03
DNA Damage Reversal	3	3	7.0.E-03

CD4$^+$ T cells - Pathway Commons	No. in selected genes	No. in all RefSeq	*p*-value
Gene Expression	271	297	0.0E+00
Metabolism of RNA	179	212	0.0E+00
HIV Infection	155	173	0.0E+00
Formation and Maturation of mRNA Transcript	154	164	0.0E+00
Metabolism of mRNA	149	176	0.0E+00
TNF alpha/NF-kB	132	159	0.0E+00
mRNA Processing	128	136	0.0E+00
Transcription	120	138	0.0E+00
Processing of Capped Intron-Containing Pre-mRNA	115	119	0.0E+00
Host Interactions of HIV factors	105	117	0.0E+00
The citric acid (TCA) cycle and respiratory electron transport	93	101	0.0E+00
mRNA Splicing - Major Pathway	90	92	0.0E+00
mRNA Splicing	90	92	0.0E+00
HIV Life Cycle	84	91	0.0E+00
RNA Polymerase II Transcription	82	89	0.0E+00
Influenza Infection	80	88	0.0E+00
Regulation of mRNA Stability by Proteins that Bind AU-rich Elements	80	94	2.5E-03
Late Phase of HIV Life Cycle	77	82	0.0E+00
Influenza Life Cycle	76	83	0.0E+00
Respiratory electron transport, ATP synthesis by chemiosmotic coupling, and heat production by uncoupling proteins.	72	78	0.0E+00
Translation	67	77	1.0E-03
Class I MHC mediated antigen processing & presentation	64	75	2.5E-03
Respiratory electron transport	61	65	0.0E+00
Antigen processing-Cross presentation	60	71	4.5E-03
Eukaryotic Translation Initiation	60	70	6.0E-03
Cap-dependent Translation Initiation	60	70	6.0E-03
ER-Phagosome pathway	58	68	3.5E-03
Signalling by NGF	56	69	9.0E-03
Signaling by Wnt	55	66	8.5E-03
Degradation of beta-catenin by the destruction complex	55	66	8.5E-03
Vif-mediated degradation of APOBEC3G	54	64	6.0E-03
Destabilization of mRNA by AUF1 (hnRNP D0)	54	64	4.0E-03
Vpu mediated degradation of CD4	53	63	3.5E-03
SCF-beta-TrCP mediated degradation of Emi1	53	63	5.0E-03
SCF(Skp2)-mediated degradation of p27/p21	52	61	6.0E-03
Nonsense-Mediated Decay	52	60	7.5E-03
Nonsense Mediated Decay Enhanced by the Exon Junction Complex	52	60	7.5E-03
Transcription of the HIV genome	50	54	3.5E-03
Autodegradation of Cdh1 by Cdh1:APC/C	49	56	2.0E-03
RNA Polymerase II Pre-transcription Events	48	52	1.5E-03
Transport of Mature Transcript to Cytoplasm	47	49	0.0E+00
Transport of Mature mRNA derived from an Intron-Containing Transcript	43	45	5.0E-04
Ceramide signaling pathway	39	48	8.5E-03
RNA Polymerase II Transcription Elongation	35	36	2.0E-03
Formation of RNA Pol II elongation complex	35	36	2.0E-03
Formation of HIV-1 elongation complex in the absence of HIV-1 Tat	35	36	2.0E-03
TCR signaling	34	37	1.5E-03
Tat-mediated elongation of the HIV-1 transcript	34	35	5.0E-04
RNA Polymerase II Transcription Termination	34	37	5.0E-03
Formation of HIV-1 elongation complex containing HIV-1 Tat	34	35	5.0E-04
Cleavage of Growing Transcript in the Termination Region	34	37	5.0E-03
HIV-1 Transcription Elongation	34	35	5.0E-04
Post-Elongation Processing of the Transcript	34	37	5.0E-03
Toll Like Receptor 9 (TLR9) Cascade	33	38	2.0E-03
mRNA Splicing - Minor Pathway	31	31	1.0E-03
mRNA 3'-end processing	31	32	0.0E+00
Post-Elongation Processing of Intron-Containing pre-mRNA	31	32	0.0E+00

HIV-1 Nef: Negative effector of Fas and TNF-alpha	30	35	2.0E-03
Transport of Mature mRNAs Derived from Intronless Transcripts	30	32	9.5E-03
MyD88:Mal cascade initiated on plasma membrane	29	35	4.0E-03
Toll Like Receptor 7/8 (TLR7/8) Cascade	28	33	1.0E-03
MyD88 dependent cascade initiated on endosome	28	33	1.0E-03
Interactions of Rev with host cellular proteins	28	29	6.0E-03
Toll Like Receptor 5 (TLR5) Cascade	27	33	8.5E-03
Toll Like Receptor 10 (TLR10) Cascade	27	33	5.5E-03
MyD88 cascade initiated on plasma membrane	27	32	2.5E-03
TRAF6 mediated induction of NFkB and MAP kinases upon TLR7/8 or 9 activation	27	32	2.5E-03
Rev-mediated nuclear export of HIV-1 RNA	27	28	6.0E-03
Pausing and recovery of HIV-1 elongation	25	25	1.5E-03
Nuclear import of Rev protein	25	26	9.5E-03
Elongation arrest and recovery	25	25	1.5E-03
Pausing and recovery of elongation	25	25	1.5E-03
HIV-1 elongation arrest and recovery	25	25	1.5E-03
Tat-mediated HIV-1 elongation arrest and recovery	24	24	2.5E-03
Pausing and recovery of Tat-mediated HIV-1 elongation	24	24	2.5E-03
Downstream TCR signaling	24	26	1.5E-03
Cytosolic tRNA aminoacylation	23	24	7.5E-03
p75 NTR receptor-mediated signalling	22	24	2.5E-03
Abortive elongation of HIV-1 transcript in the absence of Tat	17	17	0.0E+00
Arf1 pathway	16	18	7.5E-03
The role of Nef in HIV-1 replication and disease pathogenesis	16	17	1.0E-03
MAP kinase activation in TLR cascade	15	16	6.5E-03
MicroRNA (miRNA) Biogenesis	14	14	0.0E+00
Regulatory RNA pathways	14	14	0.0E+00
Generation of second messenger molecules	13	14	6.5E-03
Nef-mediates down modulation of cell surface receptors by recruiting them to clathrin adapters	12	12	0.0E+00
Viral Messenger RNA Synthesis	10	10	0.0E+00
Sumoylation by RanBP2 regulates transcriptional repression	10	10	0.0E+00
Antigen Presentation: Folding, assembly and peptide loading of class I MHC	10	10	0.0E+00
Folding of actin by CCT/TriC	9	9	5.0E-04
Recycling of eIF2:GDP	8	8	0.0E+00
MAPK targets/ Nuclear events mediated by MAP kinases	8	8	5.0E-04
Phosphorylation of CD3 and TCR zeta chains	8	8	5.0E-04
N-glycan trimming in the ER and Calnexin/Calreticulin cycle	8	8	0.0E+00
Formation of tubulin folding intermediates by CCT/TriC	8	8	5.0E-04
Calnexin/calreticulin cycle	7	7	0.0E+00
Purine salvage	7	7	0.0E+00
GDP-mannose biosynthesis	6	6	1.0E-03
Pyruvate metabolism	6	6	3.5E-03
Alternative NF-kappaB pathway	6	6	1.5E-03
Translocation of ZAP-70 to Immunological synapse	6	6	4.0E-03
Post-chaperonin tubulin folding pathway	5	5	2.0E-03
COPII (Coat Protein 2) Mediated Vesicle Transport	5	5	9.0E-03
Activation of DNA fragmentation factor	5	5	1.0E-03
ER to Golgi Transport	5	5	9.0E-03
Activation of Chaperones by IRE1alpha	5	5	0.0E+00
Beta oxidation of hexanoyl-CoA to butanoyl-CoA	5	5	2.0E-03
Beta oxidation of decanoyl-CoA to octanoyl-CoA-CoA	5	5	2.5E-03
Beta oxidation of octanoyl-CoA to hexanoyl-CoA	5	5	2.5E-03
Nef mediated downregulation of MHC class I complex cell surface expression	5	5	5.0E-03
Phospholipase C-mediated cascade	5	5	2.0E-03
Apoptosis induced DNA fragmentation	5	5	1.0E-03
DAG and IP3 signaling	5	5	2.0E-03
Regulation of pyruvate dehydrogenase (PDH) complex	5	5	8.5E-03
Nuclear Events (kinase and transcription factor activation)	5	5	7.5E-03

CD20⁺ B cells - Pathway Commons	No. in selected genes	No. in all RefSeq	*p*-value
Gene Expression	189	297	0.0E+00
Metabolism of RNA	119	212	0.0E+00
HIV Infection	112	173	0.0E+00
Formation and Maturation of mRNA Transcript	105	164	0.0E+00
Metabolism of mRNA	97	176	1.0E-03
Metabolism of proteins	97	180	7.0E-03
TNF alpha/NF-kB	89	159	5.0E-04
mRNA Processing	85	136	0.0E+00
Apoptosis	78	144	0.0E+00
Host Interactions of HIV factors	75	117	0.0E+00
Processing of Capped Intron-Containing Pre-mRNA	74	119	0.0E+00
S Phase	65	115	1.0E-03
HIV Life Cycle	62	91	0.0E+00
FAS (CD95) signaling pathway	62	120	3.5E-03
The citric acid (TCA) cycle and respiratory electron transport	60	101	1.0E-03
G1/S Transition	60	104	0.0E+00
Late Phase of HIV Life Cycle	58	82	0.0E+00
Regulation of mRNA Stability by Proteins that Bind AU-rich Elements	57	94	0.0E+00
Cell Cycle Checkpoints	57	106	4.5E-03
RNA Polymerase II Transcription	57	89	0.0E+00
mRNA Splicing - Major Pathway	54	92	5.0E-04
mRNA Splicing	54	92	5.0E-04
Cyclin A:Cdk2-associated events at S phase entry	53	84	0.0E+00
Class I MHC mediated antigen processing & presentation	50	75	0.0E+00
Influenza Life Cycle	49	83	5.0E-03
Cyclin E associated events during G1/S transition	49	74	0.0E+00
Respiratory electron transport, ATP synthesis by chemiosmotic coupling, and heat production by uncoupling proteins.	47	78	2.0E-03
Antigen processing-Cross presentation	46	71	0.0E+00
Translation	45	77	4.5E-03
Switching of origins to a post-replicative state	44	75	0.0E+00
Removal of licensing factors from origins	44	77	5.0E-04
Regulation of DNA replication	44	77	5.0E-04
Orc1 removal from chromatin	44	75	0.0E+00
Regulation of mitotic cell cycle	43	73	0.0E+00
APC/C-mediated degradation of cell cycle proteins	43	73	0.0E+00
Regulation of Apoptosis	43	70	0.0E+00
Assembly of the pre-replicative complex	43	75	1.0E-03
ER-Phagosome pathway	43	68	0.0E+00
p53-Dependent G1 DNA Damage Response	42	67	0.0E+00
Regulation of APC/C activators between G1/S and early anaphase	42	72	1.5E-03
p53-Dependent G1/S DNA damage checkpoint	42	67	0.0E+00
Respiratory electron transport	42	65	5.0E-04
Destabilization of mRNA by AUF1 (hnRNP D0)	42	64	0.0E+00
G1/S DNA Damage Checkpoints	42	70	0.0E+00
SCF(Skp2)-mediated degradation of p27/p21	41	61	0.0E+00
Signaling by Wnt	41	66	0.0E+00
BCR signaling pathway	41	63	0.0E+00
Degradation of beta-catenin by the destruction complex	41	66	0.0E+00
CDT1 association with the CDC6:ORC:origin complex	41	69	0.0E+00
Autodegradation of the E3 ubiquitin ligase COP1	40	64	0.0E+00
Activation of APC/C and APC/C:Cdc20 mediated degradation of mitotic proteins	40	68	1.0E-03
APC/C:Cdc20 mediated degradation of mitotic proteins	40	67	0.0E+00
Regulation of ornithine decarboxylase (ODC)	40	63	0.0E+00
Vif-mediated degradation of APOBEC3G	40	64	0.0E+00
SCF-beta-TrCP mediated degradation of Emi1	40	63	0.0E+00
Stabilization of p53	40	65	0.0E+00
Vpu mediated degradation of CD4	39	63	0.0E+00
Ubiquitin-dependent degradation of Cyclin D1	39	62	0.0E+00
Cdc20:Phospho-APC/C mediated degradation of Cyclin A	39	65	0.0E+00
p53-Independent DNA Damage Response	39	65	0.0E+00
p53-Independent G1/S DNA damage checkpoint	39	65	0.0E+00
APC/C:Cdc20 mediated degradation of Securin	39	62	0.0E+00
Ubiquitin-dependent degradation of Cyclin D	39	62	0.0E+00
Regulation of activated PAK-2p34 by proteasome mediated degradation	39	62	0.0E+00
Ubiquitin Mediated Degradation of Phosphorylated Cdc25A	39	64	0.0E+00
CDK-mediated phosphorylation and removal of Cdc6	39	62	0.0E+00
Cross-presentation of soluble exogenous antigens (endosomes)	38	60	0.0E+00
APC/C:Cdh1 mediated degradation of Cdc20 and other APC/C:Cdh1 targeted proteins in late mitosis/early G1	38	60	0.0E+00
Antigen processing: Ubiquitination & Proteasome degradation	38	60	0.0E+00
Transcription of the HIV genome	38	54	0.0E+00
RNA Polymerase II Pre-transcription Events	37	52	0.0E+00
Autodegradation of Cdh1 by Cdh1:APC/C	36	56	0.0E+00
Signaling events regulated by Ret tyrosine kinase	35	64	6.0E-03
Transport of Mature Transcript to Cytoplasm	33	49	0.0E+00
Fc-epsilon receptor I signaling in mast cells	33	59	8.5E-03
Toll Receptor Cascades	31	55	2.5E-03
tRNA Aminoacylation	30	41	0.0E+00
Transport of Mature mRNA derived from an Intron-Containing Transcript	29	45	5.0E-04
Asparagine N-linked glycosylation	29	47	1.0E-03
Formation of HIV-1 elongation complex in the absence of HIV-1	27	36	0.0E+00
RNA Polymerase II Transcription Elongation	27	36	0.0E+00
Formation of RNA Pol II elongation complex	27	36	0.0E+00
Ceramide signaling pathway	27	48	2.5E-03
Formation of HIV-1 elongation complex containing HIV-1 Tat	26	35	0.0E+00
HIV-1 Transcription Elongation	26	35	0.0E+00
Tat-mediated elongation of the HIV-1 transcript	26	35	0.0E+00
Metabolism of non-coding RNA	25	39	1.0E-03
snRNP Assembly	25	39	1.0E-03
Caspase cascade in apoptosis	25	45	3.5E-03
RNA Polymerase II Transcription Pre-Initiation And Promoter Opening	24	35	0.0E+00
RNA Polymerase II Promoter Escape	24	35	0.0E+00
Transport of Mature mRNAs Derived from Intronless Transcripts	24	32	0.0E+00
HIV-1 Transcription Initiation	24	35	0.0E+00
RNA Polymerase II Transcription Initiation And Promoter Clearance	24	35	0.0E+00
RNA Polymerase II HIV-1 Promoter Escape	24	35	0.0E+00
RNA Polymerase II Transcription Initiation	24	35	0.0E+00

Toll Like Receptor 9 (TLR9) Cascade	23	38	3.0E-03
Transport of Mature mRNA Derived from an Intronless Transcript	23	31	0.0E+00
HIV-1 Nef: Negative effector of Fas and TNF-alpha	22	35	0.0E+00
Activated TLR4 signalling	22	39	8.0E-03
Toll Like Receptor 2 (TLR2) Cascade	22	37	2.0E-03
Toll Like Receptor TLR6:TLR2 Cascade	22	37	2.0E-03
MyD88:Mal cascade initiated on plasma membrane	21	35	1.0E-03
Toll Like Receptor TLR1:TLR2 Cascade	21	36	2.5E-03
Transport of the SLBP Dependant Mature mRNA	21	29	0.0E+00
Global Genomic NER (GG-NER)	21	33	7.5E-03
Toll Like Receptor 7/8 (TLR7/8) Cascade	20	33	1.5E-03
MyD88 dependent cascade initiated on endosome	20	33	1.5E-03
Interactions of Rev with host cellular proteins	20	29	0.0E+00
Rev-mediated nuclear export of HIV-1 RNA	20	28	0.0E+00
Transport of the SLBP independent Mature mRNA	20	28	0.0E+00
Toll Like Receptor 10 (TLR10) Cascade	20	33	5.0E-04
RIG-I/MDA5 mediated induction of IFN-alpha/beta pathways	20	35	3.0E-03
Toll Like Receptor 5 (TLR5) Cascade	19	33	8.0E-03
Nuclear import of Rev protein	19	26	0.0E+00
FoxO family signaling	19	29	0.0E+00
EPO signaling pathway	19	31	7.0E-03
Vpr-mediated nuclear import of PICs	19	25	0.0E+00
TRAF6 mediated induction of NFkB and MAP kinases upon TLR7/8 or 9 activation	19	32	1.0E-03
NEP/NS2 Interacts with the Cellular Export Machinery	19	25	0.0E+00
Formation of the HIV-1 Early Elongation Complex	19	26	0.0E+00
Interactions of Vpr with host cellular proteins	19	25	0.0E+00
MyD88 cascade initiated on plasma membrane	19	32	1.0E-03
Export of Viral Ribonucleoproteins from Nucleus	19	25	0.0E+00
Formation of the Early Elongation Complex	19	26	0.0E+00
Hexose transport	19	29	0.0E+00
Cytosolic tRNA aminoacylation	19	24	0.0E+00
Transport of Ribonucleoproteins into the Host Nucleus	18	25	0.0E+00
Glucose transport	18	27	0.0E+00
RNA Polymerase I Transcription	18	28	7.5E-03
Elongation arrest and recovery	17	25	0.0E+00
RNA Polymerase I Promoter Clearance	17	26	0.0E+00
Regulation of Glucokinase by Glucokinase Regulatory Protein	17	25	0.0E+00
mRNA Capping	17	23	0.0E+00
Pausing and recovery of HIV-1 elongation	17	25	0.0E+00
Osteopontin-mediated events	17	26	5.0E-04
Pausing and recovery of elongation	17	25	0.0E+00
RNA Polymerase I Transcription Initiation	17	24	0.0E+00
Pyruvate metabolism and Citric Acid (TCA) cycle	17	27	9.5E-03
HIV-1 elongation arrest and recovery	17	25	0.0E+00
Tat-mediated HIV-1 elongation arrest and recovery	16	24	0.0E+00
Pausing and recovery of Tat-mediated HIV-1 elongation	16	24	0.0E+00
Mitotic Metaphase/Anaphase Transition	16	22	0.0E+00
Downstream TCR signaling	16	26	3.5E-03
Trk receptor signaling mediated by the MAPK pathway	15	24	8.0E-03
RNA Polymerase I Chain Elongation	15	20	0.0E+00
Formation of incision complex in GG-NER	15	20	0.0E+00
Protein folding	15	21	0.0E+00
Dual incision reaction in GG-NER	15	20	0.0E+00
RNA Pol II CTD phosphorylation and interaction with CE - 11	15	21	0.0E+00
RNA Polymerase I Transcription Termination	15	21	0.0E+00
RNA Pol II CTD phosphorylation and interaction with CE	15	21	0.0E+00
Citric acid cycle (TCA cycle)	14	19	0.0E+00
RNA Polymerase I Promoter Escape	14	20	0.0E+00
Mitochondrial tRNA aminoacylation	13	20	0.0E+00
Chaperonin-mediated protein folding	12	16	0.0E+00
Cooperation of Prefoldin and TriC/CCT in actin and tubulin folding	12	15	0.0E+00
Abortive elongation of HIV-1 transcript in the absence of Tat	11	17	0.0E+00
Prefoldin mediated transfer of substrate to CCT/TriC	11	14	0.0E+00
Destabilization of mRNA by Tristetraprolin (TTP)	11	17	0.0E+00
MicroRNA (miRNA) Biogenesis	10	14	0.0E+00
Regulatory RNA pathways	10	14	0.0E+00
Transport to the Golgi and subsequent modification	10	15	2.0E-03
Processing of Intronless Pre-mRNAs	10	14	0.0E+00
Conversion from APC/C:Cdc20 to APC/C:Cdh1 in late anaphase	10	16	1.0E-03
Synthesis and interconversion of nucleotide di- and triphosphates	10	16	1.5E-03
Cyclin D associated events in G1	9	13	0.0E+00
S1P2 pathway	9	13	5.0E-04
G1 Phase	9	13	0.0E+00
Atypical NF-kappaB pathway	9	14	3.5E-03
Folding of actin by CCT/TriC	8	9	0.0E+00
DNA-PK pathway in nonhomologous end joining	8	13	7.5E-03
TCA cycle variation III (eukaryotic)	8	13	0.0E+00
mRNA Decay by 3' to 5' Exoribonuclease	8	11	0.0E+00
Metabolism of polyamines	8	13	1.0E-03
Extrinsic Pathway for Apoptosis	8	13	7.5E-03
Death Receptor Signalling	8	13	7.5E-03
superpathway of geranylgeranyldiphosphate biosynthesis I (via mevalonate)	8	13	2.0E-03
Recycling of eIF2:GDP	7	8	0.0E+00
Antigen Presentation: Folding, assembly and peptide loading of class I MHC	7	10	4.0E-03
Association of TriC/CCT with target proteins during biosynthesis	7	9	2.0E-03
MAPK targets/ Nuclear events mediated by MAP kinases	7	8	0.0E+00
p75NTR signals via NF-kB	7	11	7.5E-03
isoleucine degradation I	7	11	2.5E-03
Formation of tubulin folding intermediates by CCT/TriC	7	8	0.0E+00
Viral dsRNA:TLR3:TRIF Complex Activates RIP1	6	9	2.0E-03
dolichyl-diphosphooligosaccharide biosynthesis	6	7	0.0E+00
NF-kB activation through FADD/RIP-1 pathway mediated by caspase-8 and -10	6	8	5.0E-03
ER to Golgi Transport	5	5	5.0E-04
Beta oxidation of decanoyl-CoA to octanoyl-CoA-CoA	5	5	0.0E+00
COPII (Coat Protein 2) Mediated Vesicle Transport	5	5	5.0E-04
DARPP-32 events	5	6	5.5E-03
Beta oxidation of octanoyl-CoA to hexanoyl-CoA	5	5	0.0E+00
TRAF6 mediated NF-kB activation	5	7	8.5E-03
Purine salvage	5	7	7.0E-03
Activation of DNA fragmentation factor	4	5	2.0E-03
Beta oxidation of lauroyl-CoA to decanoyl-CoA-CoA	4	5	3.0E-03
GRB2:SOS provides linkage to MAPK signaling for Intergrins	4	5	5.0E-03
Beta-oxidation of very long chain fatty acids	4	5	4.5E-03
Apoptosis induced DNA fragmentation	4	5	2.0E-03
2-ketoglutarate dehydrogenase complex	4	4	5.0E-04
Beta oxidation of hexanoyl-CoA to butanoyl-CoA	4	5	5.5E-03
mitochondrial fatty acid beta-oxidation of unsaturated fatty acids	4	5	5.0E-03
CREB phosphorylation	4	4	2.0E-03
Utilization of Ketone Bodies	3	3	6.0E-03
DNA Damage Reversal	3	3	5.5E-03
Activation of the AP-1 family of transcription factors	3	3	4.5E-03

Table S7. Differences of functional enrichments between gene expression information alone (highly expressed genes) and putative transcriptional target genes using Pathway Commons.

Monocytes - functional annotations enriched only in transcriptional target genes using Pathway Commons	No. of TF
Proteoglycan syndecan-mediated signaling events	18
Regulation of CDC42 activity	15
LKB1 signaling events	14
Glypican pathway	13
Interferon Signaling	13
Sphingosine 1-phosphate (S1P) pathway	13
IL5-mediated signaling events	12
Syndecan-1-mediated signaling events	12
IL3-mediated signaling events	11
Mitotic Prophase	11
Golgi Cisternae Pericentriolar Stack Reorganization	11
Interferon alpha/beta signaling	11
IFN-gamma pathway	10
Signaling events mediated by Hepatocyte Growth Factor Receptor (c-Met)	10
Recruitment of mitotic centrosome proteins and complexes	10
GMCSF-mediated signaling events	10
CDC42 signaling events	10
Centrosome maturation	10
ATR signaling pathway	10
Thrombin/protease-activated receptor (PAR) pathway	9
Antigen processing-Cross presentation	9
ErbB receptor signaling network	9
IL23-mediated signaling events	9
VEGF and VEGFR signaling network	9
Class I MHC mediated antigen processing & presentation	9
Alpha9 beta1 integrin signaling events	9
Endothelins	9
PAR1-mediated thrombin signaling events	9
Signaling events mediated by VEGFR1 and VEGFR2	9
Integrin family cell surface interactions	9
Plasma membrane estrogen receptor signaling	9
Nectin adhesion pathway	9
Beta1 integrin cell surface interactions	9
Internalization of ErbB1	8
G2/M Transition	8
Urokinase-type plasminogen activator (uPA) and uPAR-mediated signaling	8
Loss of Nlp from mitotic centrosomes	8
Arf6 signaling events	8
EGFR-dependent Endothelin signaling events	8
ER-Phagosome pathway	8
IGF1 pathway	8
ErbB1 downstream signaling	8
Loss of proteins required for interphase microtubule organization from the centrosome	8
Arf6 trafficking events	8
Signaling events mediated by focal adhesion kinase	8
Class I PI3K signaling events mediated by Akt	8
mTOR signaling pathway	8
Insulin Pathway	8
Arf6 downstream pathway	8
PDGFR-beta signaling pathway	8
PDGF receptor signaling network	8
Glypican 1 network	8
EGF receptor (ErbB1) signaling pathway	8
Class I PI3K signaling events	8
S1P1 pathway	8
AP-1 transcription factor network	7
Mitotic G2-G2/M phases	7
Signaling events mediated by HDAC Class II	7
Immune System	7
p53 pathway	6
Direct p53 effectors	6
TRAIL signaling pathway	6
Processing of Capped Intronless Pre-mRNA	6
Post-Elongation Processing of Intronless pre-mRNA	6
CXCR4-mediated signaling events	6
HIF-1-alpha transcription factor network	6
Processing of Intronless Pre-mRNAs	5
Caspase cascade in apoptosis	5
Trk receptor signaling mediated by the MAPK pathway	5
CD40/CD40L signaling	5
FAS (CD95) signaling pathway	5
Canonical NF-kappaB pathway	5
IL4-mediated signaling events	5
Alpha-synuclein signaling	5
Posttranslational regulation of adherens junction stability and dissassembly	4
M Phase	4
Alpha4 beta1 integrin signaling events	4
Validated nuclear estrogen receptor alpha network	4
IL8- and CXCR1-mediated signaling events	4
Signaling events mediated by Stem cell factor receptor (c-Kit)	4
Caspase-mediated cleavage of cytoskeletal proteins	4
ATM pathway	4
Signaling events regulated by Ret tyrosine kinase	4
BMP receptor signaling	4
IL12-mediated signaling events	4
Adaptive Immune System	3
Noncanonical Wnt signaling pathway	3
Wnt signaling network	3
fatty acid beta-oxidation I	3
Clathrin derived vesicle budding	3
Cytokine Signaling in Immune system	3
Regulation of ornithine decarboxylase (ODC)	3
IL6-mediated signaling events	3
Nonsense Mediated Decay Independent of the Exon Junction Complex	3
trans-Golgi Network Vesicle Budding	3
Fatty acid, triacylglycerol, and ketone body metabolism	3
Vif-mediated degradation of APOBEC3G	3
Integrin-linked kinase signaling	3
Glypican 3 network	3
p38 MAPK signaling pathway	3
Syndecan-4-mediated signaling events	3
Signaling events mediated by TCPTP	3
Hypoxic and oxygen homeostasis regulation of HIF-1-alpha	3
amb2 Integrin signaling	3

Monocytes - functional annotations enriched only in gene expression information alone (highly expressed genes) using Pathway Commons	No. in selected genes	No. in all RefSeq	*p*-value
Gene Expression	161	297	0.0E+00
Formation and Maturation of mRNA Transcript	89	164	1.5E-03
The citric acid (TCA) cycle and respiratory electron transport	59	101	0.0E+00
Androgen-mediated signaling	54	106	1.0E-03
HIV Life Cycle	51	91	7.0E-03
Regulation of Androgen receptor activity	49	96	2.5E-03
Late Phase of HIV Life Cycle	47	82	4.5E-03
Respiratory electron transport, ATP synthesis by chemiosmotic coupling, and heat production by uncoupling proteins.	45	78	3.0E-03
Respiratory electron transport	40	65	0.0E+00
BCR signaling pathway	36	63	0.0E+00
Signaling by Interleukins	36	70	9.0E-03
Endogenous TLR signaling	33	56	0.0E+00
Fc-epsilon receptor I signaling in mast cells	33	59	2.0E-03
RNA Polymerase II Pre-transcription Events	31	52	9.5E-03
Toll Receptor Cascades	31	55	3.5E-03
tRNA Aminoacylation	26	41	0.0E+00
Toll Like Receptor 4 (TLR4) Cascade	25	43	1.0E-03
Activated TLR4 signalling	22	39	3.5E-03
HIV-1 Nef: Negative effector of Fas and TNF-alpha	21	35	2.0E-03
RIG-I/MDA5 mediated induction of IFN-alpha/beta pathways	21	35	1.0E-03
Toll Like Receptor 2 (TLR2) Cascade	21	37	5.0E-03
Toll Like Receptor TLR6:TLR2 Cascade	21	37	5.0E-03
Toll Like Receptor TLR1:TLR2 Cascade	20	36	5.0E-03
FoxO family signaling	19	29	0.0E+00
Pyruvate metabolism and Citric Acid (TCA) cycle	18	27	0.0E+00
Cytosolic tRNA aminoacylation	18	24	0.0E+00
Vpr-mediated nuclear import of PICs	16	25	0.0E+00
Osteopontin-mediated events	16	26	1.0E-03
Interactions of Vpr with host cellular proteins	16	25	0.0E+00
Protein folding	15	21	0.0E+00
Citric acid cycle (TCA cycle)	13	19	0.0E+00
Chaperonin-mediated protein folding	12	16	0.0E+00
Cooperation of Prefoldin and TriC/CCT in actin and tubulin folding	12	15	0.0E+00
Prefoldin mediated transfer of substrate to CCT/TriC	11	14	0.0E+00
Extrinsic Pathway for Apoptosis	10	13	0.0E+00
Death Receptor Signalling	10	13	0.0E+00
GPVI-mediated activation cascade	9	15	4.0E-03
S1P2 pathway	8	13	5.0E-04
EPHA2 forward signaling	8	13	3.5E-03
Folding of actin by CCT/TriC	8	9	0.0E+00

Association of TriC/CCT with target proteins during biosynthesis	7	9	1.0E-03
heme biosynthesis II	7	10	0.0E+00
isoleucine degradation I	7	11	4.5E-03
Formation of tubulin folding intermediates by CCT/TriC	7	8	0.0E+00
Caspase-8 is formed from procaspase-8	6	9	6.0E-03
COPI Mediated Transport	6	9	9.0E-03
NF-kB activation through FADD/RIP-1 pathway mediated by caspase-8 and -10	6	8	3.0E-03
Golgi to ER Retrograde Transport	6	9	9.0E-03
Activation of Pro-Caspase 8	6	9	6.0E-03
IKK complex recruitment mediated by RIP1	6	7	5.0E-04
Heme biosynthesis	5	6	1.0E-03
Metabolism of porphyrins	5	7	2.0E-03
TRAF6 mediated NF-kB activation	5	7	7.0E-03
Beta oxidation of lauroyl-CoA to decanoyl-CoA-CoA	4	5	2.0E-03
Beta oxidation of hexanoyl-CoA to butanoyl-CoA	4	5	2.0E-03
Beta oxidation of decanoyl-CoA to octanoyl-CoA-CoA	4	5	2.5E-03
Beta oxidation of octanoyl-CoA to hexanoyl-CoA	4	5	2.5E-03
GRB2:SOS provides linkage to MAPK signaling for Intergrins	4	5	2.0E-03
G alpha (q) signalling events	4	4	1.0E-03
Beta-oxidation of very long chain fatty acids	4	5	4.0E-03
Interleukin receptor SHC signaling	4	4	5.5E-03
AKT phosphorylates targets in the nucleus	3	3	8.5E-03
DNA Damage Reversal	3	3	7.0E-03

CD4$^+$ T cells - functional annotations enriched only in transcriptional target genes using Pathway Commons	No. of TF
TCR signaling in naive CD8$^+$ T cells	36
IL12-mediated signaling events	24
Downstream signaling in naive CD8$^+$ T cells	21
TCR signaling in naive CD4$^+$ T cells	21
IL12 signaling mediated by STAT4	20
Validated transcriptional targets of AP1 family members Fra1 and Fra2	19
CXCR4-mediated signaling events	17
ATF-2 transcription factor network	15
Thrombin/protease-activated receptor (PAR) pathway	14
PAR1-mediated thrombin signaling events	14
Internalization of ErbB1	13
Urokinase-type plasminogen activator (uPA) and uPAR-mediated signaling	13
ErbB receptor signaling network	13
Arf6 signaling events	13
VEGF and VEGFR signaling network	13
EGFR-dependent Endothelin signaling events	13
ErbB1 downstream signaling	13
Signaling events mediated by VEGFR1 and VEGFR2	13
salvage pathways of pyrimidine ribonucleotides	13
Transcription from mitochondrial promoters	13
Arf6 trafficking events	13
Signaling events mediated by focal adhesion kinase	13
Class I PI3K signaling events mediated by Akt	13
mTOR signaling pathway	13
Integrin family cell surface interactions	13
Insulin Pathway	13
Arf6 downstream pathway	13
PDGFR-beta signaling pathway	13
Glypican 1 network	13
EGF receptor (ErbB1) signaling pathway	13
Class I PI3K signaling events	13
S1P1 pathway	13
IFN-gamma pathway	12
Alpha9 beta1 integrin signaling events	12
Extrinsic Pathway for Apoptosis	12
Death Receptor Signalling	12
IL5-mediated signaling events	12
CD40/CD40L signaling	12
Beta1 integrin cell surface interactions	12
Signaling events mediated by Hepatocyte Growth Factor Receptor (c-Met)	11
LKB1 signaling events	11
IGF1 pathway	11
Syndecan-2-mediated signaling events	11
GMCSF-mediated signaling events	11
PDGF receptor signaling network	11
Plasma membrane estrogen receptor signaling	11
Nectin adhesion pathway	11
Canonical NF-kappaB pathway	11
Glypican pathway	10
Processing of Intronless Pre-mRNAs	10
IL2-mediated signaling events	10
FAS (CD95) signaling pathway	10
Signaling events regulated by Ret tyrosine kinase	10
Adaptive Immune System	9
Proteoglycan syndecan-mediated signaling events	9
Endothelins	9
Regulation of CDC42 activity	9
RIG-I/MDA5 mediated induction of IFN-alpha/beta pathways	9
Endogenous TLR signaling	9
Syndecan-1-mediated signaling events	9
Sphingosine 1-phosphate (S1P) pathway	9
Circadian rhythm pathway	9
IL23-mediated signaling events	8
TRAIL signaling pathway	8
IL3-mediated signaling events	8
Signaling events mediated by TCPTP	8
IL4-mediated signaling events	8
Costimulation by the CD28 family	7
AP-1 transcription factor network	7
Cytokine Signaling in Immune system	7
Integrin-linked kinase signaling	7
FGF signaling pathway	7
Interferon Signaling	7
BMAL1:CLOCK/NPAS2 Activates Gene Expression	7
Immune System	7
Calcium signaling in the CD4$^+$ TCR pathway	6
IL8-mediated signaling events	6
IL8- and CXCR2-mediated signaling events	6
Regulation of Apoptosis	6
Regulation of RAC1 activity	6
RAC1 signaling pathway	6
Circadian Clock	6
RhoA signaling pathway	6
Netrin-1 signaling	6
CDC42 signaling events	6
Immunoregulatory interactions between a Lymphoid and a non-Lymphoid cell	6
Cell junction organization	6
HIF-1-alpha transcription factor network	6
Cyclin E associated events during G1/S transition	6
Regulation of RhoA activity	6
Interferon alpha/beta signaling	6
p53-Dependent G1 DNA Damage Response	5
FoxO family signaling	5
G2/M Transition	5
Autodegradation of the E3 ubiquitin ligase COP1	5
Glutamate Neurotransmitter Release Cycle	5
Cyclin A:Cdk2-associated events at S phase entry	5
Regulation of APC/C activators between G1/S and early anaphase	5
APC/C-mediated degradation of cell cycle proteins	5
Signaling by Aurora kinases	5
Cdc20:Phospho-APC/C mediated degradation of Cyclin A	5
Activation of APC/C and APC/C:Cdc20 mediated degradation of mitotic proteins	5
p53-Independent DNA Damage Response	5
p53-Independent G1/S DNA damage checkpoint	5
APC/C:Cdc20 mediated degradation of mitotic proteins	5
Cross-presentation of soluble exogenous antigens (endosomes)	5
p53-Dependent G1/S DNA damage checkpoint	5
CDT1 association with the CDC6:ORC:origin complex	5
Assembly of the pre-replicative complex	5
APC/C:Cdh1 mediated degradation of Cdc20 and other APC/C:Cdh1 targeted proteins in late mitosis/early G1	5
Regulation of ornithine decarboxylase (ODC)	5
Antigen processing: Ubiquitination & Proteasome degradation	5
G1/S DNA Damage Checkpoints	5
Stabilization of p53	5
CDK-mediated phosphorylation and removal of Cdc6	5
Mitotic G2-G2/M phases	5
LPA receptor mediated events	5
Regulation of mitotic cell cycle	5
Ubiquitin-dependent degradation of Cyclin D1	5
Integrins in angiogenesis	5
Signaling events mediated by HDAC Class I	5
a6b1 and a6b4 Integrin signaling	5
APC/C:Cdc20 mediated degradation of Securin	5
Ubiquitin-dependent degradation of Cyclin D	5
Calcineurin-regulated NFAT-dependent transcription in lymphocytes	5
Metabolism of lipids and lipoproteins	5
Regulation of activated PAK-2p34 by proteasome mediated degradation	5
Canonical Wnt signaling pathway	5
Regulation of nuclear beta catenin signaling and target gene transcription	5
Ubiquitin Mediated Degradation of Phosphorylated Cdc25A	5
Wnt	5
Validated targets of C-MYC transcriptional repression	4
Switching of origins to a post-replicative state	4
Innate Immune System	4
Negative regulators of RIG-I/MDA5 signaling	4
Insulin-mediated glucose transport	4
Wnt signaling network	4

Cell surface interactions at the vascular wall	4
EGFR interacts with phospholipase C-gamma	4
p75(NTR)-mediated signaling	4
Regulation of DNA replication	4
DNA Replication Pre-Initiation	4
Orc1 removal from chromatin	4
IL6-mediated signaling events	4
M Phase	4
Osteopontin-mediated events	4
Signaling events mediated by HDAC Class III	4
KitReceptor	4
Validated nuclear estrogen receptor alpha network	4
Signaling events mediated by HDAC Class II	4
RNA Polymerase III Transcription Initiation From Type 2 Promoter	4
Glypican 3 network	4
IL2 signaling events mediated by PI3K	4
Removal of licensing factors from origins	4
M/G1 Transition	4
Factors involved in megakaryocyte development and platelet production	4
Atypical NF-kappaB pathway	4
RNA Polymerase III Transcription Initiation From Type 1 Promoter	4
Noncanonical Wnt signaling pathway	3
S Phase	3
EphrinA-EPHA pathway	3
Mitotic Prometaphase	3
G1 Phase	3
Regulation of retinoblastoma protein	3
RNA Polymerase III Transcription Initiation	3
eNOS activation	3
DNA Replication	3
Intrinsic Pathway for Apoptosis	3
EPO signaling pathway	3
Signaling events mediated by Stem cell factor receptor (c-Kit)	3
Cyclin D associated events in G1	3
Syndecan-4-mediated signaling events	3
NOTCH	3
Aurora A signaling	3
VEGFR1 specific signals	3
RNA Polymerase III Abortive And Retractive Initiation	3
G1/S Transition	3
EGFR1	3
RNA Polymerase III Transcription	3
mitochondrial fatty acid beta-oxidation of unsaturated fatty acids	3
Ras signaling in the CD4$^+$ TCR pathway	3
Androgen-mediated signaling	3
IL2 signaling events mediated by STAT5	3
CD28 co-stimulation	3
Cell Cycle, Mitotic	3

CD4$^+$ T cells - functional annotaions enriched only in gene expression information alone (highly expressed genes) using Pathway Commons	No. in selected genes	No. in all RefSeq	*p*-value
Gene Expression	271	297	0.0E+00
Metabolism of RNA	179	212	0.0E+00
HIV Infection	155	173	0.0E+00
Metabolism of mRNA	149	176	0.0E+00
TNF alpha/NF-kB	132	159	0.0E+00
mRNA Processing	128	136	0.0E+00
Processing of Capped Intron-Containing Pre-mRNA	115	119	0.0E+00
mRNA Splicing - Major Pathway	90	92	0.0E+00
mRNA Splicing	90	92	0.0E+00
HIV Life Cycle	84	91	0.0E+00
Influenza Infection	80	88	0.0E+00
Late Phase of HIV Life Cycle	77	82	0.0E+00
Influenza Life Cycle	76	83	0.0E+00
Respiratory electron transport, ATP synthesis by chemiosmotic coupling, and heat production by uncoupling proteins.	72	78	0.0E+00
Translation	67	77	1.0E-03
Respiratory electron transport	61	65	0.0E+00
Eukaryotic Translation Initiation	60	70	6.0E-03
Cap-dependent Translation Initiation	60	70	6.0E-03
Signalling by NGF	56	69	9.0E-03
Transcription of the HIV genome	50	54	3.5E-03
RNA Polymerase II Pre-transcription Events	48	52	1.5E-03
Transport of Mature Transcript to Cytoplasm	47	49	0.0E+00
Transport of Mature mRNA derived from an Intron-Containing Transcript	43	45	5.0E-04
Ceramide signaling pathway	39	48	8.5E-03
RNA Polymerase II Transcription Elongation	35	36	2.0E-03
Formation of RNA Pol II elongation complex	35	36	2.0E-03
Formation of HIV-1 elongation complex in the absence of HIV-1 Tat	35	36	2.0E-03
Tat-mediated elongation of the HIV-1 transcript	34	35	5.0E-04
RNA Polymerase II Transcription Termination	34	37	5.0E-03
Formation of HIV-1 elongation complex containing HIV-1 Tat	34	35	5.0E-04
Cleavage of Growing Transcript in the Termination Region	34	37	5.0E-03
HIV-1 Transcription Elongation	34	35	5.0E-04
Post-Elongation Processing of the Transcript	34	37	5.0E-03
mRNA Splicing - Minor Pathway	31	31	1.0E-03
HIV-1 Nef: Negative effector of Fas and TNF-alpha	30	35	2.0E-03
Transport of Mature mRNAs Derived from Intronless Transcripts	30	32	9.5E-03
MyD88:Mal cascade initiated on plasma membrane	29	35	4.0E-03
Toll Like Receptor 7/8 (TLR7/8) Cascade	28	33	1.0E-03
MyD88 dependent cascade initiated on endosome	28	33	1.0E-03
Interactions of Rev with host cellular proteins	28	29	6.0E-03
Toll Like Receptor 5 (TLR5) Cascade	27	33	8.5E-03
Toll Like Receptor 10 (TLR10) Cascade	27	33	5.5E-03
MyD88 cascade initiated on plasma membrane	27	32	2.5E-03
TRAF6 mediated induction of NFkB and MAP kinases upon TLR7/8 or 9 activation	27	32	2.5E-03
Rev-mediated nuclear export of HIV-1 RNA	27	28	6.0E-03
Pausing and recovery of HIV-1 elongation	25	25	1.5E-03
Nuclear import of Rev protein	25	26	9.5E-03
Elongation arrest and recovery	25	25	1.5E-03
Pausing and recovery of elongation	25	25	1.5E-03
HIV-1 elongation arrest and recovery	25	25	1.5E-03
Tat-mediated HIV-1 elongation arrest and recovery	24	24	2.5E-03
Pausing and recovery of Tat-mediated HIV-1 elongation	24	24	2.5E-03
Cytosolic tRNA aminoacylation	23	24	7.5E-03
Abortive elongation of HIV-1 transcript in the absence of Tat	17	17	0.0E+00
Arf1 pathway	16	18	7.5E-03
The role of Nef in HIV-1 replication and disease pathogenesis	16	17	1.0E-03
MAP kinase activation in TLR cascade	15	16	6.5E-03
MicroRNA (miRNA) Biogenesis	14	14	0.0E+00
Regulatory RNA pathways	14	14	0.0E+00
Nef-mediates down modulation of cell surface receptors by recruiting them to clathrin adapters	12	12	0.0E+00
Viral Messenger RNA Synthesis	10	10	0.0E+00
Antigen Presentation: Folding, assembly and peptide loading of class I MHC	10	10	0.0E+00
Folding of actin by CCT/TriC	9	9	5.0E-04
Recycling of eIF2:GDP	8	8	0.0E+00
MAPK targets/ Nuclear events mediated by MAP kinases	8	8	5.0E-04
N-glycan trimming in the ER and Calnexin/Calreticulin cycle	8	8	0.0E+00
Formation of tubulin folding intermediates by CCT/TriC	8	8	5.0E-04
Calnexin/calreticulin cycle	7	7	0.0E+00
Purine salvage	7	7	0.0E+00
GDP-mannose biosynthesis	6	6	1.0E-03
Pyruvate metabolism	6	6	3.5E-03
Post-chaperonin tubulin folding pathway	5	5	2.0E-03
COPII (Coat Protein 2) Mediated Vesicle Transport	5	5	9.0E-03
Activation of DNA fragmentation factor	5	5	1.0E-03
ER to Golgi Transport	5	5	9.0E-03
Activation of Chaperones by IRE1alpha	5	5	0.0E+00
Beta oxidation of hexanoyl-CoA to butanoyl-CoA	5	5	2.0E-03
Beta oxidation of decanoyl-CoA to octanoyl-CoA-CoA	5	5	2.5E-03
Beta oxidation of octanoyl-CoA to hexanoyl-CoA	5	5	2.5E-03
Nef mediated downregulation of MHC class I complex cell surface ex	5	5	5.0E-03
Apoptosis induced DNA fragmentation	5	5	1.0E-03
Regulation of pyruvate dehydrogenase (PDH) complex	5	5	8.5E-03
Nuclear Events (kinase and transcription factor activation)	5	5	7.5E-03

CD20$^+$ B cells - functional annotations enriched only in transcriptional target genes using Pathway Commons	No. of TF
Interferon alpha/beta signaling	12
Alpha6Beta4Integrin	11
Validated targets of C-MYC transcriptional activation	11
IL8- and CXCR2-mediated signaling events	10
IL6-mediated signaling events	9
Cell junction organization	9
Regulation of CDC42 activity	8
CXCR4-mediated signaling events	8
CDC42 signaling events	8
Syndecan-4-mediated signaling events	8
Noncanonical Wnt signaling pathway	7
Cell-Cell communication	7
TCR signaling in naive CD8$^+$ T cells	6
Wnt signaling network	6
Regulation of cytoplasmic and nuclear SMAD2/3 signaling	6
Regulation of nuclear SMAD2/3 signaling	6
Interleukin-1 signaling	6
Glypican 3 network	6
IL4-mediated signaling events	6
Canonical Wnt signaling pathway	6
TGF-beta receptor signaling	6
N-cadherin signaling events	6

Trk receptor signaling mediated by PI3K and PLC-gamma	5
Notch-mediated HES/HEY network	5
Posttranslational regulation of adherens junction stability and dissassembly	5
Notch signaling pathway	5
LPA receptor mediated events	5
ATF-2 transcription factor network	5
Mitotic Prophase	5
Signaling by Interleukins	5
Golgi Cisternae Pericentriolar Stack Reorganization	5
Interferon Signaling	5
Regulation of nuclear beta catenin signaling and target gene transcription	5
IL8-mediated signaling events	4
Proteoglycan syndecan-mediated signaling events	4
Transmission across Chemical Synapses	4
Loss of Nlp from mitotic centrosomes	4
GP1b-IX-V activation signalling	4
Role of Calcineurin-dependent NFAT signaling in lymphocytes	4
Signaling by GPCR	4
Nef and signal transduction	4
Loss of proteins required for interphase microtubule organization from the centrosome	4
Neuronal System	4
IL2-mediated signaling events	4
Signaling events mediated by TCPTP	4
Interleukin-3, 5 and GM-CSF signaling	4
Adaptive Immune System	3
Glucocorticoid receptor signaling	3
Stabilization and expansion of the E-cadherin adherens junction	3
Validated transcriptional targets of AP1 family members Fra1 and Fra2	3
Cytokine Signaling in Immune system	3
Arf1 pathway	3
E-cadherin signaling in the nascent adherens junction	3
E-cadherin signaling events	3
ALK1 signaling events	3
Syndecan-2-mediated signaling events	3
TCR signaling in naive CD4⁺ T cells	3
CD40/CD40L signaling	3
Regulation of p38-alpha and p38-beta	3
p38 MAPK signaling pathway	3
Integrins in angiogenesis	3
Polo-like kinase signaling events in the cell cycle	3
EGFR1	3
Calcineurin-regulated NFAT-dependent transcription in lymphocytes	3
ALK1 pathway	3
Glucocorticoid receptor regulatory network	3
Synthesis of DNA	3
Metabolism of lipids and lipoproteins	3
BMP receptor signaling	3
IL12-mediated signaling events	3

CD20⁺ B cells - functional annotations enriched only in gene expression information alone (highly expressed genes) using Pathway Commons	No. in selected genes	No. in all RefSeq	*p*-value
Gene Expression	189	297	0.0E+00
Metabolism of RNA	119	212	0.0E+00
HIV Infection	112	173	0.0E+00
Formation and Maturation of mRNA Transcript	105	164	0.0E+00
Metabolism of mRNA	97	176	1.0E-03
Metabolism of proteins	97	180	7.0E-03
mRNA Processing	85	136	0.0E+00
Processing of Capped Intron-Containing Pre-mRNA	74	119	0.0E+00
Late Phase of HIV Life Cycle	58	82	0.0E+00
RNA Polymerase II Transcription	57	89	0.0E+00
Influenza Life Cycle	49	83	5.0E-03
Transport of Mature Transcript to Cytoplasm	33	49	0.0E+00
Fc-epsilon receptor I signaling in mast cells	33	59	8.5E-03
tRNA Aminoacylation	30	41	0.0E+00
Transport of Mature mRNA derived from an Intron-Containing Transcript	29	45	5.0E-04
Formation of HIV-1 elongation complex in the absence of HIV-1 Tat	27	36	0.0E+00
RNA Polymerase II Transcription Elongation	27	36	0.0E+00
Formation of RNA Pol II elongation complex	27	36	0.0E+00
Formation of HIV-1 elongation complex containing HIV-1 Tat	26	35	0.0E+00
HIV-1 Transcription Elongation	26	35	0.0E+00
Tat-mediated elongation of the HIV-1 transcript	26	35	0.0E+00
Metabolism of non-coding RNA	25	39	1.0E-03
snRNP Assembly	25	39	1.0E-03
Transport of Mature mRNAs Derived from Intronless Transcripts	24	32	0.0E+00
Toll Like Receptor 9 (TLR9) Cascade	23	38	3.0E-03
Transport of Mature mRNA Derived from an Intronless Transcript	23	31	0.0E+00
HIV-1 Nef: Negative effector of Fas and TNF-alpha	22	35	0.0E+00
Activated TLR4 signalling	22	39	8.0E-03
Toll Like Receptor 2 (TLR2) Cascade	22	37	2.0E-03
Toll Like Receptor TLR6:TLR2 Cascade	22	37	2.0E-03
MyD88:Mal cascade initiated on plasma membrane	21	35	1.0E-03
Toll Like Receptor TLR1:TLR2 Cascade	21	36	2.5E-03
Transport of the SLBP Dependant Mature mRNA	21	29	0.0E+00
Global Genomic NER (GG-NER)	21	33	7.5E-03
Toll Like Receptor 7/8 (TLR7/8) Cascade	20	33	1.5E-03
MyD88 dependent cascade initiated on endosome	20	33	1.5E-03
Interactions of Rev with host cellular proteins	20	29	0.0E+00
Rev-mediated nuclear export of HIV-1 RNA	20	28	0.0E+00
Transport of the SLBP independent Mature mRNA	20	28	0.0E+00
Toll Like Receptor 10 (TLR10) Cascade	20	33	5.0E-04
Toll Like Receptor 5 (TLR5) Cascade	19	33	8.0E-03
Nuclear import of Rev protein	19	26	0.0E+00
FoxO family signaling	19	29	0.0E+00
Vpr-mediated nuclear import of PICs	19	25	0.0E+00
TRAF6 mediated induction of NFkB and MAP kinases upon TLR7/8 or 9 activation	19	32	1.0E-03
NEP/NS2 Interacts with the Cellular Export Machinery	19	25	0.0E+00
Interactions of Vpr with host cellular proteins	19	25	0.0E+00
MyD88 cascade initiated on plasma membrane	19	32	1.0E-03
Export of Viral Ribonucleoproteins from Nucleus	19	25	0.0E+00
Hexose transport	19	29	0.0E+00
Cytosolic tRNA aminoacylation	19	24	0.0E+00
Transport of Ribonucleoproteins into the Host Nucleus	18	25	0.0E+00
Glucose transport	18	27	0.0E+00
RNA Polymerase I Transcription	18	28	7.5E-03
RNA Polymerase I Promoter Clearance	17	26	0.0E+00
Regulation of Glucokinase by Glucokinase Regulatory Protein	17	25	0.0E+00
RNA Polymerase I Transcription Initiation	17	24	0.0E+00
Mitotic Metaphase/Anaphase Transition	16	22	0.0E+00
RNA Polymerase I Chain Elongation	15	20	0.0E+00
Formation of incision complex in GG-NER	15	20	0.0E+00
Dual incision reaction in GG-NER	15	20	0.0E+00
RNA Polymerase I Transcription Termination	15	21	0.0E+00
RNA Polymerase I Promoter Escape	14	20	0.0E+00
Mitochondrial tRNA aminoacylation	13	20	0.0E+00
Prefoldin mediated transfer of substrate to CCT/TriC	11	14	0.0E+00
Destabilization of mRNA by Tristetraprolin (TTP)	11	17	0.0E+00
Transport to the Golgi and subsequent modification	10	15	2.0E-03
Processing of Intronless Pre-mRNAs	10	14	0.0E+00
Synthesis and interconversion of nucleotide di- and triphosphates	10	16	1.5E-03
Cyclin D associated events in G1	9	13	0.0E+00
G1 Phase	9	13	0.0E+00
DNA-PK pathway in nonhomologous end joining	8	13	7.5E-03
mRNA Decay by 3' to 5' Exoribonuclease	8	11	0.0E+00
Extrinsic Pathway for Apoptosis	8	13	7.5E-03
Death Receptor Signalling	8	13	7.5E-03
superpathway of geranylgeranyldiphosphate biosynthesis I (via mevalonate)	8	13	2.0E-03
Recycling of eIF2:GDP	7	8	0.0E+00
Association of TriC/CCT with target proteins during biosynthesis	7	9	2.0E-03
MAPK targets/ Nuclear events mediated by MAP kinases	7	8	0.0E+00
Formation of tubulin folding intermediates by CCT/TriC	7	8	0.0E+00
Viral dsRNA:TLR3:TRIF Complex Activates RIP1	6	9	2.0E-03
dolichyl-diphosphooligosaccharide biosynthesis	6	7	0.0E+00
NF-kB activation through FADD/RIP-1 pathway mediated by caspase-8 and -10	6	8	5.0E-03
ER to Golgi Transport	5	5	5.0E-04
COPII (Coat Protein 2) Mediated Vesicle Transport	5	5	5.0E-04
DARPP-32 events	5	6	5.5E-03
TRAF6 mediated NF-kB activation	5	7	8.5E-03
Purine salvage	5	7	7.0E-03
Activation of DNA fragmentation factor	4	5	2.0E-03
GRB2:SOS provides linkage to MAPK signaling for Intergrins	4	5	5.0E-03
Beta-oxidation of very long chain fatty acids	4	5	4.5E-03
Apoptosis induced DNA fragmentation	4	5	2.0E-03
mitochondrial fatty acid beta-oxidation of unsaturated fatty acids	4	5	5.0E-03
CREB phosphorylation	4	4	2.0E-03
Utilization of Ketone Bodies	3	3	6.0E-03
DNA Damage Reversal	3	3	5.5E-03
Activation of the AP-1 family of transcription factors	3	3	4.5E-03

Table S8. Functional enrichments of putative transcriptional target genes using Pathway Commons.

Monocytes - Pathway Commons	No. of TF
Proteoglycan syndecan-mediated signaling events	18
Regulation of CDC42 activity	15
LKB1 signaling events	14
Glypican pathway	13
Interferon Signaling	13
Sphingosine 1-phosphate (S1P) pathway	13
IL5-mediated signaling events	12
Syndecan-1-mediated signaling events	12
IL3-mediated signaling events	11
Mitotic Prophase	11
Golgi Cisternae Pericentriolar Stack Reorganization	11
Interferon alpha/beta signaling	11
IFN-gamma pathway	10
Signaling events mediated by Hepatocyte Growth Factor Receptor (c-Met)	10
Recruitment of mitotic centrosome proteins and complexes	10
GMCSF-mediated signaling events	10
CDC42 signaling events	10
Centrosome maturation	10
ATR signaling pathway	10
Thrombin/protease-activated receptor (PAR) pathway	9
Antigen processing-Cross presentation	9
ErbB receptor signaling network	9
IL23-mediated signaling events	9
VEGF and VEGFR signaling network	9
Class I MHC mediated antigen processing & presentation	9
Alpha9 beta1 integrin signaling events	9
Endothelins	9
PAR1-mediated thrombin signaling events	9
Signaling events mediated by VEGFR1 and VEGFR2	9
Integrin family cell surface interactions	9
Plasma membrane estrogen receptor signaling	9
Nectin adhesion pathway	9
Beta1 integrin cell surface interactions	9
Internalization of ErbB1	8
G2/M Transition	8
Urokinase-type plasminogen activator (uPA) and uPAR-mediated signaling	8
Loss of Nlp from mitotic centrosomes	8
Arf6 signaling events	8
EGFR-dependent Endothelin signaling events	8
ER-Phagosome pathway	8
IGF1 pathway	8
ErbB1 downstream signaling	8
Loss of proteins required for interphase microtubule organization from the centrosome	8
Arf6 trafficking events	8
Signaling events mediated by focal adhesion kinase	8
Class I PI3K signaling events mediated by Akt	8
mTOR signaling pathway	8
Insulin Pathway	8
Arf6 downstream pathway	8
PDGFR-beta signaling pathway	8
PDGF receptor signaling network	8
Glypican 1 network	8
EGF receptor (ErbB1) signaling pathway	8
Class I PI3K signaling events	8
S1P1 pathway	8
AP-1 transcription factor network	7
Mitotic G2-G2/M phases	7
Signaling events mediated by HDAC Class II	7
Immune System	7
p53 pathway	6
Direct p53 effectors	6
TRAIL signaling pathway	6
Processing of Capped Intronless Pre-mRNA	6
Post-Elongation Processing of Intronless pre-mRNA	6
TNF signaling	6
CXCR4-mediated signaling events	6
HIF-1-alpha transcription factor network	6
Processing of Intronless Pre-mRNAs	5
Caspase cascade in apoptosis	5
Trk receptor signaling mediated by the MAPK pathway	5
CD40/CD40L signaling	5
FAS (CD95) signaling pathway	5
Canonical NF-kappaB pathway	5
IL4-mediated signaling events	5
Alpha-synuclein signaling	5

CD4^{+} T cells - Pathway Commons	No. of TF
TCR signaling in naive CD8^{+} T cells	36
IL12-mediated signaling events	24
Downstream signaling in naive CD8^{+} T cells	21
TCR signaling in naive CD4^{+} T cells	21
IL12 signaling mediated by STAT4	20
Validated transcriptional targets of AP1 family members Fra1 and Fra2	19
CXCR4-mediated signaling events	17
ATF-2 transcription factor network	15
Thrombin/protease-activated receptor (PAR) pathway	14
TCR signaling	14
PAR1-mediated thrombin signaling events	14
Downstream TCR signaling	14
Internalization of ErbB1	13
Urokinase-type plasminogen activator (uPA) and uPAR-mediated signaling	13
ErbB receptor signaling network	13
Arf6 signaling events	13
VEGF and VEGFR signaling network	13
EGFR-dependent Endothelin signaling events	13
ErbB1 downstream signaling	13
Signaling events mediated by VEGFR1 and VEGFR2	13
salvage pathways of pyrimidine ribonucleotides	13
Transcription from mitochondrial promoters	13
Arf6 trafficking events	13
Signaling events mediated by focal adhesion kinase	13
Class I PI3K signaling events mediated by Akt	13
mTOR signaling pathway	13
Integrin family cell surface interactions	13
Insulin Pathway	13
Arf6 downstream pathway	13
PDGFR-beta signaling pathway	13
Glypican 1 network	13
EGF receptor (ErbB1) signaling pathway	13
Class I PI3K signaling events	13
S1P1 pathway	13
IFN-gamma pathway	12
Transcription	12
Alpha9 beta1 integrin signaling events	12
Extrinsic Pathway for Apoptosis	12
Death Receptor Signalling	12
IL5-mediated signaling events	12
CD40/CD40L signaling	12
Beta1 integrin cell surface interactions	12
Signaling events mediated by Hepatocyte Growth Factor Receptor (c-Met)	11
LKB1 signaling events	11
IGF1 pathway	11
Syndecan-2-mediated signaling events	11
GMCSF-mediated signaling events	11
PDGF receptor signaling network	11
Plasma membrane estrogen receptor signaling	11
Nectin adhesion pathway	11
Canonical NF-kappaB pathway	11
Glypican pathway	10
Processing of Intronless Pre-mRNAs	10
IL2-mediated signaling events	10
FAS (CD95) signaling pathway	10
Signaling events regulated by Ret tyrosine kinase	10
Adaptive Immune System	9
Antigen processing-Cross presentation	9
Proteoglycan syndecan-mediated signaling events	9
ER-Phagosome pathway	9
Endothelins	9
Regulation of CDC42 activity	9
RIG-I/MDA5 mediated induction of IFN-alpha/beta pathways	9
Endogenous TLR signaling	9
Syndecan-1-mediated signaling events	9
Sphingosine 1-phosphate (S1P) pathway	9
Circadian rhythm pathway	9
IL23-mediated signaling events	8
Class I MHC mediated antigen processing & presentation	8
TRAIL signaling pathway	8
IL3-mediated signaling events	8
Signaling events mediated by TCPTP	8
IL4-mediated signaling events	8
Costimulation by the CD28 family	7
AP-1 transcription factor network	7
Signaling by Wnt	7
Degradation of beta-catenin by the destruction complex	7
Cytokine Signaling in Immune system	7
Integrin-linked kinase signaling	7
FGF signaling pathway	7
Interferon Signaling	7
BMAL1:CLOCK/NPAS2 Activates Gene Expression	7
Immune System	7
Calcium signaling in the CD4^{+} TCR pathway	6
IL8-mediated signaling events	6
IL8- and CXCR2-mediated signaling events	6
Regulation of Apoptosis	6

Regulation of RAC1 activity	6
RAC1 signaling pathway	6
Circadian Clock	6
RhoA signaling pathway	6
Netrin-1 signaling	6
CDC42 signaling events	6
Immunoregulatory interactions between a Lymphoid and a non-Lymphoid cell	6
Cell junction organization	6
HIF-1-alpha transcription factor network	6
Cyclin E associated events during G1/S transition	6
Regulation of RhoA activity	6
Interferon alpha/beta signaling	6
p53-Dependent G1 DNA Damage Response	5
Vpu mediated degradation of CD4	5
Generation of second messenger molecules	5
FoxO family signaling	5
G2/M Transition	5
Autodegradation of the E3 ubiquitin ligase COP1	5
Glutamate Neurotransmitter Release Cycle	5
Cyclin A:Cdk2-associated events at S phase entry	5
Regulation of APC/C activators between G1/S and early anaphase	5
APC/C-mediated degradation of cell cycle proteins	5
Signaling by Aurora kinases	5
Cdc20:Phospho-APC/C mediated degradation of Cyclin A	5
Activation of APC/C and APC/C:Cdc20 mediated degradation of mitotic proteins	5
p53-Independent DNA Damage Response	5
p53-Independent G1/S DNA damage checkpoint	5
APC/C:Cdc20 mediated degradation of mitotic proteins	5
Cross-presentation of soluble exogenous antigens (endosomes)	5
p53-Dependent G1/S DNA damage checkpoint	5
CDT1 association with the CDC6:ORC:origin complex	5
Assembly of the pre-replicative complex	5
APC/C:Cdh1 mediated degradation of Cdc20 and other APC/C:Cdh1 targeted proteins in late mitosis/early G1	5
Regulation of ornithine decarboxylase (ODC)	5
Antigen processing: Ubiquitination & Proteasome degradation	5
Vif-mediated degradation of APOBEC3G	5
G1/S DNA Damage Checkpoints	5
Stabilization of p53	5
CDK-mediated phosphorylation and removal of Cdc6	5
Mitotic G2-G2/M phases	5
LPA receptor mediated events	5
Regulation of mitotic cell cycle	5
Ubiquitin-dependent degradation of Cyclin D1	5
SCF(Skp2)-mediated degradation of p27/p21	5
Integrins in angiogenesis	5
Signaling events mediated by HDAC Class I	5
a6b1 and a6b4 Integrin signaling	5
APC/C:Cdc20 mediated degradation of Securin	5
Ubiquitin-dependent degradation of Cyclin D	5
Calcineurin-regulated NFAT-dependent transcription in lymphocytes	5
Autodegradation of Cdh1 by Cdh1:APC/C	5
Metabolism of lipids and lipoproteins	5
Regulation of activated PAK-2p34 by proteasome mediated degradation	5
Canonical Wnt signaling pathway	5
Toll Like Receptor 9 (TLR9) Cascade	5
Destabilization of mRNA by AUF1 (hnRNP D0)	5
Regulation of nuclear beta catenin signaling and target gene transcription	5
Ubiquitin Mediated Degradation of Phosphorylated Cdc25A	5
Wnt	5
SCF-beta-TrCP mediated degradation of Emi1	5

CD20⁺ B cells - Pathway Commons	No. of TF
Interferon alpha/beta signaling	12
Alpha6Beta4Integrin	11
Validated targets of C-MYC transcriptional activation	11
IL8- and CXCR2-mediated signaling events	10
Antigen processing-Cross presentation	9
BCR signaling pathway	9
IL6-mediated signaling events	9
Cell junction organization	9
ER-Phagosome pathway	8
Regulation of CDC42 activity	8
CXCR4-mediated signaling events	8
CDC42 signaling events	8
Syndecan-4-mediated signaling events	8
Noncanonical Wnt signaling pathway	7
Class I MHC mediated antigen processing & presentation	7
Cell-Cell communication	7
TCR signaling in naive CD8⁺ T cells	6
Wnt signaling network	6
Regulation of cytoplasmic and nuclear SMAD2/3 signaling	6
Regulation of nuclear SMAD2/3 signaling	6
Interleukin-1 signaling	6
EPO signaling pathway	6
Glypican 3 network	6
TCA cycle variation III (eukaryotic)	6
IL4-mediated signaling events	6
Canonical Wnt signaling pathway	6
TGF-beta receptor signaling	6
N-cadherin signaling events	6
Trk receptor signaling mediated by PI3K and PLC-gamma	5
Notch-mediated HES/HEY network	5
Posttranslational regulation of adherens junction stability and dissassembly	5
Notch signaling pathway	5
LPA receptor mediated events	5
ATF-2 transcription factor network	5
Mitotic Prophase	5
Signaling by Interleukins	5
Golgi Cisternae Pericentriolar Stack Reorganization	5
Interferon Signaling	5
Regulation of nuclear beta catenin signaling and target gene transcription	5

Table S9. Functional enrichments of putative transcriptional target genes using WikiPathways.

Monocytes - WikiPathways	No. of TF
Calcium Regulation in the Cardiac Cell:WP536	17
TGF Beta Signaling Pathway:WP366	15
TGF Beta Signaling Pathway:WP560	15
Mitochondrial Gene Expression:WP391	13
RANKL/RANK Signaling Pathway:WP2018	11
Corticotropin-releasing hormone:WP2355	8
Diurnally regulated genes with circadian orthologs:WP410	6
Diurnally Regulated Genes with Circadian Orthologs:WP410	6
Primary Focal Segmental Glomerulosclerosis FSGS:WP2572	5
TSH signaling pathway:WP2032	5
Insulin Signaling:WP481	4
Neural Crest Differentiation:WP2064	4
Regulation of toll-like receptor signaling pathway:KEGG-:WP1449	4
Vitamin B12 Metabolism:WP1533	4
IL-4 Signaling Pathway:WP395	3
Heart Development:WP1591	3
Myometrial Relaxation and Contraction Pathways:WP289	3
MAPK Signaling Pathway:WP382	3
Histone Modifications:WP2369	3
Alpha6-Beta4 Integrin Signaling Pathway:WP244	3
Proteasome Degradation:WP183	3
MAPK signaling pathway:WP382	3
Type II interferon signaling (IFNG):WP619	3
Interferon type I signaling pathways:WP585	3
TCR Signaling Pathway:WP69	3
Cell Cycle:WP179	3
Apoptosis Modulation by HSP70:WP384	3
Cell cycle:WP179	3
Fatty Acid Beta Oxidation:WP143	3
Integrated Pancreatic Cancer Pathway:WP2377	3
Allograft Rejection:KEGG-hsa05330:WP2328	3

CD4^{+} T cells - WikiPathways	No. of TF
SIDS Susceptibility Pathways:WP706	13
TCR Signaling Pathway:WP69	12
Signaling Pathways in Glioblastoma:WP2261	11
TGF Beta Signaling Pathway:WP560	10
TGF Beta Signaling Pathway:WP366	10
MAPK Signaling Pathway:WP382	8
Alpha 6 Beta 4 signaling pathway:WP244	8
Cytoplasmic Ribosomal Proteins:WP477	7
DNA damage response (only ATM dependent):WP710	7
RANKL/RANK Signaling Pathway:WP2018	7
DNA damage response:WP707	7
MAPK signaling pathway:WP382	7
Oncostatin M Signaling Pathway:WP2374	7
Endochondral Ossification:WP474	6
Myometrial Relaxation and Contraction Pathways:WP289	6
Neural Crest Differentiation:WP2064	6
IL-5 Signaling Pathway:WP127	6
Wnt Signaling Pathway NetPath:WP363	5
estrogen signalling:WP712	5
Proteasome Degradation:WP183	5
Focal Adhesion:WP306	5
Androgen receptor signaling pathway:WP138	5
IL-7 Signaling Pathway:WP205	5
DNA Damage Response (only ATM dependent):WP710	5
Regulation of toll-like receptor signaling pathway:KEGG-:WP1449	5
IL-6 signaling pathway:WP364	5
Nifedipine Activity:WP259	4
IL-4 Signaling Pathway:WP395	4
Physiological and Pathological Hypertrophy of the Heart:WP1528	4
AGE/RAGE pathway:WP2324	4
Diurnally regulated genes with circadian orthologs:WP410	4
IL-6 Signaling Pathway:WP364	4
Pathogenic Escherichia coli infection:KEGG-hsa05130:WP2272	4
Corticotropin-releasing hormone:WP2355	4
T Cell Receptor Signaling Pathway:WP69	4
IL-9 Signaling Pathway:WP22	4
TSH signaling pathway:WP2032	4
Cell cycle:WP179	4
Diurnally Regulated Genes with Circadian Orthologs:WP410	4
Nifedipine:WP259	4
Hedgehog Signaling Pathway:WP47	3
Wnt Signaling Pathway Netpath:WP363	3
BCL-XL prevents the binding of truncated BID to BAX. This is prevented by BAD. BAD can displace tBID from BCL-XL. :WP231	3
Angiogenesis:WP1539	3
TSLP Signaling Pathway:WP2203	3
EGF/EGFR Signaling Pathway:WP437	3
Spinal Cord Injury:WP2431	3
BDNF signaling pathway:WP2380	3
Integrated Pancreatic Cancer Pathway:WP2377	3
Adipogenesis:WP236	3
miRs in Muscle Cell Differentiation:WP2012	3
miRNA Regulation of DNA Damage Response:WP1530	3
Integrated Breast Cancer Pathway:WP1984	3
Cholesterol Biosynthesis:WP197	3
Insulin Signaling:WP481	3
Alpha6-Beta4 Integrin Signaling Pathway:WP244	3
Wnt Signaling Pathway and Pluripotency:WP399	3
Estrogen signaling pathway:WP712	3
EPO Receptor Signaling:WP581	3
DNA Damage Response:WP707	3
Nucleotide GPCRs:WP80	3

CD20^{+} B cells - WikiPathways	No. of TF
Histone Modifications:WP2369	16
Alpha6-Beta4 Integrin Signaling Pathway:WP244	16
SIDS Susceptibility Pathways:WP706	15
DNA damage response (only ATM dependent):WP710	9
Wnt Signaling Pathway:WP428	9
TGF Beta Signaling Pathway:WP366	9
TGF Beta Signaling Pathway:WP560	9
DNA Damage Response (only ATM dependent):WP710	8
Focal Adhesion:WP306	8
Alpha 6 Beta 4 signaling pathway:WP244	8
miR-targeted genes in epithelium - TarBase:WP2002	6
ErbB Signaling Pathway:WP673	5
Wnt Signaling Pathway Netpath:WP363	5
Nuclear Receptors:WP170	5
IL-5 Signaling Pathway:WP127	5
IL-4 signaling Pathway:WP395	5
Adipogenesis:WP236	5
Myometrial Relaxation and Contraction Pathways:WP289	4
G13 Signaling Pathway:WP524	4
Calcium Regulation in the Cardiac Cell:WP536	4
IL-4 Signaling Pathway:WP395	3
Id Signaling Pathway:WP53	3
RANKL/RANK Signaling Pathway:WP2018	3
BCL-XL prevents the binding of truncated BID to BAX. This is prevented by BAD. BAD can displace tBID from BCL-XL. :WP231	3
Integrin-mediated Cell Adhesion:WP185	3
Mitochondrial Gene Expression:WP391	3
Selenium Pathway:WP15	3
Selenium Micronutrient Network:WP15	3
Proteasome Degradation:WP183	3
EGF/EGFR Signaling Pathway:WP437	3
T Cell Receptor Signaling Pathway:WP69	3
Integrin-mediated cell adhesion:WP185	3

Table S10. Functional enrichments of putative transcriptional target genes using KEGG.

Monocytes - KEGG	No. of TF
Systemic lupus erythematosus:KEGG-hsa05322	41
Chemokine signaling pathway:KEGG-hsa04062	27
Chagas disease (American trypanosomiasis):KEGG-hsa05142	11
Glutamatergic synapse:KEGG-hsa04724	10
Melanogenesis:KEGG-hsa04916	9
Lysosome:KEGG-hsa04142	8
Salivary secretion:KEGG-hsa04970	8
GnRH signaling pathway:KEGG-hsa04912	8
Vibrio cholerae infection:KEGG-hsa05110	8
Toxoplasmosis:KEGG-hsa05145	7
Adipocytokine signaling pathway:KEGG-hsa04920	7
Long-term potentiation:KEGG-hsa04720	6
Neurotrophin signaling pathway:KEGG-hsa04722	6
ABC transporters:KEGG-hsa02010	6
Cell adhesion molecules (CAMs):KEGG-hsa04514	6
Viral myocarditis:KEGG-hsa05416	6
Proteasome:KEGG-hsa03050	6
Autoimmune thyroid disease:KEGG-hsa05320	5
Intestinal immune network for IgA production:KEGG-hsa04672	5
Gap junction:KEGG-hsa04540	5
Graft-versus-host disease:KEGG-hsa05332	5
Calcium signaling pathway:KEGG-hsa04020	5
Allograft rejection:KEGG-hsa05330	5
RIG-I-like receptor signaling pathway:KEGG-hsa04622	5
Gastric acid secretion:KEGG-hsa04971	5
Porphyrin and chlorophyll metabolism:KEGG-hsa00860	5
Antigen processing and presentation:KEGG-hsa04612	5
Toll-like receptor signaling pathway:KEGG-hsa04620	4
Glycerolipid metabolism:KEGG-hsa00561	4
Basal transcription factors:KEGG-hsa03022	4
Type I diabetes mellitus:KEGG-hsa04940	4
MAPK signaling pathway:KEGG-hsa04010	3
Valine, leucine and isoleucine degradation:KEGG-hsa00280	3
Shigellosis:KEGG-hsa05131	3
Fructose and mannose metabolism:KEGG-hsa00051	3
Wnt signaling pathway:KEGG-hsa04310	3
Cell cycle:KEGG-hsa04110	3
Bile secretion:KEGG-hsa04976	3
Axon guidance:KEGG-hsa04360	2
Phosphatidylinositol signaling system:KEGG-hsa04070	2
Vascular smooth muscle contraction:KEGG-hsa04270	2
Melanoma:KEGG-hsa05218	2
Lysine degradation:KEGG-hsa00310	2
Fatty acid metabolism:KEGG-hsa00071	2
TGF-beta signaling pathway:KEGG-hsa04350	2
Leishmaniasis:KEGG-hsa05140	2
Nicotinate and nicotinamide metabolism:KEGG-hsa00760	2
Measles:KEGG-hsa05162	2
Rheumatoid arthritis:KEGG-hsa05323	2
Fatty acid elongation in mitochondria:KEGG-hsa00062	2
Leukocyte transendothelial migration:KEGG-hsa04670	2
Osteoclast differentiation:KEGG-hsa04380	2
Endocytosis:KEGG-hsa04144	2
Huntington's disease:KEGG-hsa05016	2
Fc gamma R-mediated phagocytosis:KEGG-hsa04666	1
beta-Alanine metabolism:KEGG-hsa00410	1
Bladder cancer:KEGG-hsa05219	1
DNA replication:KEGG-hsa03030	1
Oocyte meiosis:KEGG-hsa04114	1
Purine metabolism:KEGG-hsa00230	1
Progesterone-mediated oocyte maturation:KEGG-hsa04914	1
VEGF signaling pathway:KEGG-hsa04370	1
Glyoxylate and dicarboxylate metabolism:KEGG-hsa00630	1
Retinol metabolism:KEGG-hsa00830	1
Cytokine-cytokine receptor interaction:KEGG-hsa04060	1
Prion diseases:KEGG-hsa05020	1
Acute myeloid leukemia:KEGG-hsa05221	1
Fc epsilon RI signaling pathway:KEGG-hsa04664	1
Phagosome:KEGG-hsa04145	1
Tight junction:KEGG-hsa04530	1
Amoebiasis:KEGG-hsa05146	1
Staphylococcus aureus infection:KEGG-hsa05150	1
Asthma:KEGG-hsa05310	1
Arginine and proline metabolism:KEGG-hsa00330	1
Renal cell carcinoma:KEGG-hsa05211	1
B cell receptor signaling pathway:KEGG-hsa04662	1
Parkinson's disease:KEGG-hsa05012	1
Regulation of actin cytoskeleton:KEGG-hsa04810	1
Pyruvate metabolism:KEGG-hsa00620	1
p53 signaling pathway:KEGG-hsa04115	1
Glutathione metabolism:KEGG-hsa00480	1
Circadian rhythm - mammal:KEGG-hsa04710	1
NOD-like receptor signaling pathway:KEGG-hsa04621	1
Ribosome:KEGG-hsa03010	1
Propanoate metabolism:KEGG-hsa00640	1
mRNA surveillance pathway:KEGG-hsa03015	1
Notch signaling pathway:KEGG-hsa04330	1
Oxidative phosphorylation:KEGG-hsa00190	1
Endometrial cancer:KEGG-hsa05213	1
One carbon pool by folate:KEGG-hsa00670	1
Alzheimer's disease:KEGG-hsa05010	1
Jak-STAT signaling pathway:KEGG-hsa04630	1
Base excision repair:KEGG-hsa03410	1
Glioma:KEGG-hsa05214	1
Amyotrophic lateral sclerosis (ALS):KEGG-hsa05014	1
Adherens junction:KEGG-hsa04520	1
Olfactory transduction:KEGG-hsa04740	1
Valine, leucine and isoleucine biosynthesis:KEGG-hsa00290	1
T cell receptor signaling pathway:KEGG-hsa04660	1
Type II diabetes mellitus:KEGG-hsa04930	1
Bacterial invasion of epithelial cells:KEGG-hsa05100	1
Epithelial cell signaling in Helicobacter pylori infection:KEGG-hsa05120	1
Insulin signaling pathway:KEGG-hsa04910	1
Natural killer cell mediated cytotoxicity:KEGG-hsa04650	1

CD4+ T cells - KEGG	No. of TF
Lysosome:KEGG-hsa04142	21
Glycosaminoglycan degradation:KEGG-hsa00531	14
MAPK signaling pathway:KEGG-hsa04010	13
Shigellosis:KEGG-hsa05131	13
RIG-I-like receptor signaling pathway:KEGG-hsa04622	13
Cytosolic DNA-sensing pathway:KEGG-hsa04623	12
Chagas disease (American trypanosomiasis):KEGG-hsa05142	11
Hematopoietic cell lineage:KEGG-hsa04640	10
T cell receptor signaling pathway:KEGG-hsa04660	9
Ribosome:KEGG-hsa03010	7
Proteasome:KEGG-hsa03050	7
Pentose and glucuronate interconversions:KEGG-hsa00040	6
Jak-STAT signaling pathway:KEGG-hsa04630	6
Glycosphingolipid biosynthesis - ganglio series:KEGG-hsa00604	5
Endometrial cancer:KEGG-hsa05213	5
Bacterial invasion of epithelial cells:KEGG-hsa05100	5
Gap junction:KEGG-hsa04540	4
Measles:KEGG-hsa05162	4
p53 signaling pathway:KEGG-hsa04115	4
Circadian rhythm - mammal:KEGG-hsa04710	4
Pathogenic Escherichia coli infection:KEGG-hsa05130	4
ErbB signaling pathway:KEGG-hsa04012	4
Antigen processing and presentation:KEGG-hsa04612	4
Fc gamma R-mediated phagocytosis:KEGG-hsa04666	3
Long-term potentiation:KEGG-hsa04720	3
Valine, leucine and isoleucine degradation:KEGG-hsa00280	3
N-Glycan biosynthesis:KEGG-hsa00510	3
Collecting duct acid secretion:KEGG-hsa04966	3
Chemokine signaling pathway:KEGG-hsa04062	3
Rheumatoid arthritis:KEGG-hsa05323	3
Focal adhesion:KEGG-hsa04510	3
Alanine, aspartate and glutamate metabolism:KEGG-hsa00250	3
Chronic myeloid leukemia:KEGG-hsa05220	3
Cell cycle:KEGG-hsa04110	3
Systemic lupus erythematosus:KEGG-hsa05322	3
Steroid hormone biosynthesis:KEGG-hsa00140	3
Glutamatergic synapse:KEGG-hsa04724	3
Small cell lung cancer:KEGG-hsa05222	3
Phosphatidylinositol signaling system:KEGG-hsa04070	2
Neurotrophin signaling pathway:KEGG-hsa04722	2
DNA replication:KEGG-hsa03030	2
Non-small cell lung cancer:KEGG-hsa05223	2
Purine metabolism:KEGG-hsa00230	2
Neuroactive ligand-receptor interaction:KEGG-hsa04080	2
Melanoma:KEGG-hsa05218	2
Pathways in cancer:KEGG-hsa05200	2
Nucleotide excision repair:KEGG-hsa03420	2
Inositol phosphate metabolism:KEGG-hsa00562	2
Arrhythmogenic right ventricular cardiomyopathy (ARVC):KEGG-hsa05412	2
Acute myeloid leukemia:KEGG-hsa05221	2
Toll-like receptor signaling pathway:KEGG-hsa04620	2
Pyruvate metabolism:KEGG-hsa00620	2
Regulation of actin cytoskeleton:KEGG-hsa04810	2
Adipocytokine signaling pathway:KEGG-hsa04920	2
Fatty acid elongation in mitochondria:KEGG-hsa00062	2
Alzheimer's disease:KEGG-hsa05010	2
Adherens junction:KEGG-hsa04520	2
Huntington's disease:KEGG-hsa05016	2
Axon guidance:KEGG-hsa04360	1
Autoimmune thyroid disease:KEGG-hsa05320	1
Pentose phosphate pathway:KEGG-hsa00030	1

RNA degradation:KEGG-hsa03018	1
Selenocompound metabolism:KEGG-hsa00450	1
SNARE interactions in vesicular transport:KEGG-hsa04130	1
Toxoplasmosis:KEGG-hsa05145	1
Dorso-ventral axis formation:KEGG-hsa04320	1
Folate biosynthesis:KEGG-hsa00790	1
Glyoxylate and dicarboxylate metabolism:KEGG-hsa00630	1
Intestinal immune network for IgA production:KEGG-hsa04672	1
Fatty acid metabolism:KEGG-hsa00071	1
alpha-Linolenic acid metabolism:KEGG-hsa00592	1
Pancreatic cancer:KEGG-hsa05212	1
Phagosome:KEGG-hsa04145	1
Aldosterone-regulated sodium reabsorption:KEGG-hsa04960	1
TGF-beta signaling pathway:KEGG-hsa04350	1
Viral myocarditis:KEGG-hsa05416	1
Staphylococcus aureus infection:KEGG-hsa05150	1
Wnt signaling pathway:KEGG-hsa04310	1
Asthma:KEGG-hsa05310	1
Arachidonic acid metabolism:KEGG-hsa00590	1
Arginine and proline metabolism:KEGG-hsa00330	1
B cell receptor signaling pathway:KEGG-hsa04662	1
Allograft rejection:KEGG-hsa05330	1
Carbohydrate digestion and absorption:KEGG-hsa04973	1
Glycerolipid metabolism:KEGG-hsa00561	1
Basal transcription factors:KEGG-hsa03022	1
Cysteine and methionine metabolism:KEGG-hsa00270	1
NOD-like receptor signaling pathway:KEGG-hsa04621	1
Vibrio cholerae infection:KEGG-hsa05110	1
Long-term depression:KEGG-hsa04730	1
Spliceosome:KEGG-hsa03040	1
Porphyrin and chlorophyll metabolism:KEGG-hsa00860	1
Osteoclast differentiation:KEGG-hsa04380	1
Type I diabetes mellitus:KEGG-hsa04940	1
Amyotrophic lateral sclerosis (ALS):KEGG-hsa05014	1
Endocytosis:KEGG-hsa04144	1
Aminoacyl-tRNA biosynthesis:KEGG-hsa00970	1
Tryptophan metabolism:KEGG-hsa00380	1
Valine, leucine and isoleucine biosynthesis:KEGG-hsa00290	1
Cardiac muscle contraction:KEGG-hsa04260	1
Type II diabetes mellitus:KEGG-hsa04930	1
Epithelial cell signaling in Helicobacter pylori infection:KEGG-hsa05120	1
Primary immunodeficiency:KEGG-hsa05340	1
Prostate cancer:KEGG-hsa05215	1
Natural killer cell mediated cytotoxicity:KEGG-hsa04650	1

CD20$^+$ B cells - KEGG	No. of TF
Systemic lupus erythematosus:KEGG-hsa05322	27
Antigen processing and presentation:KEGG-hsa04612	14
Toxoplasmosis:KEGG-hsa05145	10
Focal adhesion:KEGG-hsa04510	10
Lysosome:KEGG-hsa04142	9
Cell adhesion molecules (CAMs):KEGG-hsa04514	9
Viral myocarditis:KEGG-hsa05416	9
Rheumatoid arthritis:KEGG-hsa05323	9
B cell receptor signaling pathway:KEGG-hsa04662	9
Type I diabetes mellitus:KEGG-hsa04940	8
Intestinal immune network for IgA production:KEGG-hsa04672	7
Graft-versus-host disease:KEGG-hsa05332	7
Fc epsilon RI signaling pathway:KEGG-hsa04664	7
Leishmaniasis:KEGG-hsa05140	7
Proteasome:KEGG-hsa03050	7
Adipocytokine signaling pathway:KEGG-hsa04920	7
T cell receptor signaling pathway:KEGG-hsa04660	7
Type II diabetes mellitus:KEGG-hsa04930	7
Autoimmune thyroid disease:KEGG-hsa05320	6
Phagosome:KEGG-hsa04145	6
Allograft rejection:KEGG-hsa05330	6
Neurotrophin signaling pathway:KEGG-hsa04722	5
Chemokine signaling pathway:KEGG-hsa04062	5
Neuroactive ligand-receptor interaction:KEGG-hsa04080	4
Staphylococcus aureus infection:KEGG-hsa05150	4
Asthma:KEGG-hsa05310	4
Pyruvate metabolism:KEGG-hsa00620	4
p53 signaling pathway:KEGG-hsa04115	4
Spliceosome:KEGG-hsa03040	4
Gastric acid secretion:KEGG-hsa04971	4
mTOR signaling pathway:KEGG-hsa04150	3
VEGF signaling pathway:KEGG-hsa04370	3
MAPK signaling pathway:KEGG-hsa04010	3
Cytokine-cytokine receptor interaction:KEGG-hsa04060	3
Cysteine and methionine metabolism:KEGG-hsa00270	3
RIG-I-like receptor signaling pathway:KEGG-hsa04622	3
Glycerophospholipid metabolism:KEGG-hsa00564	3
ECM-receptor interaction:KEGG-hsa04512	3
Glycosphingolipid biosynthesis - ganglio series:KEGG-hsa00604	2
Vascular smooth muscle contraction:KEGG-hsa04270	2
Glycolysis / Gluconeogenesis:KEGG-hsa00010	2
Citrate cycle (TCA cycle):KEGG-hsa00020	2
Valine, leucine and isoleucine degradation:KEGG-hsa00280	2
Pancreatic cancer:KEGG-hsa05212	2
Tuberculosis:KEGG-hsa05152	2
GnRH signaling pathway:KEGG-hsa04912	2
Measles:KEGG-hsa05162	2
Renal cell carcinoma:KEGG-hsa05211	2
Basal transcription factors:KEGG-hsa03022	2
Pentose and glucuronate interconversions:KEGG-hsa00040	2
Osteoclast differentiation:KEGG-hsa04380	2
Jak-STAT signaling pathway:KEGG-hsa04630	2
Glutamatergic synapse:KEGG-hsa04724	2
Endocytosis:KEGG-hsa04144	2
Epithelial cell signaling in Helicobacter pylori infection:KEGG-hsa05120	2
Small cell lung cancer:KEGG-hsa05222	2
Fc gamma R-mediated phagocytosis:KEGG-hsa04666	1
Long-term potentiation:KEGG-hsa04720	1
RNA degradation:KEGG-hsa03018	1
Bladder cancer:KEGG-hsa05219	1
Selenocompound metabolism:KEGG-hsa00450	1
Histidine metabolism:KEGG-hsa00340	1
Dorso-ventral axis formation:KEGG-hsa04320	1
Melanoma:KEGG-hsa05218	1
N-Glycan biosynthesis:KEGG-hsa00510	1
Retinol metabolism:KEGG-hsa00830	1
Gap junction:KEGG-hsa04540	1
Arrhythmogenic right ventricular cardiomyopathy (ARVC):KEGG-hsa05412	1
Acute myeloid leukemia:KEGG-hsa05221	1
Calcium signaling pathway:KEGG-hsa04020	1
Shigellosis:KEGG-hsa05131	1
Glycosylphosphatidylinositol(GPI)-anchor biosynthesis:KEGG-hsa00563	1
Amoebiasis:KEGG-hsa05146	1
Wnt signaling pathway:KEGG-hsa04310	1
Hepatitis C:KEGG-hsa05160	1
Arachidonic acid metabolism:KEGG-hsa00590	1
Sulfur metabolism:KEGG-hsa00920	1
Parkinson's disease:KEGG-hsa05012	1
Regulation of actin cytoskeleton:KEGG-hsa04810	1
Circadian rhythm - mammal:KEGG-hsa04710	1
Vibrio cholerae infection:KEGG-hsa05110	1
Colorectal cancer:KEGG-hsa05210	1
Cytosolic DNA-sensing pathway:KEGG-hsa04623	1
Fatty acid elongation in mitochondria:KEGG-hsa00062	1
ErbB signaling pathway:KEGG-hsa04012	1
Bile secretion:KEGG-hsa04976	1
Mismatch repair:KEGG-hsa03430	1
Amyotrophic lateral sclerosis (ALS):KEGG-hsa05014	1
Pyrimidine metabolism:KEGG-hsa00240	1
Taste transduction:KEGG-hsa04742	1
Insulin signaling pathway:KEGG-hsa04910	1
Prostate cancer:KEGG-hsa05215	1
Natural killer cell mediated cytotoxicity:KEGG-hsa04650	1

Table S11. Functional enrichments of putative transcriptional target genes using Gene Ontology (GO).

ID	Monocytes - Gene Ontology (GO)	No. of TF
GO:0000786	nucleosome	58
GO:0006334	nucleosome assembly	49
GO:0005694	chromosome	48
GO:0007044	cell-substrate junction assembly	29
GO:0009887	organ morphogenesis	26
GO:0006937	regulation of muscle contraction	21
GO:0010628	positive regulation of gene expression	20
GO:0033177	proton-transporting two-sector ATPase complex, proton-transporting domain	17
GO:0009409	response to cold	17
GO:0019219	regulation of nucleobase, nucleoside, nucleotide and nucleic acid metabolic process	16
GO:0030496	midbody	16
GO:0009653	anatomical structure morphogenesis	14
GO:0048513	organ development	14
GO:0005929	cilium	14
GO:0008277	regulation of G-protein coupled receptor protein signaling pathway	13
GO:0003779	actin binding	13
GO:0044057	regulation of system process	12
GO:0071203	WASH complex	12
GO:0007268	synaptic transmission	11
GO:0008308	voltage-gated anion channel activity	11
GO:0043190	ATP-binding cassette (ABC) transporter complex	10
GO:0016607	nuclear speck	10
GO:0033363	secretory granule organization	10
GO:0033572	transferrin transport	10
GO:0001843	neural tube closure	10
GO:0051117	ATPase binding	9
GO:0034220	ion transmembrane transport	9
GO:0030036	actin cytoskeleton organization	9
GO:0034728	nucleosome organization	9
GO:0044419	interspecies interaction between organisms	9
GO:0048598	embryonic morphogenesis	8
GO:0003924	GTPase activity	8
GO:0005096	GTPase activator activity	8
GO:2000112	regulation of cellular macromolecule biosynthetic process	8
GO:0042288	MHC class I protein binding	8
GO:0070851	growth factor receptor binding	8
GO:0016509	long-chain-3-hydroxyacyl-CoA dehydrogenase activity	8
GO:0006325	chromatin organization	8
GO:0034622	cellular macromolecular complex assembly	8
GO:0001525	angiogenesis	7
GO:0030168	platelet activation	7
GO:0008284	positive regulation of cell proliferation	7
GO:0002699	positive regulation of immune effector process	7
GO:0008307	structural constituent of muscle	7
GO:0042605	peptide antigen binding	7
GO:0004703	G-protein coupled receptor kinase activity	7
GO:0070003	threonine-type peptidase activity	7
GO:0010740	positive regulation of intracellular protein kinase cascade	7
GO:0019882	antigen processing and presentation	7
GO:0045202	synapse	7
GO:0030029	actin filament-based process	7
GO:0032496	response to lipopolysaccharide	7
GO:0005839	proteasome core complex	7
GO:0044421	extracellular region part	7
GO:0043130	ubiquitin binding	7
GO:0006695	cholesterol biosynthetic process	7
GO:0008285	negative regulation of cell proliferation	7
GO:0045211	postsynaptic membrane	7
GO:0031122	cytoplasmic microtubule organization	6
GO:0090257	regulation of muscle system process	6
GO:0019904	protein domain specific binding	6
GO:0042127	regulation of cell proliferation	6
GO:0031668	cellular response to extracellular stimulus	6
GO:0030017	sarcomere	6
GO:0044452	nucleolar part	6
GO:0061041	regulation of wound healing	6
GO:0005576	extracellular region	6
GO:0005925	focal adhesion	6
GO:0019222	regulation of metabolic process	6
GO:0030099	myeloid cell differentiation	6
GO:0045121	membrane raft	6
GO:0006955	immune response	6
GO:0071844	cellular component assembly at cellular level	6
GO:0006595	polyamine metabolic process	6
GO:0010468	regulation of gene expression	6
GO:0019901	protein kinase binding	6
GO:0007186	G-protein coupled receptor protein signaling pathway	6
GO:0005126	cytokine receptor binding	6
GO:0016604	nuclear body	6
GO:0042742	defense response to bacterium	6
GO:0007569	cell aging	6
GO:0048731	system development	6
GO:0033762	response to glucagon stimulus	6
GO:0010627	regulation of intracellular protein kinase cascade	6
GO:0051220	cytoplasmic sequestering of protein	5
GO:0006821	chloride transport	5
GO:0048858	cell projection morphogenesis	5
GO:0002474	antigen processing and presentation of peptide antigen via MHC class I	5
GO:0043085	positive regulation of catalytic activity	5
GO:0007507	heart development	5
GO:0007213	muscarinic acetylcholine receptor signaling pathway	5
GO:0007015	actin filament organization	5
GO:0006812	cation transport	5
GO:0003002	regionalization	5
GO:0051051	negative regulation of transport	5
GO:0007067	mitosis	5
GO:0031490	chromatin DNA binding	5
GO:0005813	centrosome	5
GO:0021549	cerebellum development	5
GO:0044437	vacuolar part	5
GO:0009581	detection of external stimulus	5
GO:0060341	regulation of cellular localization	5
GO:0019898	extrinsic to membrane	5
GO:0005815	microtubule organizing center	5
GO:0032993	protein-DNA complex	5
GO:0004674	protein serine/threonine kinase activity	5
GO:0045735	nutrient reservoir activity	5
GO:0016477	cell migration	5
GO:0071260	cellular response to mechanical stimulus	5
GO:0004857	enzyme inhibitor activity	5
GO:0009952	anterior/posterior pattern formation	5
GO:0007155	cell adhesion	5
GO:0032269	negative regulation of cellular protein metabolic process	5
GO:0006820	anion transport	5
GO:0006417	regulation of translation	5
GO:0001906	cell killing	5
GO:0006935	chemotaxis	5
GO:0035145	exon-exon junction complex	5
GO:0001819	positive regulation of cytokine production	5
GO:0043011	myeloid dendritic cell differentiation	5
GO:0009636	response to toxin	5
GO:0051056	regulation of small GTPase mediated signal transduction	5
GO:0003682	chromatin binding	5
GO:0003714	transcription corepressor activity	5
GO:0002697	regulation of immune effector process	5
GO:0008138	protein tyrosine/serine/threonine phosphatase activity	5

ID	$CD4^+$ T cells - Gene Ontology (GO)	No. of TF
GO:0034097	response to cytokine stimulus	24
GO:0003700	sequence-specific DNA binding transcription factor activity	19
GO:0006687	glycosphingolipid metabolic process	16
GO:0016568	chromatin modification	15
GO:0045851	pH reduction	15
GO:0006464	protein modification process	14
GO:0045667	regulation of osteoblast differentiation	14
GO:0034381	plasma lipoprotein particle clearance	14
GO:0048009	insulin-like growth factor receptor signaling pathway	13
GO:0035085	cilium axoneme	13
GO:0007040	lysosome organization	13
GO:0001707	mesoderm formation	13
GO:0051117	ATPase binding	12
GO:0006596	polyamine biosynthetic process	12
GO:0043565	sequence-specific DNA binding	12
GO:0006390	transcription from mitochondrial promoter	12
GO:2000026	regulation of multicellular organismal development	12
GO:0043536	positive regulation of blood vessel endothelial cell migration	12
GO:0010717	regulation of epithelial to mesenchymal transition	12
GO:0008285	negative regulation of cell proliferation	12
GO:0005672	transcription factor TFIIA complex	11
GO:0030511	positive regulation of transforming growth factor beta receptor signaling pathway	11
GO:0005765	lysosomal membrane	11
GO:0030140	trans-Golgi network transport vesicle	11
GO:0017119	Golgi transport complex	11
GO:0050852	T cell receptor signaling pathway	11
GO:0071277	cellular response to calcium ion	11
GO:0042277	peptide binding	10
GO:0006289	nucleotide-excision repair	10
GO:0031464	Cul4A-RING ubiquitin ligase complex	10
GO:0051592	response to calcium ion	10
GO:0050885	neuromuscular process controlling balance	10
GO:0071496	cellular response to external stimulus	10
GO:0051591	response to cAMP	10
GO:0042383	sarcolemma	10
GO:0000315	organellar large ribosomal subunit	10

GO:0080090	regulation of primary metabolic process	9
GO:0006952	defense response	9
GO:0022415	viral reproductive process	9
GO:0019904	protein domain specific binding	9
GO:0090398	cellular senescence	9
GO:0031294	lymphocyte costimulation	9
GO:0070003	threonine-type peptidase activity	9
GO:0006414	translational elongation	9
GO:0016018	cyclosporin A binding	9
GO:0031331	positive regulation of cellular catabolic process	9
GO:0005839	proteasome core complex	9
GO:0006357	regulation of transcription from RNA polymerase II promoter	9
GO:0071565	nBAF complex	9
GO:0006338	chromatin remodeling	9
GO:0045597	positive regulation of cell differentiation	9
GO:0050708	regulation of protein secretion	9
GO:0003677	DNA binding	8
GO:0060021	palate development	8
GO:0008284	positive regulation of cell proliferation	8
GO:0045595	regulation of cell differentiation	8
GO:0001817	regulation of cytokine production	8
GO:0001649	osteoblast differentiation	8
GO:0032813	tumor necrosis factor receptor superfamily binding	8
GO:0003690	double-stranded DNA binding	8
GO:0018279	protein N-linked glycosylation via asparagine	8
GO:0006383	transcription from RNA polymerase III promoter	8
GO:0003779	actin binding	8
GO:0051240	positive regulation of multicellular organismal process	8
GO:0042752	regulation of circadian rhythm	8
GO:0030530	heterogeneous nuclear ribonucleoprotein complex	8
GO:0004861	cyclin-dependent protein kinase inhibitor activity	8
GO:0030203	glycosaminoglycan metabolic process	8
GO:0048821	erythrocyte development	8
GO:0051899	membrane depolarization	8
GO:0003714	transcription corepressor activity	8
GO:0022627	cytosolic small ribosomal subunit	8
GO:0008105	asymmetric protein localization	8
GO:0006415	translational termination	8
GO:0009897	external side of plasma membrane	7
GO:0048015	phosphatidylinositol-mediated signaling	7
GO:0045668	negative regulation of osteoblast differentiation	7
GO:0019083	viral transcription	7
GO:0060255	regulation of macromolecule metabolic process	7
GO:0045087	innate immune response	7
GO:0007507	heart development	7
GO:0031018	endocrine pancreas development	7
GO:0042127	regulation of cell proliferation	7
GO:0031371	ubiquitin conjugating enzyme complex	7
GO:0019843	rRNA binding	7
GO:0050863	regulation of T cell activation	7
GO:0006879	cellular iron ion homeostasis	7
GO:0002576	platelet degranulation	7
GO:0006385	transcription elongation from RNA polymerase III promoter	7
GO:0072395	signal transduction involved in cell cycle checkpoint	7
GO:0005178	integrin binding	7
GO:0006521	regulation of cellular amino acid metabolic process	7
GO:0006955	immune response	7
GO:0051302	regulation of cell division	7
GO:0009952	anterior/posterior pattern formation	7

GO:0042274	ribosomal small subunit biogenesis	7
GO:0007050	cell cycle arrest	7
GO:0033572	transferrin transport	7
GO:0071445	cellular response to protein stimulus	7
GO:0006386	termination of RNA polymerase III transcription	7
GO:0045599	negative regulation of fat cell differentiation	7
GO:0019901	protein kinase binding	7
GO:0031625	ubiquitin protein ligase binding	7
GO:0045682	regulation of epidermis development	7
GO:0043687	post-translational protein modification	7
GO:0005544	calcium-dependent phospholipid binding	7
GO:0002521	leukocyte differentiation	7
GO:0030168	platelet activation	6
GO:0016331	morphogenesis of embryonic epithelium	6
GO:0000184	nuclear-transcribed mRNA catabolic process, nonsense-mediated decay	6
GO:0007623	circadian rhythm	6
GO:0045111	intermediate filament cytoskeleton	6
GO:0006954	inflammatory response	6
GO:0030154	cell differentiation	6
GO:0006366	transcription from RNA polymerase II promoter	6
GO:0032480	negative regulation of type I interferon production	6
GO:0014070	response to organic cyclic compound	6
GO:0000502	proteasome complex	6
GO:0042763	intracellular immature spore	6
GO:0005068	transmembrane receptor protein tyrosine kinase adaptor activity	6
GO:0007275	multicellular organismal development	6
GO:0008277	regulation of G-protein coupled receptor protein signaling pathway	6
GO:0019894	kinesin binding	6
GO:0030518	steroid hormone receptor signaling pathway	6
GO:0000287	magnesium ion binding	6
GO:0002696	positive regulation of leukocyte activation	6
GO:0048589	developmental growth	6
GO:0032479	regulation of type I interferon production	6
GO:0016234	inclusion body	6
GO:0010557	positive regulation of macromolecule biosynthetic process	6
GO:0031060	regulation of histone methylation	6
GO:0009611	response to wounding	6
GO:0006665	sphingolipid metabolic process	6
GO:0031123	RNA 3'-end processing	6
GO:0006937	regulation of muscle contraction	6
GO:0032269	negative regulation of cellular protein metabolic process	6
GO:0045669	positive regulation of osteoblast differentiation	6
GO:0030879	mammary gland development	6
GO:0070491	repressing transcription factor binding	6
GO:0002040	sprouting angiogenesis	6
GO:0004175	endopeptidase activity	6
GO:0006631	fatty acid metabolic process	6
GO:0001657	ureteric bud development	6
GO:0002076	osteoblast development	6
GO:0009358	polyphosphate kinase complex	6
GO:0015929	hexosaminidase activity	6
GO:0044419	interspecies interaction between organisms	6
GO:0048384	retinoic acid receptor signaling pathway	6
GO:0007565	female pregnancy	6
GO:0016791	phosphatase activity	6
GO:0051952	regulation of amine transport	6
GO:0006875	cellular metal ion homeostasis	6
GO:0017124	SH3 domain binding	5
GO:0033209	tumor necrosis factor-mediated signaling pathway	5

GO:0008234	cysteine-type peptidase activity	5
GO:0006090	pyruvate metabolic process	5
GO:0032526	response to retinoic acid	5
GO:0045787	positive regulation of cell cycle	5
GO:0045075	regulation of interleukin-12 biosynthetic process	5
GO:0006605	protein targeting	5
GO:0005798	Golgi-associated vesicle	5
GO:0051437	positive regulation of ubiquitin-protein ligase activity involved in mitotic cell cycle	5
GO:0090263	positive regulation of canonical Wnt receptor signaling pathway	5
GO:0030522	intracellular receptor mediated signaling pathway	5
GO:0005743	mitochondrial inner membrane	5
GO:0051436	negative regulation of ubiquitin-protein ligase activity involved in mitotic cell cycle	5
GO:0000245	spliceosome assembly	5
GO:0000188	inactivation of MAPK activity	5
GO:0045637	regulation of myeloid cell differentiation	5
GO:0016525	negative regulation of angiogenesis	5
GO:0009409	response to cold	5
GO:0021549	cerebellum development	5
GO:0002682	regulation of immune system process	5
GO:0045740	positive regulation of DNA replication	5
GO:0050870	positive regulation of T cell activation	5
GO:0030424	axon	5
GO:0005773	vacuole	5
GO:0006897	endocytosis	5
GO:0042110	T cell activation	5
GO:0000079	regulation of cyclin-dependent protein kinase activity	5
GO:0019866	organelle inner membrane	5
GO:0051928	positive regulation of calcium ion transport	5
GO:0051781	positive regulation of cell division	5
GO:0030286	dynein complex	5
GO:0048535	lymph node development	5
GO:0071260	cellular response to mechanical stimulus	5
GO:0090092	regulation of transmembrane receptor protein serine/threonine kinase signaling pathway	5
GO:0048545	response to steroid hormone stimulus	5
GO:0033549	MAP kinase phosphatase activity	5
GO:0001963	synaptic transmission, dopaminergic	5
GO:0016829	lyase activity	5
GO:0006928	cellular component movement	5
GO:0071248	cellular response to metal ion	5
GO:0009898	internal side of plasma membrane	5
GO:0005874	microtubule	5
GO:0072379	ER membrane insertion complex	5
GO:0015629	actin cytoskeleton	5
GO:0000226	microtubule cytoskeleton organization	5
GO:0035145	exon-exon junction complex	5
GO:0030217	T cell differentiation	5
GO:0017048	Rho GTPase binding	5
GO:0030278	regulation of ossification	5
GO:0019883	antigen processing and presentation of endogenous antigen	5
GO:0046332	SMAD binding	5
GO:0001503	ossification	5
GO:0007188	G-protein signaling, coupled to cAMP nucleotide second messenger	5
GO:0051094	positive regulation of developmental process	5
GO:0032355	response to estradiol stimulus	5
GO:0032844	regulation of homeostatic process	5
GO:0042287	MHC protein binding	5

GO:0051130	positive regulation of cellular component organization	5
GO:0004930	G-protein coupled receptor activity	5

ID	CD20^{+} B cells - Gene Ontology (GO)	No. of TF
GO:0000786	nucleosome	28
GO:0005694	chromosome	25
GO:0006334	nucleosome assembly	22
GO:0042994	cytoplasmic sequestering of transcription factor	20
GO:0014070	response to organic cyclic compound	13
GO:0000122	negative regulation of transcription from RNA polymerase II promoter	13
GO:0019882	antigen processing and presentation	13
GO:0045202	synapse	13
GO:0000226	microtubule cytoskeleton organization	13
GO:0001776	leukocyte homeostasis	11
GO:0019221	cytokine-mediated signaling pathway	11
GO:0045934	negative regulation of nucleobase, nucleoside, nucleotide and nucleic acid metabolic process	11
GO:0000785	chromatin	11
GO:0009653	anatomical structure morphogenesis	10
GO:0090398	cellular senescence	10
GO:0002460	adaptive immune response based on somatic recombination of immune receptors built from immunoglobulin superfamily domains	10
GO:0033572	transferrin transport	10
GO:0030530	heterogeneous nuclear ribonucleoprotein complex	10
GO:0003714	transcription corepressor activity	10
GO:0031175	neuron projection development	10
GO:0071108	protein K48-linked deubiquitination	9
GO:0090257	regulation of muscle system process	9
GO:0051117	ATPase binding	9
GO:0070003	threonine-type peptidase activity	9
GO:0007265	Ras protein signal transduction	9
GO:0001909	leukocyte mediated cytotoxicity	9
GO:0044437	vacuolar part	9
GO:0043473	pigmentation	9
GO:0050679	positive regulation of epithelial cell proliferation	9
GO:0005839	proteasome core complex	9
GO:0005874	microtubule	9
GO:0005765	lysosomal membrane	9
GO:0007569	cell aging	9
GO:0050817	coagulation	9
GO:0045892	negative regulation of transcription, DNA-dependent	8
GO:0008134	transcription factor binding	8
GO:0000502	proteasome complex	8
GO:0051704	multi-organism process	8
GO:0045124	regulation of bone resorption	8
GO:0031418	L-ascorbic acid binding	8
GO:0071900	regulation of protein serine/threonine kinase activity	8
GO:0030097	hemopoiesis	8
GO:0009309	amine biosynthetic process	8
GO:0001819	positive regulation of cytokine production	8
GO:0008285	negative regulation of cell proliferation	8
GO:0002920	regulation of humoral immune response	7
GO:0045598	regulation of fat cell differentiation	7
GO:0002252	immune effector process	7
GO:0006959	humoral immune response	7
GO:0003924	GTPase activity	7
GO:0007389	pattern specification process	7
GO:0009890	negative regulation of biosynthetic process	7
GO:0008624	induction of apoptosis by extracellular signals	7
GO:0010557	positive regulation of macromolecule biosynthetic process	7
GO:0034968	histone lysine methylation	7
GO:0005254	chloride channel activity	7
GO:0006595	polyamine metabolic process	7
GO:0048146	positive regulation of fibroblast proliferation	7
GO:0016363	nuclear matrix	7
GO:0030278	regulation of ossification	7
GO:0005126	cytokine receptor binding	7
GO:0042613	MHC class II protein complex	7
GO:0002763	positive regulation of myeloid leukocyte differentiation	6
GO:0031294	lymphocyte costimulation	6
GO:0005643	nuclear pore	6
GO:0031228	intrinsic to Golgi membrane	6
GO:0005667	transcription factor complex	6
GO:0006385	transcription elongation from RNA polymerase III promoter	6
GO:0003779	actin binding	6
GO:0003700	sequence-specific DNA binding transcription factor activity	6
GO:0006461	protein complex assembly	6
GO:0000981	sequence-specific DNA binding RNA polymerase II transcription factor activity	6
GO:0050730	regulation of peptidyl-tyrosine phosphorylation	6
GO:0044255	cellular lipid metabolic process	6
GO:0035023	regulation of Rho protein signal transduction	6
GO:0042274	ribosomal small subunit biogenesis	6
GO:0010629	negative regulation of gene expression	6
GO:0048545	response to steroid hormone stimulus	6
GO:0006357	regulation of transcription from RNA polymerase II promoter	6
GO:0006386	termination of RNA polymerase III transcription	6
GO:0070688	MLL5-L complex	6
GO:0022618	ribonucleoprotein complex assembly	6
GO:0030325	adrenal gland development	6
GO:0008201	heparin binding	6
GO:0005769	early endosome	5
GO:0048598	embryonic morphogenesis	5
GO:0051084	'de novo' posttranslational protein folding	5
GO:0009226	nucleotide-sugar biosynthetic process	5
GO:0000184	nuclear-transcribed mRNA catabolic process, nonsense-mediated decay	5
GO:0045944	positive regulation of transcription from RNA polymerase II promoter	5
GO:0007044	cell-substrate junction assembly	5
GO:0001709	cell fate determination	5
GO:0001671	ATPase activator activity	5
GO:0045595	regulation of cell differentiation	5
GO:0034765	regulation of ion transmembrane transport	5
GO:0042605	peptide antigen binding	5
GO:0045765	regulation of angiogenesis	5
GO:0050794	regulation of cellular process	5
GO:0042127	regulation of cell proliferation	5
GO:0003690	double-stranded DNA binding	5
GO:0043565	sequence-specific DNA binding	5
GO:0002637	regulation of immunoglobulin production	5
GO:0005743	mitochondrial inner membrane	5
GO:0003707	steroid hormone receptor activity	5
GO:0030865	cortical cytoskeleton organization	5
GO:0043623	cellular protein complex assembly	5
GO:0004177	aminopeptidase activity	5
GO:0022407	regulation of cell-cell adhesion	5
GO:0030036	actin cytoskeleton organization	5
GO:0044449	contractile fiber part	5
GO:0048365	Rac GTPase binding	5
GO:0005178	integrin binding	5
GO:0016234	inclusion body	5
GO:2000026	regulation of multicellular organismal development	5
GO:0001664	G-protein-coupled receptor binding	5
GO:0034707	chloride channel complex	5
GO:0008415	acyltransferase activity	5
GO:0022891	substrate-specific transmembrane transporter activity	5
GO:0016568	chromatin modification	5
GO:0030286	dynein complex	5
GO:0006955	immune response	5
GO:0045735	nutrient reservoir activity	5
GO:0016477	cell migration	5
GO:0006937	regulation of muscle contraction	5
GO:0031012	extracellular matrix	5
GO:0043123	positive regulation of I-kappaB kinase/NF-kappaB cascade	5
GO:0004861	cyclin-dependent protein kinase inhibitor activity	5
GO:0006935	chemotaxis	5
GO:0005097	Rab GTPase activator activity	5
GO:0009636	response to toxin	5
GO:0034708	methyltransferase complex	5
GO:0003682	chromatin binding	5
GO:0042287	MHC protein binding	5
GO:0000186	activation of MAPKK activity	5
GO:0042391	regulation of membrane potential	5
GO:0051258	protein polymerization	5
GO:0017053	transcriptional repressor complex	5
GO:0010627	regulation of intracellular protein kinase cascade	5

Table S12. Total number, mean and median of transcriptional target genes.

Table S12. Total number, mean and median of putative transcriptional target genes

	Monocyte			CD4⁺ T cell			CD20⁺ B cell		
Region	Total number	Mean	Median	Total number	Mean	Median	Total number	Mean	Median
Promoter	412,572	124	24	598,570	164	33	460,035	144	24
Association rule 1	689,902	177	55	853,906	217	58	657,500	175	37
Association rule 2	928,612	234	87	1,058,387	267	80	821,343	214	53
Association rule 3	1,038,723	262	94	1,230,321	310	90	945,600	245	58
Association rule 4	639,038	164	53	771,437	197	54	596,900	160	35

	HUVEC			IMR90			MCF-7		
Region	Total number	Mean	Median	Total number	Mean	Median	Total number	Mean	Median
Promoter	313,399	148	24	197,616	108	17	195,048	105	17
Association rule 1	518,192	218	56	386,167	167	37	319,436	140	30
Association rule 2	690,909	288	90	557,979	235	62	440,468	187	48
Association rule 3	771,177	321	97	618,564	260	67	500,539	211	53
Association rule 4	480,580	202	54	362,007	157	36	295,501	130	29

	HMEC			H1-hESC			iPSC		
Region	Total number	Mean	Median	Total number	Mean	Median	Total number	Mean	Median
Promoter	222,483	115	20	276,845	140	21	233,805	129	18
Association rule 1	413,527	177	40	410,791	180	36	349,314	155	30
Association rule 2	594,464	249	67	538,213	229	55	464,210	198	47
Association rule 3	660,287	276	72	613,809	260	61	516,769	220	51
Association rule 4	385,291	165	38	376,089	165	35	320,376	143	28

Table S13. Normalized number of unique functional enrichments of transcriptional target genes predicted using promoter and extended regions for enhancer-promoter association.

Normalized number of unique functional enrichments of transcriptional target genes predicted using promoter and extended regions for enhancer-promoter association (EPA)

	KEGG	CTD Ontology	GO Slim	GO	Pathway Commons	Domains	Wiki Pathways
**Monocyte**							
Promoter	2.52	2.84	0.61	29.74	7.78	10.16	2.04
Association rule 1	2.04	2.81	0.26	26.68	5.28	9.25	1.99
Association rule 2	1.42	2.46	0.17	20.98	3.77	6.96	1.53
Association rule 3	1.28	1.90	0.12	18.98	3.38	6.94	1.37
Association rule 4	2.16	3.07	0.25	28.72	5.09	9.64	2.07
**CD4⁺ T cell**							
Promoter	1.75	2.44	0.38	27.63	6.93	9.77	2.22
Association rule 1	1.53	2.48	0.25	26.56	5.12	9.61	1.78
Association rule 2	1.18	1.89	0.16	21.98	4.51	8.54	1.48
Association rule 3	1.01	1.80	0.13	19.26	3.75	7.97	1.22
Association rule 4	1.66	2.58	0.27	28.91	5.34	10.53	1.98
**CD20⁺ B cell**							
Promoter	2.02	2.09	0.50	25.91	7.15	8.63	2.30
Association rule 1	1.66	2.39	0.30	25.03	5.54	7.74	1.60
Association rule 2	1.40	2.03	0.16	20.36	4.13	7.45	1.38
Association rule 3	1.10	1.73	0.15	17.65	3.69	6.86	1.20
Association rule 4	1.81	2.43	0.32	26.64	6.00	8.21	1.73
**HUVEC**							
Promoter	2.87	3.67	0.51	35.61	10.47	9.67	2.52
Association rule 1	2.24	3.96	0.25	36.05	6.46	13.64	2.34
Association rule 2	1.65	3.43	0.20	30.99	5.46	11.56	1.78
Association rule 3	1.61	2.87	0.16	27.53	4.58	10.63	1.70
Association rule 4	2.48	3.77	0.25	39.12	6.76	14.90	2.41
**IMR90**							
Promoter	3.29	3.74	1.06	38.00	6.88	13.66	2.68
Association rule 1	2.38	3.68	0.34	37.08	6.81	14.40	2.15
Association rule 2	1.81	3.03	0.13	29.59	5.56	12.40	1.86
Association rule 3	1.70	2.72	0.10	27.00	4.95	11.45	1.68
Association rule 4	2.18	4.17	0.30	38.45	6.99	14.42	2.43
**MCF-7**							
Promoter	2.92	3.02	0.77	39.68	11.43	12.97	3.59
Association rule 1	2.79	3.26	0.47	42.92	8.26	15.56	2.85
Association rule 2	2.32	2.72	0.23	34.80	7.67	13.21	2.38
Association rule 3	2.10	2.34	0.32	31.31	6.31	12.67	2.18
Association rule 4	2.98	3.45	0.61	46.90	10.08	16.14	3.25
**HMEC**							
Promoter	4.31	3.01	0.76	35.37	8.41	13.71	3.51
Association rule 1	2.59	3.68	0.34	36.27	7.74	12.74	2.88
Association rule 2	1.92	2.93	0.22	29.20	5.43	10.67	1.90
Association rule 3	1.64	2.92	0.14	26.56	4.94	10.07	1.77
Association rule 4	2.67	3.84	0.29	38.91	8.36	13.60	2.96
**H1-hESC**							
Promoter	3.21	2.28	0.72	33.48	6.75	11.67	2.46
Association rule 1	2.65	2.53	0.39	35.88	6.30	12.44	2.41
Association rule 2	2.14	2.68	0.28	31.31	6.58	10.70	1.97
Association rule 3	1.73	2.31	0.20	27.73	5.70	9.95	1.76
Association rule 4	2.98	2.77	0.43	39.09	7.26	13.27	2.61
**iPSC**							
Promoter	2.95	1.63	0.68	31.48	8.17	6.89	2.57
Association rule 1	2.58	2.26	0.37	31.23	7.53	6.90	2.43
Association rule 2	2.13	2.30	0.17	25.38	6.20	6.61	2.24
Association rule 3	1.92	2.13	0.21	23.32	6.58	6.50	1.80
Association rule 4	2.56	2.59	0.37	34.18	7.74	7.87	2.90

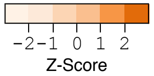

Number of functional enrichments of transcriptional target genes predicted using EPA

	KEGG	CTD Ontology	GO Slim	GO	Pathway Commons	Domains	Wiki Pathways
**Monocyte**							
Promoter	349	209	114	2,902	1,005	1,202	242
Association rule 1	720	447	139	9,213	3,087	2,911	766
Association rule 2	839	584	135	11,620	3,593	3,730	771
Association rule 3	837	549	142	11,073	2,519	4,047	792
Association rule 4	733	525	157	10,492	4,564	3,058	817
**CD4⁺ T cell**							
Promoter	317	278	77	4,077	1,806	1,401	405
Association rule 1	1,161	604	234	12,160	7,216	3,738	1,166
Association rule 2	1,436	708	323	14,939	9,140	4,903	1,272
Association rule 3	1,526	725	307	14,242	8,470	5,149	1,230
Association rule 4	1,265	606	311	13,697	8,338	4,155	1,290
**CD20⁺ B cell**							
Promoter	323	170	88	2,778	821	950	288
Association rule 1	584	408	170	6,874	3,900	2,258	545
Association rule 2	706	437	127	8,652	4,707	3,125	817
Association rule 3	852	499	134	8,402	2,505	3,326	781
Association rule 4	594	395	183	8,026	5,431	2,465	637
**HUVEC**							
Promoter	235	180	42	2,498	696	702	144
Association rule 1	602	464	194	8,318	1,923	3,376	460
Association rule 2	820	606	329	12,178	2,547	5,162	598
Association rule 3	759	594	351	11,881	2,644	5,705	597
Association rule 4	647	432	244	9,214	2,840	3,972	487
**IMR90**							
Promoter	131	103	48	1,350	230	504	108
Association rule 1	372	262	110	4,726	961	2,525	310
Association rule 2	520	366	80	6,434	913	3,368	417
Association rule 3	593	348	96	6,130	952	3,391	360
Association rule 4	382	278	136	5,144	1,088	2,710	318
**MCF-7**							
Promoter	136	106	33	1,519	482	507	171
Association rule 1	302	200	65	3,892	715	1,763	315
Association rule 2	416	272	39	4,923	951	2,374	351
Association rule 3	492	257	56	5,023	949	2,768	369
Association rule 4	319	218	82	4,422	922	1,844	327
**HMEC**							
Promoter	195	112	34	1,464	313	590	149
Association rule 1	450	291	81	5,718	756	3,000	334
Association rule 2	548	375	97	8,416	1,120	4,345	475
Association rule 3	614	429	111	8,152	1,140	4,270	512
Association rule 4	454	290	134	6,423	887	3,472	389
**H1-hESC**							
Promoter	234	96	69	2,030	391	674	134
Association rule 1	392	198	81	4,657	898	1,547	259
Association rule 2	579	313	91	6,644	1,145	2,059	288
Association rule 3	491	297	88	6,207	1,105	2,062	345
Association rule 4	397	202	123	5,317	1,321	1,736	253
**iPSC**							
Promoter	202	48	37	1,574	532	289	150
Association rule 1	358	197	58	3,755	641	935	300
Association rule 2	543	288	47	4,724	789	1,306	379
Association rule 3	604	356	52	4,695	858	1,286	401
Association rule 4	365	231	81	4,372	756	1,009	297

Number of unique functional enrichments of transcriptional target genes predicted using EPA

	KEGG	CTD Ontology	GO Slim	GO	Pathway Commons	Domains	Wiki Pathways
**Monocyte**							
Promoter	104	117	25	1,227	321	419	84
Association rule 1	141	194	18	1,841	364	638	137
Association rule 2	132	228	16	1,948	350	646	142
Association rule 3	133	197	12	1,972	351	721	142
Association rule 4	138	196	16	1,835	325	616	132
**CD4⁺ T cell**							
Promoter	105	146	23	1,654	415	585	133
Association rule 1	131	212	21	2,268	437	821	152
Association rule 2	125	200	17	2,326	477	904	157
Association rule 3	124	222	16	2,370	461	980	150
Association rule 4	128	199	21	2,230	412	812	153
**CD20⁺ B cell**							
Promoter	93	96	23	1,192	329	397	106
Association rule 1	109	157	20	1,646	364	509	105
Association rule 2	115	167	13	1,672	339	612	113
Association rule 3	104	164	14	1,669	349	649	113
Association rule 4	108	145	19	1,590	358	490	103
**HUVEC**							
Promoter	90	115	16	1,116	328	303	79
Association rule 1	116	205	13	1,868	335	707	121
Association rule 2	114	237	14	2,141	377	799	123
Association rule 3	124	221	12	2,123	353	820	131
Association rule 4	119	281	12	1,880	325	716	116
**IMR90**							
Promoter	65	74	21	751	136	270	53
Association rule 1	92	142	13	1,432	263	556	83
Association rule 2	101	169	7	1,651	310	692	104
Association rule 3	105	168	6	1,670	306	708	104
Association rule 4	79	151	11	1,392	253	522	88
**MCF-7**							
Promoter	57	59	15	774	223	253	70
Association rule 1	89	104	15	1,371	264	497	91
Association rule 2	102	120	10	1,533	338	582	105
Association rule 3	105	117	16	1,567	316	634	109
Association rule 4	88	102	18	1,386	298	477	96
**HMEC**							
Promoter	96	67	17	787	187	305	78
Association rule 1	107	152	14	1,500	320	527	119
Association rule 2	114	174	13	1,736	323	634	113
Association rule 3	108	193	9	1,754	326	665	117
Association rule 4	103	148	11	1,499	322	524	114

**H1-hESC**							
Promoter	89	63	20	927	187	323	68
Association rule 1	109	104	16	1,474	259	511	99
Association rule 2	115	144	15	1,685	354	576	106
Association rule 3	106	142	12	1,702	350	611	108
Association rule 4	112	104	16	1,470	273	499	98
**iPSC**							
Promoter	69	38	16	736	191	161	60
Association rule 1	90	79	13	1,091	263	241	85
Association rule 2	99	107	8	1,178	288	307	104
Association rule 3	99	110	11	1,205	340	336	93
Association rule 4	82	83	12	1,095	248	252	93

Table S14. Normalized number of functional enrichments of transcriptional target genes predicted from promoter and extended regions for enhancer-promoter association using ChIP-seq data of 19 TF in H1-hESC. * Wilcoxon signed-rank test $p < 0.01$

Table S14. Normalized number of functional enrichments of transcriptional target genes predicted from promoter and extended regions for enhancer-promoter association (EPA) using ChIP-seq data of 19 TF in H1-hESC. * Wilcoxon signed-rank test $p < 0.01$

	KEGG	TF Targets	CTD Ontology	GO Slim	GO	Pathway Commons	BioMarkers	MicroRNA	Domains	WikiPathways
Association rule 1	10.85	1.01	0.08	10.59	88.45	2.52	3.36	314.11	8.74	3.70
Association rule 2	6.65	0.22	0.00	6.20	59.53	0.37	1.55	141.51	3.40	2.14
Association rule 3	5.91	0.13	0.00	3.42	33.30	0.07	0.40	60.63	2.42	0.34
Association rule 4	11.48	0.95	0.09	10.27	91.49	2.24	3.88	352.83	9.58	3.54

-2 -1 0 1 2
Z-Score

Normalized number of unique functional enrichements of transcriptional target genes predicted from EPA.

	KEGG	TF Targets	CTD Ontology	GO Slim	GO	Pathway Commons	BioMarkers	MicroRNA	Domains	WikiPathways
Association rule 1	1.85	0.42	0.08	1.93	20.77	0.92	0.59	39.60	6.73	0.92
Association rule 2	1.18	0.22	0.00	1.48	16.32	0.07	0.59	32.87	2.88	0.44
Association rule 3	0.94	0.13	0.00	0.94	11.01	0.07	0.27	25.98	2.42	0.13
Association rule 4	1.81	0.35	0.09	1.90	23.13	0.69	0.69	40.74	6.99	0.86

Number of functional enrichments of transcriptional target genes predicted from EPA.

	KEGG	TF Targets	CTD Ontology	GO Slim	GO	Pathway Commons	BioMarkers	MicroRNA	Domains	WikiPathways
Association rule 1	129	12	1	126	1,052	30	40	3,736	104	44
Association rule 2	90	3	0	84	806	5	21	1,916	46	29
Association rule 3	88	2	0	51	496	1	6	903	36	5
Association rule 4	133	11	1	119	1,060	26	45	4,088	111	41

Number of unique functional enrichments of transcriptional target genes predicted from EPA.

	KEGG	TF Targets	CTD Ontology	GO Slim	GO	Pathway Commons	BioMarkers	MicroRNA	Domains	WikiPathways
Association rule 1	22	5	1	23	247	11	7	471	80	11
Association rule 2	16	3	0	20	221	1	8	445	39	6
Association rule 3	14	2	0	14	164	1	4	387	36	2
Association rule 4	21	4	1	22	268	8	8	472	81	10

Table S15. Differences of functional enrichments of putative transcriptional target genes between promoter and 'promoter and extended regions for enhancer-promoter association' using Pathway Commons (Fold change > 2).

Functional annotations enriched more in 'promoter and extended regions for enhancer-promoter association' than promoters in monocytes	No. of TF
Proteoglycan syndecan-mediated signaling events	61
Glypican pathway	56
Syndecan-1-mediated signaling events	54
Signaling events mediated by VEGFR1 and VEGFR2	53
LKB1 signaling events	52
IFN-gamma pathway	51
IL5-mediated signaling events	51
Plasma membrane estrogen receptor signaling	48
IL3-mediated signaling events	46
Alpha9 beta1 integrin signaling events	45
Nectin adhesion pathway	45
Sphingosine 1-phosphate (S1P) pathway	45
VEGF and VEGFR signaling network	44
Internalization of ErbB1	43
Signaling events mediated by Hepatocyte Growth Factor Receptor (c-Met)	43
Urokinase-type plasminogen activator (uPA) and uPAR-mediated signaling	43
Arf6 signaling events	43
EGFR-dependent Endothelin signaling events	43
IGF1 pathway	43
ErbB1 downstream signaling	43
Arf6 trafficking events	43
Signaling events mediated by focal adhesion kinase	43
Class I PI3K signaling events mediated by Akt	43
mTOR signaling pathway	43
Insulin Pathway	43
GMCSF-mediated signaling events	43
Arf6 downstream pathway	43
PDGFR-beta signaling pathway	43
PDGF receptor signaling network	43
EGF receptor (ErbB1) signaling pathway	43
Class I PI3K signaling events	43
S1P1 pathway	43
Glypican 1 network	40
Endothelins	38
Thrombin/protease-activated receptor (PAR) pathway	37
PAR1-mediated thrombin signaling events	37
ErbB receptor signaling network	35
chondroitin sulfate biosynthesis (late stages)	35
Beta1 integrin cell surface interactions	33
TRAIL signaling pathway	28
Integrins in angiogenesis	26
Osteopontin-mediated events	21
EPO signaling pathway	21
Posttranslational regulation of adherens junction stability and dissassembly	18
Thromboxane A2 receptor signaling	18
BCR signaling pathway	17
LPA receptor mediated events	16
Regulation of p38-alpha and p38-beta	16
p38 MAPK signaling pathway	16
Regulation of nuclear beta catenin signaling and target gene transcription	16
C-MYB transcription factor network	15
KitReceptor	15
BMP receptor signaling	15
Role of Calcineurin-dependent NFAT signaling in lymphocytes	14
Wnt signaling network	13
Netrin-mediated signaling events	13
Glypican 3 network	13
Neurotrophic factor-mediated Trk receptor signaling	12
EphrinB-EPHB pathway	11
TCR signaling	11
TCR signaling in naive CD4+ T cells	11
Syndecan-4-mediated signaling events	11
Signaling events mediated by TCPTP	11
IL1-mediated signaling events	11
chondroitin sulfate biosynthesis	10
S1P3 pathway	10
Fc-epsilon receptor I signaling in mast cells	10
Semaphorin interactions	10
N-cadherin signaling events	10
Hemostasis	9
Downstream TCR signaling	9
Toll Like Receptor 4 (TLR4) Cascade	9
IL2 signaling events mediated by PI3K	9
IL8-mediated signaling events	8
Stabilization and expansion of the E-cadherin adherens junction	8
Regulation of cytoplasmic and nuclear SMAD2/3 signaling	8
E-cadherin signaling in the nascent adherens junction	8
Regulation of nuclear SMAD2/3 signaling	8
Platelet activation, signaling and aggregation	8
GAB1 signalosome	8
Canonical Wnt signaling pathway	8
TGF-beta receptor signaling	8
Glucocorticoid receptor signaling	7
Trk receptor signaling mediated by PI3K and PLC-gamma	7
E-cadherin signaling events	7
Nongenotropic Androgen signaling	7
Reelin signaling pathway	7
Aurora A signaling	7
Glucocorticoid receptor regulatory network	7
Integrin cell surface interactions	7
Cell surface interactions at the vascular wall	6
Axon guidance	6
ErbB4 signaling events	6
Wnt	6
Ephrin B reverse signaling	5
Other semaphorin interactions	5
PDGFR-alpha signaling pathway	5
Toll Like Receptor TLR1:TLR2 Cascade	5
Toll Like Receptor 2 (TLR2) Cascade	5
p75(NTR)-mediated signaling	5
GPCR downstream signaling	5
Transcriptional Regulation of White Adipocyte Differentiation	5
ALK1 signaling events	5
C-MYC pathway	5
Syndecan-2-mediated signaling events	5
Angiopoietin receptor Tie2-mediated signaling	5
TGFBR	5
Signaling by Interleukins	5
Toll Like Receptor TLR6:TLR2 Cascade	5
FGF signaling pathway	5
ALK1 pathway	5
G alpha (q) signalling events	5
Regulation of IFNG signaling	5
Transmission across Chemical Synapses	4
Signaling events mediated by the Hedgehog family	4
Signaling by PDGF	4
Amino acid synthesis and interconversion (transamination)	4
MyD88:Mal cascade initiated on plasma membrane	4
VEGFR3 signaling in lymphatic endothelium	4
dermatan sulfate biosynthesis	4
NOTCH	4
Sumoylation by RanBP2 regulates transcriptional repression	4
EGFR1	4
L1CAM interactions	4
p63 transcription factor network	4
D-myo-inositol (1,3,4)-trisphosphate biosynthesis	4
Downstream signal transduction	4
BMAL1:CLOCK/NPAS2 Activates Gene Expression	4
Presenilin action in Notch and Wnt signaling	4
Validated targets of C-MYC transcriptional repression	3
S1P4 pathway	3
1D-myo-inositol hexakisphosphate biosynthesis II (mammalian)	3
Toll Like Receptor 5 (TLR5) Cascade	3
chondroitin and dermatan biosynthesis	3
Activated TLR4 signalling	3
TRAF6 mediated induction of NFkB and MAP kinases upon TLR7/8 or 9 activation	3
EPHB forward signaling	3
Toll Like Receptor 10 (TLR10) Cascade	3
MyD88 cascade initiated on plasma membrane	3
Regulation of Lipid Metabolism by Peroxisome proliferator-activated receptor alpha (PPA	3
Validated transcriptional targets of deltaNp63 isoforms	3

Functional annotations enriched more in promoters than EPA in monocytes	No. of TF
Interferon Signaling	13
Mitotic Prophase	11
Golgi Cisternae Pericentriolar Stack Reorganization	11
Interferon alpha/beta signaling	11
Recruitment of mitotic centrosome proteins and complexes	10
Centrosome maturation	10
ATR signaling pathway	10
Antigen processing-Cross presentation	9
IL23-mediated signaling events	9
Class I MHC mediated antigen processing & presentation	9
G2/M Transition	8
Loss of Nlp from mitotic centrosomes	8
ER-Phagosome pathway	8

Loss of proteins required for interphase microtubule organization from the centrosome	8
Mitotic G2-G2/M phases	7
Immune System	7
p53 pathway	6
Direct p53 effectors	6
Processing of Capped Intronless Pre-mRNA	6
Post-Elongation Processing of Intronless pre-mRNA	6
TNF signaling	6
HIF-1-alpha transcription factor network	6
Processing of Intronless Pre-mRNAs	5
Caspase cascade in apoptosis	5
Trk receptor signaling mediated by the MAPK pathway	5
FAS (CD95) signaling pathway	5
IL4-mediated signaling events	5
Alpha-synuclein signaling	5
M Phase	4
Alpha4 beta1 integrin signaling events	4
IL8- and CXCR1-mediated signaling events	4
Caspase-mediated cleavage of cytoskeletal proteins	4
ATM pathway	4
Clathrin derived vesicle budding	3
Regulation of ornithine decarboxylase (ODC)	3
Metabolism of polyamines	3
Nonsense Mediated Decay Independent of the Exon Junction Complex	3
trans-Golgi Network Vesicle Budding	3
Fatty acid, triacylglycerol, and ketone body metabolism	3
Interleukin-1 signaling	3
Hypoxic and oxygen homeostasis regulation of HIF-1-alpha	3
amb2 Integrin signaling	3

Functional annotations enriched more in EPA than promoters in CD4$^+$ T cells	No. of TF
TCR signaling in naive CD8$^+$ T cells	130
Integrin family cell surface interactions	112
Alpha9 beta1 integrin signaling events	111
Beta1 integrin cell surface interactions	111
IFN-gamma pathway	107
ErbB receptor signaling network	107
LKB1 signaling events	107
TCR signaling in naive CD4$^+$ T cells	103
GMCSF-mediated signaling events	103
Thrombin/protease-activated receptor (PAR) pathway	102
PAR1-mediated thrombin signaling events	102
Signaling events mediated by VEGFR1 and VEGFR2	101
Internalization of ErbB1	100
Urokinase-type plasminogen activator (uPA) and uPAR-mediated signaling	100
Arf6 signaling events	100
VEGF and VEGFR signaling network	100
EGFR-dependent Endothelin signaling events	100
ErbB1 downstream signaling	100
IL3-mediated signaling events	100
Arf6 trafficking events	100
Signaling events mediated by focal adhesion kinase	100
Class I PI3K signaling events mediated by Akt	100
mTOR signaling pathway	100
Insulin Pathway	100
Arf6 downstream pathway	100
PDGFR-beta signaling pathway	100
Nectin adhesion pathway	100
EGF receptor (ErbB1) signaling pathway	100
Class I PI3K signaling events	100
S1P1 pathway	100
IL5-mediated signaling events	99
Glypican 1 network	99
Proteoglycan syndecan-mediated signaling events	98
Signaling events mediated by Hepatocyte Growth Factor Receptor (c-Met)	98
Endothelins	98
IGF1 pathway	98
PDGF receptor signaling network	98
Glypican pathway	97
Plasma membrane estrogen receptor signaling	95
Sphingosine 1-phosphate (S1P) pathway	95
TRAIL signaling pathway	94
Syndecan-1-mediated signaling events	94
CXCR4-mediated signaling events	78
IL2-mediated signaling events	61
Signaling events mediated by TCPTP	56
Regulation of CDC42 activity	54
Angiopoietin receptor Tie2-mediated signaling	53
CDC42 signaling events	52
Integrin-linked kinase signaling	49
AP-1 transcription factor network	48
CD40/CD40L signaling	48
IL4-mediated signaling events	47
Signaling events mediated by HDAC Class II	40
IL2 signaling events mediated by PI3K	40
HIF-1-alpha transcription factor network	38
KitReceptor	36
EPO signaling pathway	35
BCR signaling pathway	33
Costimulation by the CD28 family	32
Osteopontin-mediated events	32
CD28 co-stimulation	31
C-MYB transcription factor network	28
LPA receptor mediated events	28
Signaling events mediated by HDAC Class I	28
1D-myo-inositol hexakisphosphate biosynthesis II (mammalian)	27
Fc-epsilon receptor I signaling in mast cells	27
IL1-mediated signaling events	27
D-myo-inositol (1,3,4)-trisphosphate biosynthesis	27
TNF receptor signaling pathway	26
Generation of second messenger molecules	25
IL23-mediated signaling events	25
Reelin signaling pathway	23
Stabilization and expansion of the E-cadherin adherens junction	20
E-cadherin signaling in the nascent adherens junction	20
IL6-mediated signaling events	20
Integrins in angiogenesis	20
E-cadherin signaling events	19
JNK signaling in the CD4$^+$ TCR pathway	19
Hypoxic and oxygen homeostasis regulation of HIF-1-alpha	18
EphrinA-EPHA pathway	17
E-cadherin signaling in keratinocytes	17
Signaling events mediated by Stem cell factor receptor (c-Kit)	17
Aurora A signaling	17
Transmission across Chemical Synapses	16
Notch-mediated HES/HEY network	16
Notch signaling pathway	16
Netrin-mediated signaling events	16
Neuronal System	16
Signaling by EGFR	16
Androgen-mediated signaling	16
Regulation of Androgen receptor activity	15
Role of Calcineurin-dependent NFAT signaling in lymphocytes	14
Phosphorylation of CD3 and TCR zeta chains	13
Signaling by GPCR	13
Ceramide signaling pathway	13
Alpha6Beta4Integrin	13
AndrogenReceptor	13
Ephrin B reverse signaling	12
PDGFR-alpha signaling pathway	12
superpathway of D-myo-inositol (1,4,5)-trisphosphate metabolism	12
Translocation of ZAP-70 to Immunological synapse	12
The role of Nef in HIV-1 replication and disease pathogenesis	12
EGFR downregulation	12
N-cadherin signaling events	12
p75 NTR receptor-mediated signalling	11
Semaphorin interactions	11
Regulation of p38-alpha and p38-beta	11
Syndecan-4-mediated signaling events	11
VEGFR1 specific signals	11
Signaling by SCF-KIT	11
Glucocorticoid receptor signaling	10
Signaling by FGFR	10
Signaling by TGF beta	10
PD-1 signaling	10
Glucocorticoid receptor regulatory network	10
Hemostasis	9
PLK1 signaling events	9
Downstream signaling of activated FGFR	9
EphrinB-EPHB pathway	8
Signaling by PDGF	8
Posttranslational regulation of adherens junction stability and dissassembly	8
Caspase cascade in apoptosis	8
Regulation of Telomerase	8
Signalling by NGF	8
Signaling events mediated by PTP1B	8
Polo-like kinase signaling events in the cell cycle	8
a4b7 Integrin signaling	7
VEGFR3 signaling in lymphatic endothelium	7
p38 MAPK signaling pathway	7
TGFBR	7
Activation of NMDA receptor upon glutamate binding and postsynaptic events	6
Direct p53 effectors	6
GPCR downstream signaling	6
Post NMDA receptor activation events	6
Retinoic acid receptors-mediated signaling	6
BMP receptor signaling	6
ErbB4 signaling events	6
Nephrin/Neph1 signaling in the kidney podocyte	5
Activation of Pro-Caspase 8	5
S1P4 pathway	5
TNF signaling	5
HIV-1 Nef: Negative effector of Fas and TNF-alpha	5
Platelet activation, signaling and aggregation	5
EPHA2 forward signaling	5
Caspase-8 is formed from procaspase-8	5

IL27-mediated signaling events	5
Signaling by Interleukins	5
Platelet homeostasis	5
Validated transcriptional targets of deltaNp63 isoforms	5
NCAM signaling for neurite out-growth	4
p38 signaling mediated by MAPKAP kinases	4
PLC beta mediated events	4
S1P3 pathway	4
G-protein mediated events	4
IL8- and CXCR1-mediated signaling events	4
EPHB forward signaling	4
Ca-dependent events	4
Sema4D in semaphorin signaling	4
Cell-Cell communication	4
Nucleotide-binding domain, leucine rich repeat containing receptor (NLR) signaling pathways	4
Downstream signal transduction	4
CD28 dependent Vav1 pathway	3
p53 pathway	3
NOD1/2 Signaling Pathway	3
GP1b-IX-V activation signalling	3
Apoptotic execution phase	3
Trk receptor signaling mediated by PI3K and PLC-gamma	3
Regulation of KIT signaling	3
Sema3A PAK dependent Axon repulsion	3
Neurotrophic factor-mediated Trk receptor signaling	3
Class B/2 (Secretin family receptors)	3
MAP kinase activation in TLR cascade	3
C-MYC pathway	3
Signaling by BMP	3
Axon guidance	3
Platelet Adhesion to exposed collagen	3
Opioid Signalling	3
HIF-2-alpha transcription factor network	3
p63 transcription factor network	3
Signal regulatory protein (SIRP) family interactions	3
NGF signalling via TRKA from the plasma membrane	3
Signaling events mediated by PRL	3
GPVI-mediated activation cascade	3
3-phosphoinositide biosynthesis	3

Functional annotations enriched more in promoters than EPA in CD4+ T cells	No. of TF
Transcription	12
Extrinsic Pathway for Apoptosis	12
Death Receptor Signalling	12
Processing of Intronless Pre-mRNAs	10
Antigen processing-Cross presentation	9
ER-Phagosome pathway	9
Circadian rhythm pathway	9
Class I MHC mediated antigen processing & presentation	8
Signaling by Wnt	7
Degradation of beta-catenin by the destruction complex	7
Cytokine Signaling in Immune system	7
Interferon Signaling	7
BMAL1:CLOCK/NPAS2 Activates Gene Expression	7
Regulation of Apoptosis	6
Circadian Clock	6
Netrin-1 signaling	6
Cell junction organization	6
Cyclin E associated events during G1/S transition	6
Interferon alpha/beta signaling	6
p53-Dependent G1 DNA Damage Response	5
Vpu mediated degradation of CD4	5
G2/M Transition	5
Autodegradation of the E3 ubiquitin ligase COP1	5
Glutamate Neurotransmitter Release Cycle	5
Cyclin A:Cdk2-associated events at S phase entry	5
Regulation of APC/C activators between G1/S and early anaphase	5
APC/C-mediated degradation of cell cycle proteins	5
Cdc20:Phospho-APC/C mediated degradation of Cyclin A	5
Activation of APC/C and APC/C:Cdc20 mediated degradation of mitotic proteins	5
p53-Independent DNA Damage Response	5
p53-Independent G1/S DNA damage checkpoint	5
APC/C:Cdc20 mediated degradation of mitotic proteins	5
Cross-presentation of soluble exogenous antigens (endosomes)	5
p53-Dependent G1/S DNA damage checkpoint	5
CDT1 association with the CDC6:ORC:origin complex	5
Assembly of the pre-replicative complex	5
APC/C:Cdh1 mediated degradation of Cdc20 and other APC/C:Cdh1 targeted proteins in late mitosis/early G1	5
Regulation of ornithine decarboxylase (ODC)	5
Antigen processing: Ubiquitination & Proteasome degradation	5
Vif-mediated degradation of APOBEC3G	5
G1/S DNA Damage Checkpoints	5
Stabilization of p53	5
CDK-mediated phosphorylation and removal of Cdc6	5
Mitotic G2-G2/M phases	5
Regulation of mitotic cell cycle	5
Ubiquitin-dependent degradation of Cyclin D1	5
SCF(Skp2)-mediated degradation of p27/p21	5
APC/C:Cdc20 mediated degradation of Securin	5
Ubiquitin-dependent degradation of Cyclin D	5
Autodegradation of Cdh1 by Cdh1:APC/C	5
Metabolism of lipids and lipoproteins	5
Regulation of activated PAK-2p34 by proteasome mediated degradation	5
Canonical Wnt signaling pathway	5
Toll Like Receptor 9 (TLR9) Cascade	5
Destabilization of mRNA by AUF1 (hnRNP D0)	5
Ubiquitin Mediated Degradation of Phosphorylated Cdc25A	5
Wnt	5
SCF-beta-TrCP mediated degradation of Emi1	5
Switching of origins to a post-replicative state	4
Negative regulators of RIG-I/MDA5 signaling	4
Insulin-mediated glucose transport	4
Regulation of DNA replication	4
DNA Replication Pre-Initiation	4
Orc1 removal from chromatin	4
M Phase	4
RNA Polymerase III Transcription Initiation From Type 2 Promoter	4
Removal of licensing factors from origins	4
M/G1 Transition	4
Factors involved in megakaryocyte development and platelet production	4
Atypical NF-kappaB pathway	4
RNA Polymerase III Transcription Initiation From Type 1 Promoter	4
Alternative NF-kappaB pathway	3
Regulation of mRNA Stability by Proteins that Bind AU-rich Elements	3
S Phase	3
G1 Phase	3
Regulation of retinoblastoma protein	3
RNA Polymerase III Transcription Initiation	3
Host Interactions of HIV factors	3
DNA Replication	3
The citric acid (TCA) cycle and respiratory electron transport	3
Intrinsic Pathway for Apoptosis	3
Cyclin D associated events in G1	3
NOTCH	3
RNA Polymerase III Abortive And Retractive Initiation	3
G1/S Transition	3
RNA Polymerase III Transcription	3
mitochondrial fatty acid beta-oxidation of unsaturated fatty acids	3
Nonsense-Mediated Decay	3
mRNA 3'-end processing	3
RNA Polymerase II Transcription	3
IL2 signaling events mediated by STAT5	3
Nonsense Mediated Decay Enhanced by the Exon Junction Complex	3
Cell Cycle, Mitotic	3
Post-Elongation Processing of Intron-Containing pre-mRNA	3
Formation and Maturation of mRNA Transcript	3

Functional annotations enriched more in EPA than promoters in CD20+ B cells	No. of TF
Beta1 integrin cell surface interactions	71
LKB1 signaling events	70
Syndecan-1-mediated signaling events	70
Glypican pathway	69
IFN-gamma pathway	69
Proteoglycan syndecan-mediated signaling events	69
Alpha9 beta1 integrin signaling events	69
Integrin family cell surface interactions	69
Signaling events mediated by Hepatocyte Growth Factor Receptor (c-Met)	68
VEGF and VEGFR signaling network	68
Thrombin/protease-activated receptor (PAR) pathway	66
PAR1-mediated thrombin signaling events	66
Signaling events mediated by VEGFR1 and VEGFR2	66
Endothelins	65
IL5-mediated signaling events	64
Internalization of ErbB1	63
Urokinase-type plasminogen activator (uPA) and uPAR-mediated signaling	63
Arf6 signaling events	63
EGFR-dependent Endothelin signaling events	63
IGF1 pathway	63
ErbB1 downstream signaling	63
Arf6 trafficking events	63
Signaling events mediated by focal adhesion kinase	63
Class I PI3K signaling events mediated by Akt	63
mTOR signaling pathway	63
Insulin Pathway	63
GMCSF-mediated signaling events	63
Arf6 downstream pathway	63
PDGFR-beta signaling pathway	63
PDGF receptor signaling network	63
Glypican 1 network	63
EGF receptor (ErbB1) signaling pathway	63
Class I PI3K signaling events	63
Sphingosine 1-phosphate (S1P) pathway	63
S1P1 pathway	63

ErbB receptor signaling network	62
Plasma membrane estrogen receptor signaling	61
TRAIL signaling pathway	60
Nectin adhesion pathway	60
IL3-mediated signaling events	59
Regulation of CDC42 activity	41
CDC42 signaling events	37
GPVI-mediated activation cascade	26
Noncanonical Wnt signaling pathway	23
Syndecan-4-mediated signaling events	23
Canonical Wnt signaling pathway	21
Wnt signaling network	20
EPO signaling pathway	20
Glypican 3 network	20
IL4-mediated signaling events	19
Signaling events mediated by TCPTP	18
AP-1 transcription factor network	17
Angiopoietin receptor Tie2-mediated signaling	17
Integrin-linked kinase signaling	16
Regulation of RAC1 activity	13
RAC1 signaling pathway	13
RhoA signaling pathway	13
Regulation of RhoA activity	13
Peptide ligand-binding receptors	12
Cytokine Signaling in Immune system	11
Osteopontin-mediated events	11
a6b1 and a6b4 Integrin signaling	11
Validated transcriptional targets of TAp63 isoforms	11
superpathway of D-myo-inositol (1,4,5)-trisphosphate metabolism	10
1D-myo-inositol hexakisphosphate biosynthesis II (mammalian)	10
Signaling events mediated by HDAC Class II	10
Thromboxane A2 receptor signaling	10
Integrins in angiogenesis	10
Calcineurin-regulated NFAT-dependent transcription in lymphocytes	10
D-myo-inositol (1,3,4)-trisphosphate biosynthesis	10
Signaling events mediated by PRL	10
GPCR ligand binding	9
Alpha-synuclein signaling	9
KitReceptor	8
Signaling by EGFR	8
NOD1/2 Signaling Pathway	7
Fatty acid, triacylglycerol, and ketone body metabolism	7
Netrin-mediated signaling events	7
Semaphorin interactions	7
Signaling events mediated by HDAC Class I	7
Class A/1 (Rhodopsin-like receptors)	7
Other semaphorin interactions	6
Cell surface interactions at the vascular wall	6
a4b7 Integrin signaling	6
Phase 1 - Functionalization of compounds	6
M Phase	6
Fc-epsilon receptor I signaling in mast cells	6
chondroitin sulfate biosynthesis (late stages)	6
Reelin signaling pathway	6
IL1-mediated signaling events	6
EphrinB-EPHB pathway	5
chondroitin sulfate biosynthesis	5
Axon guidance	5
HIF-1-alpha transcription factor network	5
Nucleotide-binding domain, leucine rich repeat containing receptor (NLR) signaling pathways	5
Presenilin action in Notch and Wnt signaling	5
Hemostasis	4
C-MYB transcription factor network	4
Toll Receptor Cascades	4
Neurotrophic factor-mediated Trk receptor signaling	4
TCR signaling	4
Signaling events regulated by Ret tyrosine kinase	4
Factors involved in megakaryocyte development and platelet production	4
Integrin cell surface interactions	4
G2/M Transition	3
Insulin-mediated glucose transport	3
Signaling by Aurora kinases	3
Mitotic Prometaphase	3
Alpha4 beta1 integrin signaling events	3
Mitotic G2-G2/M phases	3
Platelet activation, signaling and aggregation	3
Platelet Adhesion to exposed collagen	3
Aurora A signaling	3
Toll Like Receptor 10 (TLR10) Cascade	3
NGF signalling via TRKA from the plasma membrane	3

Functional annotations enriched more in promoters than EPA in CD20- B cells	No. of TF
Interferon alpha/beta signaling	12
Alpha6Beta4Integrin	11
Validated targets of C-MYC transcriptional activation	11
IL8- and CXCR2-mediated signaling events	10
Antigen processing-Cross presentation	9
ER-Phagosome pathway	8
Class I MHC mediated antigen processing & presentation	7
Regulation of cytoplasmic and nuclear SMAD2/3 signaling	6
Regulation of nuclear SMAD2/3 signaling	6
Interleukin-1 signaling	6
TCA cycle variation III (eukaryotic)	6
TGF-beta receptor signaling	6
Trk receptor signaling mediated by PI3K and PLC-gamma	5
ATF-2 transcription factor network	5
Mitotic Prophase	5
Golgi Cisternae Pericentriolar Stack Reorganization	5
Switching of origins to a post-replicative state	4
IL8-mediated signaling events	4
Loss of Nlp from mitotic centrosomes	4
GP1b-IX-V activation signalling	4
Apoptosis	4
Regulation of DNA replication	4
CDT1 association with the CDC6:ORC:origin complex	4
Assembly of the pre-replicative complex	4
Orc1 removal from chromatin	4
Metabolism of polyamines	4
Host Interactions of HIV factors	4
Nef and signal transduction	4
Loss of proteins required for interphase microtubule organization from the centrosome	4
Removal of licensing factors from origins	4
p53-Dependent G1 DNA Damage Response	3
Vpu mediated degradation of CD4	3
Adaptive Immune System	3
Autodegradation of the E3 ubiquitin ligase COP1	3
S Phase	3
Cyclin A:Cdk2-associated events at S phase entry	3
Regulation of APC/C activators between G1/S and early anaphase	3
Cdc20:Phospho-APC/C mediated degradation of Cyclin A	3
Signaling by Wnt	3
p53-Independent DNA Damage Response	3
p53-Independent G1/S DNA damage checkpoint	3
Degradation of beta-catenin by the destruction complex	3
Regulation of Apoptosis	3
Cross-presentation of soluble exogenous antigens (endosomes)	3
p53-Dependent G1/S DNA damage checkpoint	3
APC/C:Cdh1 mediated degradation of Cdc20 and other APC/C:Cdh1 targeted proteins in late mitosis/early G1	3
Regulation of ornithine decarboxylase (ODC)	3
Arf1 pathway	3
Antigen processing: Ubiquitination & Proteasome degradation	3
RIG-I/MDA5 mediated induction of IFN-alpha/beta pathways	3
Syndecan-2-mediated signaling events	3
G1/S DNA Damage Checkpoints	3
Stabilization of p53	3
CDK-mediated phosphorylation and removal of Cdc6	3
Ubiquitin-dependent degradation of Cyclin D1	3
Regulation of p38-alpha and p38-beta	3
SCF(Skp2)-mediated degradation of p27/p21	3
p38 MAPK signaling pathway	3
G1/S Transition	3
Ubiquitin-dependent degradation of Cyclin D	3
Cell Cycle Checkpoints	3
Autodegradation of Cdh1 by Cdh1:APC/C	3
Synthesis of DNA	3
Metabolism of lipids and lipoproteins	3
Cyclin E associated events during G1/S transition	3
Regulation of activated PAK-2p34 by proteasome mediated degradation	3
Destabilization of mRNA by AUF1 (hnRNP D0)	3
Ubiquitin Mediated Degradation of Phosphorylated Cdc25A	3
SCF-beta-TrCP mediated degradation of Emi1	3

Table S16. Total number, mean and median of putative transcriptional target genes.

Table S16. Total number, mean and median of putative transcriptional target genes

	Monocyte			$CD4^+$ T cell			$CD20^+$ B cell		
Region	Total number	Mean	Median	Total number	Mean	Median	Total number	Mean	Median
Association rule 4	639,038	164	53	771,437	197	54	596,900	160	35
CTCF (FR+RF+FF+RR)	483,874	129	35	571,761	150	39	497,523	144	27
CTCF (FR)	227,846	67	23	213,564	64	21	225,259	77	20

	HUVEC			IMR90			MCF-7		
Region	Total number	Mean	Median	Total number	Mean	Median	Total number	Mean	Median
Association rule 4	596,900	160	35	732,010	196	40	771,437	197	54
CTCF (FR+RF+FF+RR)	207,722	99	23	122,501	66	17	103,632	60	14
CTCF (FR)	152,462	74	21	120,799	64	16	98,469	58	14

	HMEC			H1-hESC			iPSC		
Region	Total number	Mean	Median	Total number	Mean	Median	Total number	Mean	Median
Association rule 4	596,900	160	35	732,010	196	40	771,437	197	54
CTCF (FR+RF+FF+RR)	135,160	71	17	171,464	92	17	154,297	95	15
CTCF (FR)	134,846	72	18	130,811	73	17	116,662	71	15

Table S17. Normalized number of unique functional enrichments of transcriptional target genes predicted using CTCF-binding sites.

Table S17. Normalized number of unique functional enrichments of transcriptional target genes predicted using CTCF-binding sites

	KEGG	CTD Ontology	GO Slim	GO	Pathway Commons	Domains	Wiki Pathways
**Monocyte**							
Association rule 4	2.16	3.07	0.25	28.72	5.09	9.64	2.07
CTCF (FR+RF+FF+RR)	2.60	3.70	0.45	33.27	6.68	11.33	2.31
CTCF (FR)	4.87	6.63	0.75	60.92	9.88	15.36	4.21
**$CD4^+$ T cell**							
Association rule 4	1.66	2.58	0.27	28.91	5.34	10.53	1.98
CTCF (FR+RF+FF+RR)	2.10	3.08	0.33	34.47	6.31	11.77	2.50
CTCF (FR)	5.01	5.34	0.66	61.29	10.72	15.78	4.07
**$CD20^+$ B cell**							
Association rule 4	1.81	2.43	0.32	26.64	6.00	8.21	1.73
CTCF (FR+RF+FF+RR)	2.11	2.59	0.50	28.32	7.52	9.31	2.15
CTCF (FR)	3.77	3.73	0.62	46.83	9.10	12.56	3.11
**HUVEC**							
Association rule 4	2.48	3.77	0.25	39.12	6.76	14.90	2.41
CTCF (FR+RF+FF+RR)	4.43	6.31	0.58	54.50	11.70	18.10	4.38
CTCF (FR)	5.05	6.23	0.66	82.97	11.94	25.06	4.66
**IMR90**							
Association rule 4	2.18	4.17	0.30	38.45	6.99	14.42	2.43
CTCF (FR+RF+FF+RR)	3.76	6.20	1.39	63.84	8.41	23.84	4.41
CTCF (FR)	4.30	6.79	0.83	76.49	9.69	26.74	5.55
**MCF-7**							
Association rule 4	2.98	3.45	0.61	46.90	10.08	16.14	3.25
CTCF (FR+RF+FF+RR)	4.63	4.15	1.54	75.65	13.90	23.06	5.79
CTCF (FR)	6.30	6.50	1.93	91.81	13.91	29.76	7.52
**HMEC**							
Association rule 4	2.67	3.84	0.29	38.91	8.36	13.60	2.96
CTCF (FR+RF+FF+RR)	4.59	5.99	1.33	62.67	12.65	20.27	4.81
CTCF (FR)	5.78	6.67	1.26	75.57	9.79	20.62	5.27
**H1-hESC**							
Association rule 4	2.98	2.77	0.43	39.09	7.26	13.27	2.61
CTCF (FR+RF+FF+RR)	4.43	3.91	0.73	50.27	11.66	16.68	3.85
CTCF (FR)	3.82	5.73	1.15	71.17	13.15	21.25	5.50
**iPSC**							
Association rule 4	2.56	2.59	0.37	34.18	7.74	7.87	2.90
CTCF (FR+RF+FF+RR)	4.73	2.07	1.17	44.98	11.02	8.23	3.56
CTCF (FR)	5.83	4.03	1.11	58.63	12.94	13.63	5.23

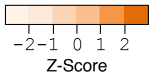

Number of functional enrichments of transcriptional target genes using predicted CTCF-binding sites

	KEGG	CTD Ontology	GO Slim	GO	Pathway Commons	Domains	Wiki Pathways
**Monocyte**							
Association rule 4	733	525	157	10,492	4,564	3,058	817
CTCF (FR+RF+FF+RR)	561	391	129	5,969	1,693	1,941	524
CTCF (FR)	978	445	129	11,617	4,479	2,555	800
**CD4+ T cell**							
Association rule 4	1,265	606	311	13,697	8,338	4,155	1,290
CTCF (FR+RF+FF+RR)	1,124	484	244	10,562	6,833	2,713	1,091
CTCF (FR)	1,482	313	532	11,969	4,710	2,846	994
**CD20+ B cell**							
Association rule 4	594	395	183	8,026	5,431	2,465	637
CTCF (FR+RF+FF+RR)	454	268	115	4,233	1,474	1,352	382
CTCF (FR)	650	219	149	6,848	4,973	2,250	511
**HUVEC**							
Association rule 4	647	432	244	9,214	2,840	3,972	487
CTCF (FR+RF+FF+RR)	402	220	80	3,222	589	1,202	282
CTCF (FR)	514	226	249	6,929	943	2,491	448
**IMR90**							
Association rule 4	382	278	136	5,144	1,088	2,710	318
CTCF (FR+RF+FF+RR)	170	215	113	3,090	489	1,509	275
CTCF (FR)	365	192	137	4,861	647	2,183	452
**MCF-7**							
Association rule 4	319	218	82	4,422	922	1,844	327
CTCF (FR+RF+FF+RR)	118	86	51	2,430	315	1,313	145
CTCF (FR)	220	113	60	3,916	506	1,432	370
**HMEC**							
Association rule 4	454	290	134	6,423	887	3,472	389
CTCF (FR+RF+FF+RR)	266	183	58	3,142	378	1,436	133
CTCF (FR)	399	195	94	5,265	592	1,683	415
**H1-hESC**							
Association rule 4	397	202	123	5,317	1,321	1,736	253
CTCF (FR+RF+FF+RR)	222	92	42	2,254	507	757	190
CTCF (FR)	369	138	118	3,969	745	1,824	258
**iPSC**							
Association rule 4	365	231	81	4,372	756	1,009	297
CTCF (FR+RF+FF+RR)	265	36	26	1,865	395	325	99
CTCF (FR)	431	71	80	3,200	366	973	196

Number of unique functional enrichments of transcriptional target genes predicted using CTCF-binding sites

	KEGG	CTD Ontology	GO Slim	GO	Pathway Commons	Domains	Wiki Pathways
**Monocyte**							
Association rule 4	138	196	16	1,835	325	616	132
CTCF (FR+RF+FF+RR)	126	179	22	1,610	323	548	112
CTCF (FR)	111	151	17	1,388	225	350	96
**CD4+ T cell**							
Association rule 4	128	199	21	2,230	412	812	153
CTCF (FR+RF+FF+RR)	120	176	19	1,971	361	673	143
CTCF (FR)	107	114	14	1,309	229	337	87
**CD20+ B cell**							
Association rule 4	108	145	19	1,590	358	490	103
CTCF (FR+RF+FF+RR)	105	129	25	1,409	374	463	107
CTCF (FR)	85	84	14	1,055	205	283	70
**HUVEC**							
Association rule 4	119	281	12	1,880	325	716	116
CTCF (FR+RF+FF+RR)	92	131	12	1,132	243	376	91
CTCF (FR)	77	95	10	1,265	182	382	71
**IMR90**							
Association rule 4	79	151	11	1,392	253	522	88
CTCF (FR+RF+FF+RR)	46	76	17	782	103	292	54
CTCF (FR)	52	82	10	924	117	323	67
**MCF-7**							
Association rule 4	88	102	18	1,386	298	477	96
CTCF (FR+RF+FF+RR)	48	43	16	784	144	239	60
CTCF (FR)	62	64	19	904	137	293	74
**HMEC**							
Association rule 4	103	148	11	1,499	322	524	114
CTCF (FR+RF+FF+RR)	62	81	18	847	171	274	65
CTCF (FR)	78	90	17	1,019	132	278	71
**H1-hESC**							
Association rule 4	112	104	16	1,470	273	499	98
CTCF (FR+RF+FF+RR)	76	67	13	862	200	286	66
CTCF (FR)	50	75	15	931	172	278	72
**iPSC**							
Association rule 4	82	83	12	1,095	248	252	93
CTCF (FR+RF+FF+RR)	73	32	18	694	170	127	55
CTCF (FR)	68	47	13	684	151	159	61

Table S18. Differences of functional enrichments of transcriptional target genes between 'promoter and extended regions for enhancer-promoter association (EPA)' (association rule 4) and EPA shortened at forward–reverse orientation of CTCF-binding sites using Pathway Commons (Fold change > 2) .

Functional annotations enriched more in promoter and extended regions for enahncer-promoter association (EPA) that were shortened at forward–reverse orientation of CTCF-binding sites in monocytes	No. of TF
CXCR4-mediated signaling events	33
GPVI-mediated activation cascade	26
Signaling events mediated by TCPTP	23
p38 MAPK signaling pathway	16
Interferon gamma signaling	15
G alpha (q) signalling events	12
TCR signaling in naive CD8$^+$ T cells	11
IL4-mediated signaling events	11
IL1-mediated signaling events	10
Signaling by GPCR	10
Immune System	10
FoxO family signaling	9
Platelet Adhesion to exposed collagen	9
IL2-mediated signaling events	9
RhoA signaling pathway	8
Regulation of RAC1 activity	8
Regulation of RhoA activity	8
RAC1 signaling pathway	8
Interferon Signaling	7
Toll Like Receptor 7/8 (TLR7/8) Cascade	6
MyD88 dependent cascade initiated on endosome	6
Signaling events mediated by Stem cell factor receptor (c-Kit)	6
Interleukin-1 signaling	6
Innate Immune System	5
Regulation of cytoplasmic and nuclear SMAD2/3 signaling	5
ALK1 pathway	5
Regulation of nuclear SMAD2/3 signaling	5
ALK1 signaling events	5
TGF-beta receptor signaling	5
IL8- and CXCR1-mediated signaling events	4
JNK signaling in the CD4$^+$ TCR pathway	4
Toll Like Receptor 9 (TLR9) Cascade	4
Metabolism of nucleotides	3
Fatty acid, triacylglycerol, and ketone body metabolism	3
Circadian rhythm pathway	3
Cell-Cell communication	3

Functional annotations enriched more in EPA in monocytes	No. of TF
chondroitin sulfate biosynthesis (late stages)	45
Integrin-linked kinase signaling	29
Osteopontin-mediated events	26
Netrin-mediated signaling events	16
Thromboxane A2 receptor signaling	15
Role of Calcineurin-dependent NFAT signaling in lymphocytes	14
CD40/CD40L signaling	14
LPA receptor mediated events	12
Toll Like Receptor 4 (TLR4) Cascade	12
EphrinB-EPHB pathway	11
C-MYB transcription factor network	11
Fc-epsilon receptor I signaling in mast cells	11
S1P3 pathway	10
Downstream TCR signaling	10
Canonical NF-kappaB pathway	10
chondroitin sulfate biosynthesis	9
Validated nuclear estrogen receptor alpha network	9
IL2 signaling events mediated by PI3K	9
Signaling by Interleukins	8
HIF-1-alpha transcription factor network	8
GAB1 signalosome	8
Cytokine Signaling in Immune system	7
IL6-mediated signaling events	7
Aurora A signaling	7
p53 pathway	6
Glucocorticoid receptor signaling	6
Cell surface interactions at the vascular wall	6
Recruitment of mitotic centrosome proteins and complexes	6
Syndecan-2-mediated signaling events	6
Signaling by EGFR	6
Nongenotropic Androgen signaling	6
Angiopoietin receptor Tie2-mediated signaling	6
Centrosome maturation	6
Signaling events regulated by Ret tyrosine kinase	6
Glucocorticoid receptor regulatory network	6
Wnt	6
Ephrin B reverse signaling	5
Vif-mediated degradation of APOBEC3G	5
ATF-2 transcription factor network	5
TNF alpha/NF-kB	5
NOTCH	5
FGF signaling pathway	5
ErbB4 signaling events	5
Validated targets of C-MYC transcriptional repression	4
PDGFR-alpha signaling pathway	4
Transmission across Chemical Synapses	4
NOD1/2 Signaling Pathway	4
Signaling by FGFR	4
Signaling by PDGF	4
Transcriptional Regulation of White Adipocyte Differentiation	4
Amino acid synthesis and interconversion (transamination)	4
Circadian Clock	4
Downstream signaling of activated FGFR	4
p63 transcription factor network	4
D-myo-inositol (1,3,4)-trisphosphate biosynthesis	4
Downstream signal transduction	4
Presenilin action in Notch and Wnt signaling	4
p38 signaling mediated by MAPKAP kinases	3
Purine catabolism	3
IL8- and CXCR2-mediated signaling events	3
Signaling events mediated by the Hedgehog family	3
S1P4 pathway	3
Signaling by Insulin receptor	3
C-MYC pathway	3
Nef and signal transduction	3
chondroitin and dermatan biosynthesis	3
dermatan sulfate biosynthesis	3
Endogenous TLR signaling	3
Beta3 integrin cell surface interactions	3
EPHB forward signaling	3
L1CAM interactions	3
NGF signalling via TRKA from the plasma membrane	3
Neurotransmitter Receptor Binding And Downstream Transmission In The Postsynaptic Cell	3
Validated transcriptional targets of deltaNp63 isoforms	3

Functional annotations enriched more in EPA shortened at forward–reverse orientation of CTCF-binding sites in CD4$^+$ T cells	No. of TF
TCR signaling in naive CD8$^+$ T cells	311
CXCR4-mediated signaling events	181
Fc-epsilon receptor I signaling in mast cells	85
JNK signaling in the CD4$^+$ TCR pathway	65
Reelin signaling pathway	62
TCR signaling	56
Semaphorin interactions	51
superpathway of D-myo-inositol (1,4,5)-trisphosphate metabolism	32
TGFBR	23
VEGFR3 signaling in lymphatic endothelium	20
Validated transcriptional targets of TAp63 isoforms	14
Cell surface interactions at the vascular wall	12
p63 transcription factor network	10
PLC-gamma1 signalling	10
Signal Transduction	10
Regulation of cytoplasmic and nuclear SMAD2/3 signaling	7
ALK1 pathway	7
Regulation of nuclear SMAD2/3 signaling	7
ALK1 signaling events	7
TGF-beta receptor signaling	7

Alternative NF-kappaB pathway	6
p75NTR signals via NF-kB	6
p73 transcription factor network	4
GAB1 signalosome	4
Axon guidance	3
G2/M Transition	3
Mitotic G2-G2/M phases	3

Functional annotations enriched more in EPA in CD4$^+$ T cells	No. of TF
LKB1 signaling events	141
IFN-gamma pathway	131
Alpha9 beta1 integrin signaling events	131
TRAIL signaling pathway	131
Integrin family cell surface interactions	130
Signaling events mediated by Hepatocyte Growth Factor Receptor (c-Met)	129
VEGF and VEGFR signaling network	127
Endothelins	127
Signaling events mediated by VEGFR1 and VEGFR2	127
Syndecan-1-mediated signaling events	127
IGF1 pathway	126
PDGF receptor signaling network	126
Sphingosine 1-phosphate (S1P) pathway	126
Thrombin/protease-activated receptor (PAR) pathway	125
PAR1-mediated thrombin signaling events	125
GMCSF-mediated signaling events	125
Plasma membrane estrogen receptor signaling	125
Beta1 integrin cell surface interactions	125
ErbB receptor signaling network	124
IL3-mediated signaling events	124
IL5-mediated signaling events	124
Glypican 1 network	124
Internalization of ErbB1	123
Proteoglycan syndecan-mediated signaling events	123
Urokinase-type plasminogen activator (uPA) and uPAR-mediated signaling	123
Arf6 signaling events	123
EGFR-dependent Endothelin signaling events	123
ErbB1 downstream signaling	123
Arf6 trafficking events	123
Signaling events mediated by focal adhesion kinase	123
Class I PI3K signaling events mediated by Akt	123
mTOR signaling pathway	123
Insulin Pathway	123
Arf6 downstream pathway	123
PDGFR-beta signaling pathway	123
EGF receptor (ErbB1) signaling pathway	123
Class I PI3K signaling events	123
S1P1 pathway	123
Regulation of CDC42 activity	93
CDC42 signaling events	81
Integrin-linked kinase signaling	64
Signaling events mediated by TCPTP	64
IL2-mediated signaling events	61
AP-1 transcription factor network	57
IL4-mediated signaling events	54
IL12-mediated signaling events	48
Signaling events mediated by HDAC Class II	44
IL1-mediated signaling events	43
HIF-1-alpha transcription factor network	41
IL2 signaling events mediated by PI3K	35
IL23-mediated signaling events	32
C-MYB transcription factor network	32
TNF receptor signaling pathway	32
Canonical NF-kappaB pathway	31
LPA receptor mediated events	30
Signaling events mediated by HDAC Class I	30
Hypoxic and oxygen homeostasis regulation of HIF-1-alpha	22
IL6-mediated signaling events	21
Endogenous TLR signaling	20
Aurora A signaling	20
IL12 signaling mediated by STAT4	19
Transmission across Chemical Synapses	18
Notch-mediated HES/HEY network	18
Notch signaling pathway	18
Neuronal System	18
p38 MAPK signaling pathway	18
Posttranslational regulation of adherens junction stability and dissassembly	17
EGFR downregulation	17
Generation of second messenger molecules	16
Syndecan-4-mediated signaling events	16
Signaling by TGF beta	14
Ephrin B reverse signaling	13
Regulation of RAC1 activity	13
Signaling by GPCR	13
RAC1 signaling pathway	13
RhoA signaling pathway	13
The role of Nef in HIV-1 replication and disease pathogenesis	13
Regulation of RhoA activity	13
Netrin-mediated signaling events	12
Noncanonical Wnt signaling pathway	11
Regulation of Androgen receptor activity	11
Glucocorticoid receptor signaling	11
Downstream TCR signaling	11
ATF-2 transcription factor network	11
Glucocorticoid receptor regulatory network	11
N-cadherin signaling events	11
IL8-mediated signaling events	10
E-cadherin signaling in keratinocytes	10
NCAM signaling for neurite out-growth	9
Caspase cascade in apoptosis	9
PLK1 signaling events	9
Signaling by SCF-KIT	9
Regulation of nuclear beta catenin signaling and target gene transcription	9
Androgen-mediated signaling	9
Signaling by FGFR	8
Signaling by PDGF	8
Translocation of ZAP-70 to Immunological synapse	8
Polo-like kinase signaling events in the cell cycle	8
AndrogenReceptor	8
Calcium signaling in the CD4$^+$ TCR pathway	7
Regulation of Telomerase	7
Transcription from mitochondrial promoters	7
Downstream signaling of activated FGFR	7
Retinoic acid receptors-mediated signaling	7
Signaling by Interleukins	7
GPVI-mediated activation cascade	7
EphrinB-EPHB pathway	6
Innate Immune System	6
Direct p53 effectors	6
a4b7 Integrin signaling	6
Trk receptor signaling mediated by PI3K and PLC-gamma	6
Calmodulin induced events	6
Cytokine Signaling in Immune system	6
CaM pathway	6
VEGFR1 specific signals	6
ErbB4 signaling events	6
Canonical Wnt signaling pathway	6
Validated targets of C-MYC transcriptional repression	5
IL8- and CXCR2-mediated signaling events	5
Activation of NMDA receptor upon glutamate binding and postsynaptic events	5
p53 pathway	5
NOD1/2 Signaling Pathway	5
Activation of Pro-Caspase 8	5
S1P4 pathway	5
Post NMDA receptor activation events	5
Caspase-8 is formed from procaspase-8	5
Signaling events mediated by PTP1B	5
IL27-mediated signaling events	5
Platelet homeostasis	5
Validated targets of C-MYC transcriptional activation	5
Validated transcriptional targets of deltaNp63 isoforms	5
p38 signaling mediated by MAPKAP kinases	4
Transport of Mature mRNA derived from an Intron-Containing Transcript	4
Signaling events mediated by the Hedgehog family	4
PLC beta mediated events	4
G-protein mediated events	4
Neurotrophic factor-mediated Trk receptor signaling	4
MAP kinase activation in TLR cascade	4
Extrinsic Pathway for Apoptosis	4
Death Receptor Signalling	4
C-MYC pathway	4
Nef and signal transduction	4
Signaling events mediated by HDAC Class III	4
Trk receptor signaling mediated by the MAPK pathway	4
IL8- and CXCR1-mediated signaling events	4
Immunoregulatory interactions between a Lymphoid and a non-Lymphoid cell	4
SEMA3A-Plexin repulsion signaling by inhibiting Integrin adhesion	4
Sumoylation by RanBP2 regulates transcriptional repression	4
Ca-dependent events	4
Transport of Mature Transcript to Cytoplasm	4
Nucleotide-binding domain, leucine rich repeat containing receptor (NLR) signaling pathways	4
Downstream signal transduction	4
IL2 signaling events mediated by STAT5	4
BMAL1:CLOCK/NPAS2 Activates Gene Expression	4
RXR and RAR heterodimerization with other nuclear receptor	3
CD28 dependent Vav1 pathway	3
Apoptotic execution phase	3
Interferon gamma signaling	3
superpathway of methionine degradation	3
Regulation of KIT signaling	3
Class B/2 (Secretin family receptors)	3
Lissencephaly gene (LIS1) in neuronal migration and development	3

Signaling by BMP	3
EPHA2 forward signaling	3
Meiosis	3
Coregulation of Androgen receptor activity	3
HIF-2-alpha transcription factor network	3
Signal regulatory protein (SIRP) family interactions	3
Activation of the pre-replicative complex	3
Toll Like Receptor 9 (TLR9) Cascade	3
Signaling events mediated by PRL	3
3-phosphoinositide biosynthesis	3

Functional annotations enriched more in EPA shortened at forward-reverse orientation of CTCF-binding sites in $CD20^+$ T cells	No. of TF
Syndecan-4-mediated signaling events	85
CXCR4-mediated signaling events	77
EPO signaling pathway	46
Posttranslational regulation of adherens junction stability and dissassembly	33
Stabilization and expansion of the E-cadherin adherens junction	32
E-cadherin signaling in the nascent adherens junction	32
E-cadherin signaling events	32
N-cadherin signaling events	21
TCR signaling in naive $CD8^+$ T cells	18
Interleukin-1 signaling	13
TCR signaling in naive $CD4^+$ T cells	12
Hemostasis	11
Interferon gamma signaling	11
Glycogen breakdown (glycogenolysis)	10
VEGFR3 signaling in lymphatic endothelium	7
NOTCH	7
ID	7
PLC-gamma1 signalling	7
FoxO family signaling	6
PLC beta mediated events	5
EGFR interacts with phospholipase C-gamma	5
Phospholipase C-mediated cascade	5
Calmodulin induced events	5
Ca-dependent events	5
G-protein mediated events	5
CaM pathway	5
Interleukin receptor SHC signaling	5
DAG and IP3 signaling	5
ATF-2 transcription factor network	4
Hypoxic and oxygen homeostasis regulation of HIF-1-alpha	4
Signalling by NGF	4
p53 pathway	3
Pyruvate metabolism and Citric Acid (TCA) cycle	3
Immune System	3

Functional annotations enriched more in EPA in $CD20^+$ B cells	No. of TF
Signaling events mediated by HDAC Class II	28
Role of Calcineurin-dependent NFAT signaling in lymphocytes	21
Angiopoietin receptor Tie2-mediated signaling	17
IL1-mediated signaling events	16
Regulation of p38-alpha and p38-beta	14
Peptide ligand-binding receptors	14
LPA receptor mediated events	13
Signaling events mediated by PRL	13
Signaling by GPCR	12
Validated transcriptional targets of TAp63 isoforms	12
Calcineurin-regulated NFAT-dependent transcription in lymphocytes	12
p38 MAPK signaling pathway	11
NOD1/2 Signaling Pathway	10
Thromboxane A2 receptor signaling	10
BMP receptor signaling	10
GPCR ligand binding	9
Transmission across Chemical Synapses	8
Fc-epsilon receptor I signaling in mast cells	8
Neuronal System	8
Signaling by EGFR	8
chondroitin sulfate biosynthesis (late stages)	8
IL2-mediated signaling events	8
Validated targets of C-MYC transcriptional activation	8
IL12-mediated signaling events	8
C-MYB transcription factor network	7
p75(NTR)-mediated signaling	7
CD40/CD40L signaling	7
Class A/1 (Rhodopsin-like receptors)	7
Presenilin action in Notch and Wnt signaling	7
Notch-mediated HES/HEY network	6
Phase 1 - Functionalization of compounds	6
Notch signaling pathway	6
Netrin-mediated signaling events	6
Reelin signaling pathway	6
IL2 signaling events mediated by PI3K	6
EphrinB-EPHB pathway	5
Activation of APC/C and APC/C:Cdc20 mediated degradation of mitotic proteins	5
APC/C:Cdc20 mediated degradation of mitotic proteins	5
IL6-mediated signaling events	5
Developmental Biology	5
APC/C:Cdc20 mediated degradation of Securin	5
Nucleotide-binding domain, leucine rich repeat containing receptor (NLR) signaling pathways	5
chondroitin sulfate biosynthesis	4
IL8-mediated signaling events	4
APC/C-mediated degradation of cell cycle proteins	4
Signaling by Aurora kinases	4
Regulation of cytoplasmic and nuclear SMAD2/3 signaling	4
Toll Receptor Cascades	4
M Phase	4
Regulation of nuclear SMAD2/3 signaling	4
C-MYC pathway	4
Regulation of mitotic cell cycle	4
IL8- and CXCR1-mediated signaling events	4
a6b1 and a6b4 Integrin signaling	4
Interleukin-3, 5 and GM-CSF signaling	4
Antigen Presentation: Folding, assembly and peptide loading of class I MHC	4
Polo-like kinase signaling events in the cell cycle	4
Signaling events regulated by Ret tyrosine kinase	4
Factors involved in megakaryocyte development and platelet production	4
TGF-beta receptor signaling	4
G2/M Transition	3
IL8- and CXCR2-mediated signaling events	3
Interleukin-2 signaling	3
PLK1 signaling events	3
Apoptotic cleavage of cellular proteins	3
Mitotic G2-G2/M phases	3
Platelet Adhesion to exposed collagen	3
Signaling events mediated by Stem cell factor receptor (c-Kit)	3
Toll Like Receptor 10 (TLR10) Cascade	3
Biological oxidations	3
Syndecan-3-mediated signaling events	3
EGFR downregulation	3

Table S19. Comparison of expression levels of transcriptional target genes predicted using promoters and 'promoter and extended regions for enhancer-promoter association' in monocytes, $CD4^+$ T cells, H1-hESC and iPSC.

Table S19. Comparison of expression levels of transcriptional target genes predicted using promoters and 'promoter and extended regions for enhancer-promoter association (EPA)' in monocytes, $CD4^+$ T cells, H1-hESC, and iPSC.

	Total no. of transcription factor binding sequences	Significant change of distribution of expression levels (high expression, Red dots)		Significant change of distribution of expression levels (low expression, Blue dots)		Sum of median expression levels using promoters (a)	Sum of median expression levels using EPA (b)	Ratio (b) / (a)
		No.	%	No.	%			
Monocyte								
Association rule 4	1948	13	0.7	42	2.2	43391	36549	0.84
CTCF (FR+RF+FF+RR)	1881	4	0.2	23	1.2	42040	34016	0.81
CTCF (FR)	1947	96	4.9	6	0.3	36263	40589	1.12
$CD4^+$ T cell								
Association rule 4	3597	44	1.2	5	0.1	24403	26125	1.07
CTCF (FR+RF+FF+RR)	3484	262	7.5	1	0.0	23415	28647	1.22
CTCF (FR)	3178	168	5.3	8	0.3	21170	27020	1.28
H1-hESC								
Association rule 4	1686	29	1.7	266	15.8	8087	5047	0.62
CTCF (FR+RF+FF+RR)	1676	63	3.8	237	14.1	7941	6173	0.78
CTCF (FR)	2045	0	0.0	445	21.8	9677	5792	0.60
iPSC								
Association rule 4	3085	2	0.1	281	9.1	8730	6690	0.77
CTCF (FR+RF+FF+RR)	2942	1	0.0	327	11.1	8299	6450	0.78
CTCF (FR)	2904	3	0.1	632	21.8	8093	5334	0.66
